# Potential inhibitors for SARS-CoV-2 Mpro from marine compounds†

**DOI:** 10.1039/d1ra03852d

**Published:** 2021-06-23

**Authors:** Nguyen Minh Tam, Minh Quan Pham, Huy Truong Nguyen, Nam Dao Hong, Nguyen Khoa Hien, Duong Tuan Quang, Huong Thi Thu Phung, Son Tung Ngo

**Affiliations:** Computational Chemistry Research Group, Ton Duc Thang University Ho Chi Minh City Vietnam; Faculty of Applied Sciences, Ton Duc Thang University Ho Chi Minh City Vietnam; Graduate University of Science and Technology, Vietnam Academy of Science and Technology Hanoi Vietnam; Institute of Natural Products Chemistry, Vietnam Academy of Science and Technology Hanoi Vietnam; Faculty of Pharmacy, Ton Duc Thang University Ho Chi Minh City Vietnam; University of Medicine and Pharmacy at Ho Chi Minh City Ho Chi Minh City Vietnam; Mientrung Institute for Scientific Research, Vietnam Academy of Science and Technology Hue City Thua Thien Hue Province Vietnam; Department of Chemistry, Hue University Hue City Thua Thien Hue Province Vietnam; NTT Hi-Tech Institute, Nguyen Tat Thanh University Ho Chi Minh City Vietnam; Laboratory of Theoretical and Computational Biophysics, Ton Duc Thang University Ho Chi Minh City Vietnam ngosontung@tdtu.edu.vn

## Abstract

Preventing the biological activity of SARS-CoV-2 main protease using natural compounds is of great interest. In this context, using a combination of AutoDock Vina and fast pulling of ligand simulations, eleven marine fungi compounds were identified that probably play as highly potent inhibitors for preventing viral replication. In particular, four compounds including M15 (3-*O*-(6-*O*-α-l-arabinopyranosyl)-β-d-glucopyranosyl-1,4-dimethoxyxanthone), M8 (*wailupemycins H*), M11 (cottoquinazolines B), and M9 (*wailupemycins I*) adopted the predicted ligand-binding free energy of −9.87, −9.82, −9.62, and −9.35 kcal mol^−1^, respectively, whereas the other adopted predicted ligand-binding free energies in the range from −8.54 to −8.94 kcal mol^−1^. The results were obtained using a combination of Vina and FPL simulations. Notably, although, AutoDock4 adopted higher accurate results in comparison with Vina, Vina is proven to be a more suitable technique for rapidly screening ligand-binding affinity with a large database of compounds since it requires much smaller computing resources. Furthermore, FPL is better than Vina to classify inhibitors upon ROC-AUC analysis.

## Abstract 2

Preventing the biological activity of SARS-CoV-2 main protease using natural compounds is of great interest.

## Graphical abstract

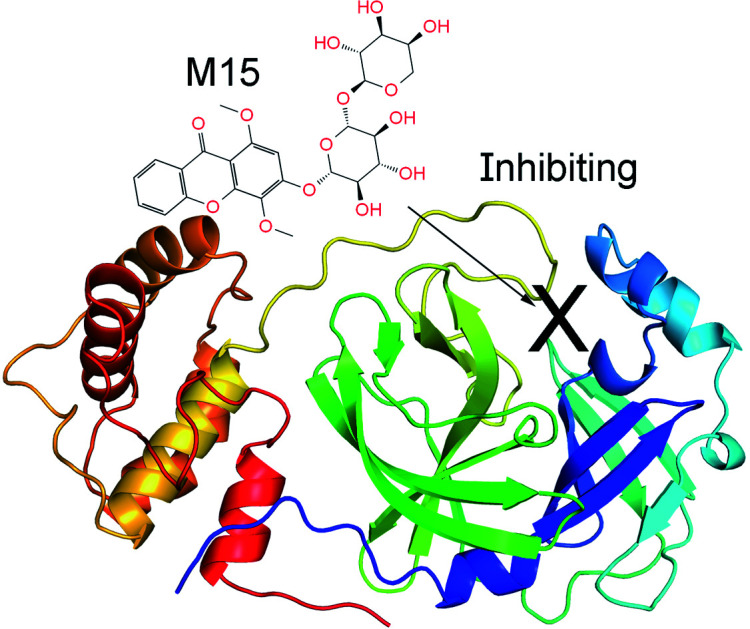

## Introduction

COVID-19 appeared in December 2019 and rapidly spread around the world, becoming the deadliest pandemic in history.^1,2^ As of 18 April 2021, the COVID-19 pandemic has caused more than 140 million infections, with about 3 million deaths, causing economic crisis and upsetting social activities on a global scale.^3^ The causative agent is Severe Acute Respiratory Syndrome Coronavirus 2 (SARS-CoV-2) virus, a novel betacoronavirus of the coronavirus family to infect humans.^4,5^ The coronavirus family was previously known to be the cause of Severe Acute Respiratory Syndrome (SARS) and Middle East Respiratory Syndrome (MERS) outbreaks.^6^ Currently, a few vaccines are approved. Many countries are making efforts to speed up the vaccination process. However, due to the low vaccination rate, the lack of specific treatment methods, as well as the emergence of many new variants, the epidemic continues to spread rapidly and complicatedly.

The papain-like protease (PLpro) and main protease (Mpro) or 3-chymotrypsin-like main protease (3CLpro) are two distinctive cysteine proteases that are essential for the viral replication cycle encoded by the SARS-CoV-2 genome.^7,8^ Mpro exists as a functional homodimer with two active sites, each containing a Cys–His catalytic dyad undertaking hydrolysis of peptide bonds, resulting in cleavage of translated polyproteins into individual segments for the coronavirus to use. The crystalline structure of this protease in the free or bound form of inhibitors has been resolved and stored in the Protein Data Bank (PDB).^9,10^ The Mpro is thus played as a potential drug target for preventing viral replication.^11,12^ PLpro is found in all coronaviruses, with two copies, denoted as PL1pro and PL2pro.^10–15^ PLpro cleaves peptide bonds between non-structural proteins (Nsps), including Nsp1 and Nsp2, Nsp2 and Nsp3, Nsp3 and Nsp4, resulting in the release of three proteins: Nsp1, Nsp2 and Nsp3.^16^ In addition to the proteolytic function, PLpro also exhibits other multiple complex and diverse functions. PLpro plays an essential role in the cleavage and maturation of viral polyproteins, assembly of the replicase–transcriptase complex, and disruption of host responses. The high resolution-structure of PLpro from SARS-CoV-2 has also been recently studied and reported.^17^

Natural products have a long history of being the prime source of compounds for the treatment of a wide spectrum of diseases. It has been estimated that more than 60% of the medicines being provided on the market are derived from or inspired by natural products. It thus offers a great opportunity for the discovery of therapeutic agents with biological activities ranging from antiviral to anticancer.^18^ Marine science was mentioned for the first time in the late 19th century, since then, biotechnology becomes the leading field that provides direction to the study of marine.^19^ Over the past decades, emerging evidences have shown that not only plants and terrestrial organisms but marine also plays a vital source for developing new drugs with greater efficacy and specificity for the therapeutics.^20,21^ Notably, marine fungi and bacteria represent as promising sources with many unique chemical structures have been found from the drug discovery.^22–24^ On the other hand, these sources have the advantage of sustainable production of large quantities of compounds with reasonable costs and large-scale cultivation.

Non-covalent bonding chemical reaction between a small compound and an enzyme target mostly corresponds to the enzymatic inhibition.^25,26^ The Gibbs free energy change during the reaction, Δ*G*, can be assessed in molecular dynamics (MD) simulations.^27,28^ The metric is associated with the inhibition constant, *k*_i_, *via* formula Δ*G* = *RT* ln(*k*_i_), where *T* is the absolute temperature and *R* is the gas constant. It should be noted that half maximal inhibitory concentration, IC_50_, is popularly approximated to be replaced *k*_i_ to predict the experimental ligand-binding affinity, Δ*G*_EXP_.^29–31^ Accurate and precise prediction of Δ*G* thus plays an important role to characterize the best compounds to inhibit the biological activity of an enzyme target. Therefore, several computational investigations were performed to predict potential inhibitors for SARS-CoV-2 Mpro.^31–42^ In this context, molecular docking using AutoDock Vina^43^ and fast pulling of ligand (FPL)^44^ simulations were employed to investigate the ligand-binding affinity of marine fungi compounds to SARS-CoV-2 Mpro. It should be noted that the FPL simulation is an affordable scheme, which adopted a good correlation coefficient to the respective experiment in screening potential inhibitors for SARS-CoV-2 Mpro using low-cost resources.^27^ In this benchmark, the FPL accuracy equals the linear interaction energy approach and better than that of the molecular mechanics Poisson–Boltzmann surface area method. However, FPL scheme requires significantly smaller computing resources.^27^ Obtained results suggested that 11 compounds can become potential candidates for preventing SARS-CoV-2 replication.

## Materials and methods

### Structure of ligands and SARS-CoV-2 Mpro

The high-resolution three-dimensional conformations of SARS-CoV-2 Mpro were downloaded from the Protein Data Bank (PDB) with the PDB code of 7JYC.^45^ The structures of 690 compounds (Table S1 of the ESI†), which were denoted from M1 to M690, were found from marine fungi samples in previous works.^46–49^

### Molecular docking simulations

**Fig. 1:**
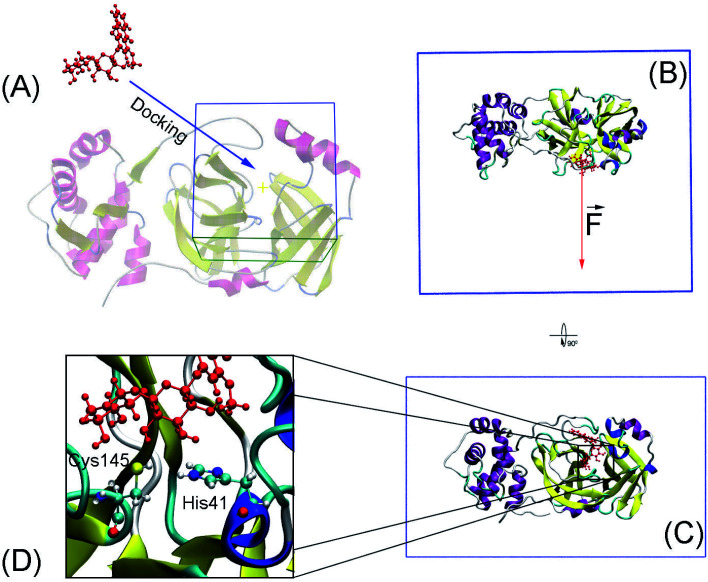
Computational scheme including molecular docking and steered-molecular dynamics simulations. (A) Molecular docking using AutoDock approaches. (B) and (C) FPL initial conformation of SARS-CoV-2 Mpro + M15 in a different perspective. (D) The protonation states of His41 and Cys145.

Molecular docking was performed to dock ligands to the receptor as described in Fig. 1A. The AutoDockTools version 1.5.6 (ref. 50) was employed to generate PDBQT files of the receptor and ligands, which are required for molecular docking simulations.

#### AutoDock Vina (Vina)^43^

Marine compounds were docked to the binding cleft of SARS-CoV-2 Mpro *via* the Autodock Vina (Vina)^43^ with docking parameters obtained from the previous studies.^27,51^ In particular, exhaustiveness was 8, 56, and 400 denoting as short, medium, and long options. The docking grid was of 2.4 × 2.4 × 2.4 nm.^27^ The grid center was the Narlaprevir center of mass.^45^ The lowest binding energy is selected as the docked model.

#### AutoDock4 (AD4)^50^

AD4 was also performed using the docking grid of 72 × 72 × 72 with a spacing of 0.333 Å.^27^ In particular, the docking grid was generated *via* AutoGrid4. The genetic algorithm (GA)/Lamarckian GA (LGA) run was 10. The population size was 150. The number of generations was 27 000. The GA number of evaluations was 250 000, 2 500 000, and 25 000 000 corresponding to short, medium, and long options.^51^ The lowest binding energy cluster was selected as the docked model.

### Molecular dynamics simulations

Conventional atomistic simulations were performed by using GROMACS v5.1.5.^52^ According to the previous works,^27,53^ Amber99SB-iLDN force field^54^ was utilized to mimic protein and counter ions. TIP3P water model^55^ was utilized to represent water molecules. The general Amber force field (GAFF)^56^ was employed to present ligands, in which, AmberTools18 (ref. 57) and ACPYPE^58^ were used to generate ligand topology *via* density functional theory calculation at B3LYP/6-31G(d,p) level of theory.

The solvated SARS-CoV-2 Mpro + inhibitor was put in a water box (*cf.*Fig. 1B and C) with a size of 9.83 × 5.92 × 8.70 nm. The water box was selected referring to the previous work,^59^ in which the complex comprises of *ca.* 50 000 atoms totally. Energy minimization, NVT (100 ps), and NPT (2.0 ns) simulations were carried out to relax the complex in sequence. During NVT and NPT, C_α_ atoms of SARS-CoV-2 Mpro were retrained *via* a harmonic force. The relaxed conformation of complexes was then used as an initial shape for FPL calculations. In particular, the inhibitor was dissociated *via* a harmonic force with a cantilever of a spring constant of 600 kJ mol^−1^ nm^−2^. The dissociate velocity is of 0.005 nm ps^−1^ constantly. In FPL simulations, the ligand-binding affinity was proportionally dependent on the magnitude of the pulling work, because the work is associated with the ligand-binding free energy according to Jarzynski equality.^60,61^ Then, the predicted binding free energy can be calculated as formula Δ*G*^Pre^_FPL_ = −0.056 × *W* − 5.512.^59^ Systemic details were recorded every 0.1 ps for analyzing results.

### Data analysis

Chemicalize,^62^ an online application of ChemAxon, was used to estimate the protonation state of ligand. The computed error was calculated using the bootstrapping scheme.^63^ Receiver Operating Characteristics-Area Under The Curve (ROC-AUC) was calculated using the Scikit-Learn library,^64^ in which the ligands were arranged into two groups including weak and strong binders.

## Results and discussion

SARS-CoV-2 Mpro, a cysteine protease, contains a Cys–His catalytic dyad in the active site.^10^ Several ligands formed a covalent bond to Cys145Sγ in X-ray crystallography such as 6LU7,^65^ 6Y2F,^11^ and 7JYC.^45^ The protonation states of His41 and Cys145 probably alter the calculated ligand-binding affinity that were assigned as shown in Fig. 1D according to the previous work.^27^

Thermodynamic metric, Δ*G* – binding free energy, which characterized the protein–ligand association, was initially estimated using molecular docking simulations. In this context, we firstly assessed the performance of both AutoDock 4 (AD4)^50^ and AutoDock Vina (Vina)^43^ since they are one of the most common open-source docking applications.^51^ In particular, AD4 implements various docking algorithms, involving GA and LGA, the performance of these algorithms was thus evaluated. It should be emphasized that AD4 employs a physical-based plus empirical scoring function, while Vina is purely an empirical scoring function docking approach.^43,50^ Therefore, the performance of these docking approaches would be expectedly different from each other. Although the AD4/Vina accuracies accounting for SARS-CoV-2 Mpro target were recently evaluated, the benchmark was completed using the short option only.^27^ In this context, a deeper assessment was carried out including the accuracy depending on the docking options and algorithms. The obtained docking results were described in Table 1 in comparison with the respective experiments.

**Table 1:** The obtained values of the docking simulations

No.	Compound	Δ*G*^LGA^_AD4_			Δ*G*^GA^_AD4_			Δ*G*_Vina_			Δ*G*_EXP_^a^
		Short	Medium	Long	Short	Medium	Long	Short	Medium	Long	
1	Atazanavir	−7.61	−10.46	−14.41	−4.55	−9.50	−8.48	−8.30	−8.20	−8.20	−8.64
2	Candesartan cilexetil	−8.22	−11.19	−11.56	−7.90	−10.88	−11.22	−8.10	−8.10	−8.10	−9.23
3	Chloroquine	−7.41	−8.11	−8.35	−6.79	−7.46	−7.78	−6.10	−6.10	−6.10	−8.56
4	Cimetidine	−6.08	−6.97	−7.32	−5.6	−6.00	−6.14	−6.10	−5.80	−6.10	−7.51
5	Maribavir	−8.59	−10.36	−10.43	−6.82	−7.42	−7.7	−6.60	−6.60	−6.60	−7.51
6	Omeprazole	−8.02	−8.28	−8.54	−7.06	−7.91	−8.09	−7.70	−7.80	−7.70	−8.03
7	Oxytetracycline	−10.06	−10.38	−10.85	−8.77	−8.56	−8.58	−8.20	−8.20	−8.30	−8.22
8	Roxatidine acetate hydrochloride	−7.02	−8.11	−9.47	−5.74	−6.48	−7.13	−7.10	−7.10	−7.10	−8.05
9	Sulfacetamide	−5.53	−5.95	−5.96	−5.28	−5.88	−5.89	−5.80	−5.80	−5.80	−7.51
10	Valacyclovir hydrochloride	−6.08	−8.18	−9.66	−5.58	−5.03	−5.35	−6.50	−6.60	−6.40	−8.16

a The experimental binding free energy was obtained through inhibition constant *k*_i_.^66^ The units of energy and force are in kcal mol^−1^ and pN, respectively.

**Fig. 2:**
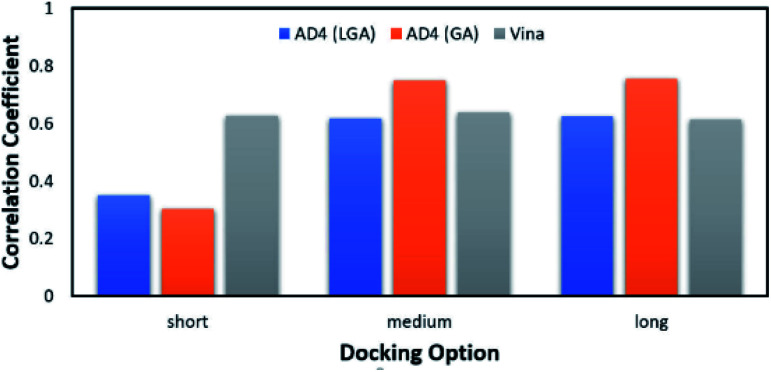
The Pearson correlation coefficient between docking and experimental data. The docking results were obtained using AD4 and Vina in various docking options.

According to the previous study,^51^ Vina accuracy is less dependent on the docking options since the package rapidly converges, but AD4 performs a different way that AD4 accuracy is strongly dependent upon the selected docking options. In particular, in good agreement with the recent work,^27^ AD4 using two algorithms formed uncorrelated results when the docking option was short. However, AD4 accuracy rapidly increased when the GA number of evaluations was enlarged (Fig. 2). Especially, when LGA algorithm was induced, the Pearson correlation coefficient, *R*, between docked and experimental data adopted as 0.35, 0.62, and 0.62 corresponding to docking options short, medium, and long, respectively. Besides, when GA algorithm was induced, the metric *R* was 0.32, 0.75, and 0.76 responding to short, medium, and long options, respectively. However, the corresponding metric of Vina approach is almost unchanged during the alteration of the docking option, in which the *R*_Vina_ is 0.63, 0.64, and 0.61, respectively.

**Fig. 3:**
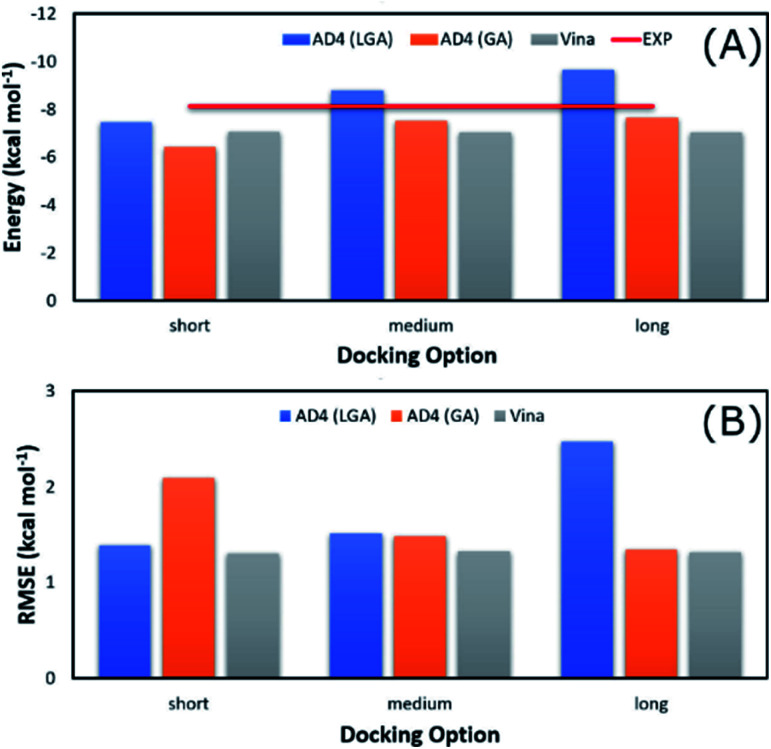
Comparison between the mean of binding free energy providing by docking and experimental approaches. The docking results were obtained by AD4 and Vina *via* various options.

The average of binding free energy, Δ*G*, provided by AD4 was significantly decreased upon changing the docking options (*cf.*Fig. 3A). The AD4 package using GA algorithm and long option formed the smallest difference of Δ*G* in comparison with the respective experiments. It should be emphasized that the experimental Δ*G* is of −8.14 ± 0.17 kcal mol^−1^ denoting by the red horizontal line in Fig. 3A. Moreover, Vina package adopted an unchanged difference value Δ*G*, *ca.* 1.10 kcal mol^−1^, compared with the experiments. Moreover, the RMSE analysis indicated that Vina approach is more accurate than AD4 (*cf.*Fig. 3B). In particular, Vina formed a mean RMSE of 1.32 kcal mol^−1^, which is significantly smaller than that by AD4 with 1.79 and 1.60 kcal mol^−1^, when AD4 uses LGA and GA algorithms, respectively. However, AD4 used GA algorithm and the long option adopted an RMSE of 1.34 kcal mol^−1^, which is no different from Vina approach (Fig. 3B).

AD4 with GA algorithm using the long option is the best solution if we only cared about the accurate issue. However, AD4 with these options consumed more than one hour (*ca.* 74 minutes) to dock a ligand to SARS-CoV-2 Mpro. It is a huge value in comparison with Vina approach, which solitary requires a few minutes (*ca.* 2 minutes only) to complete suck the same task. The CPU time consumption is much more important when a large number of ligands would be investigated. Therefore, although AD4 is more accurate, Vina with the short option is employed to preliminarily estimate the binding affinity and pose of marine fungi compounds to SARS-CoV-2 Mpro.

**Fig. 4:**
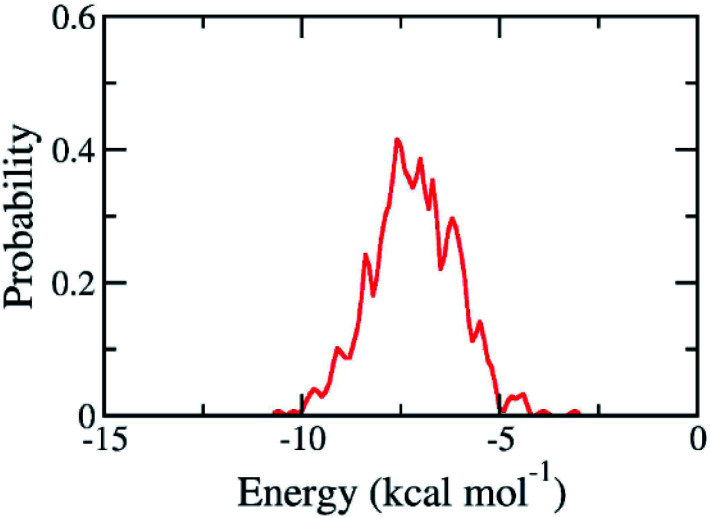
Contribution of docking energy of marine fungi compounds targeting SARS-CoV-2 Mpro.

The binding pose and affinity of marine fungi compounds to SARS-CoV-2 Mpro were thus performed using Vina package. The binding free energy between these compounds to SARS-CoV-2 Mpro falls in the range from −3.1 to −10.6 kcal mol^−1^, whose distribution was shown in Fig. 4. The mean value of docking energy is −7.17 ± 0.04 kcal mol^−1^. We have selected 16 top-lead compounds, which are equivalent to 2% of total compounds, as candidates for further analysis *via* FPL calculations.

**Fig. 5:**
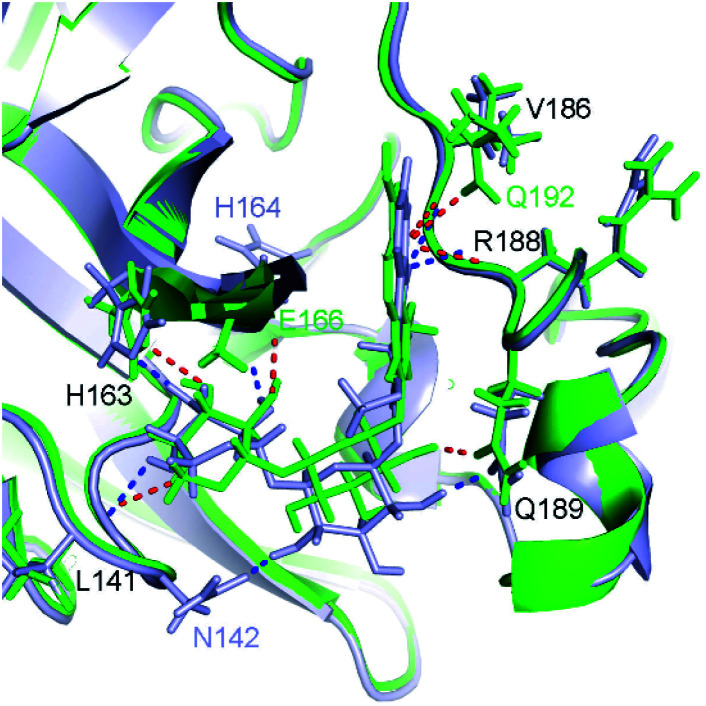
Comparison between docked (green) and MD-refined (gray) structures of SARS-CoV-2 Mpro + M15. Black texts represent residues, which form HBs to both docked and MD-refined structures. Gray texts represent residues, which only form HBs to MD-refined structure. Green texts mention residues, which only form HBs to the docked ligand.

Atomistic simulations were often used to validate the obtained results *via* molecular docking approaches because numerous approximations were implemented during docking simulations.^27,43,50^ In this context, as mentioned above, the Vina docking poses were used to generate initial structures of FPL simulations.^27^ It should be noted that FPL simulation was indicated that it is a highly appropriate scheme to validate docking results for SARS-CoV-2 Mpro system.^27,59^ Although FPL accuracy is smaller than that by the free energy perturbation (FEP) method but it is extremely required a small number of computing resources compared with FEP one.^27^ Moreover, different from the end-to-end free energy approach, FPL probably provides information about unbinding process of ligands out of the enzyme cavity. During FPL simulations, the predicted ligand-binding free energy, Δ*G*^Pre^_FPL_, was thus obtained according to the formula Δ*G*^Pre^_FPL_ = −0.056 × *W* − 5.512, where *W* is the pulling work.^59^ The predicted IC^Pre^_50_ was thus calculated *via* formula 
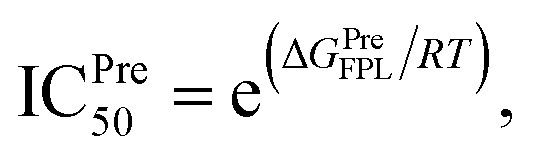
 where *T* = 310 K is the absolute temperature and *R* = 1.982 cal mol^−1^ K^−1^ is gas constant, with an approximation that IC_50_ equals to inhibition constant *k*_i_. Moreover, because applying numerous approximations to accelerate computing speed, the difference between docking pose and native binding pose is available since the success docking rate only is 67%.^27^ Therefore, the ligand-binding pose was normally refined using MD simulation.^27^ Because the MD-refined structure of the complex was slightly changed in comparison with the docking pose, the interaction diagram was also altered (Fig. 5 and Table S2 of ESI†).

**Fig. 6:**
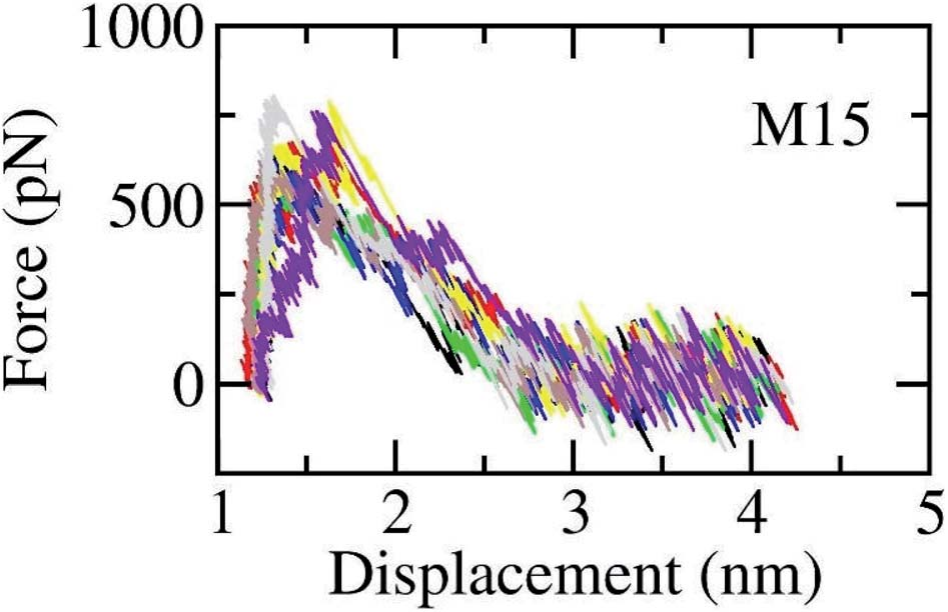
The pulling force in displacement dependence over FPL simulations. The results were obtained *via* 8 independent FPL trajectories.

The SARS-CoV-2 Mpro + inhibitor complexes were inserted into the solvation, which included water and counterions (*cf.*Fig. 1), in which the complexes were rotated to align the unbinding direction to *Z*-axis. The unbinding pathway was selected according to the previous work.^31^ The solvated system was then relaxed during conventional MD simulations as mentioned in Materials and methods section. There are 8 independent SMD trajectories that were produced to estimate the unbinding process of ligands out of SARS-CoV-2 Mpro active site. During FPL simulations, the pulling force profile is probably divided into three regions. In particular, the pulling force rapidly rises to the maximum value, *F*_Max_, upon the first region; the metric *F* is then decreased to zero value in the second region; and the value fluctuates around zero value in the third region (*cf.*Fig. 6 and Table S3 of the ESI†). It may argue that there is two transition state (TS) points during the ligand dissociate process. The first TS corresponds to where the pulling work reaches the value *F*_Max_ meaning that the ligand is starting to dissociate from the binding cavity of the enzyme. The second TS corresponds to the point that the non-bonded contact between protein–ligand is terminated. The difference between the displacement of two TSs approximately is 0.9 nm responding to the non-covalently contact radius.

Although, as mentioned above, the FPL simulations were indicated that it is an appropriate approach for estimating the ligand-binding affinity of SARS-CoV-2 Mpro,^27,59^ the validated simulations were also carried out. An appropriate correlation coefficient between predicted binding free energy and the respective experimental data was obtained (*R* = −063 ± 0.21). Details of results were reported in Table S4 of the ESI.† Although the correlation coefficient of FPL is not enhanced in comparison with Vina (*R* = 0.62), the ROC-AUC results indicated that FPL, ROC-AUC = 0.80 ± 0.16, is better than Vina, ROC-AUC = 0.74 ± 0.18, in order to classify the inhibitors.

**Fig. 7:**
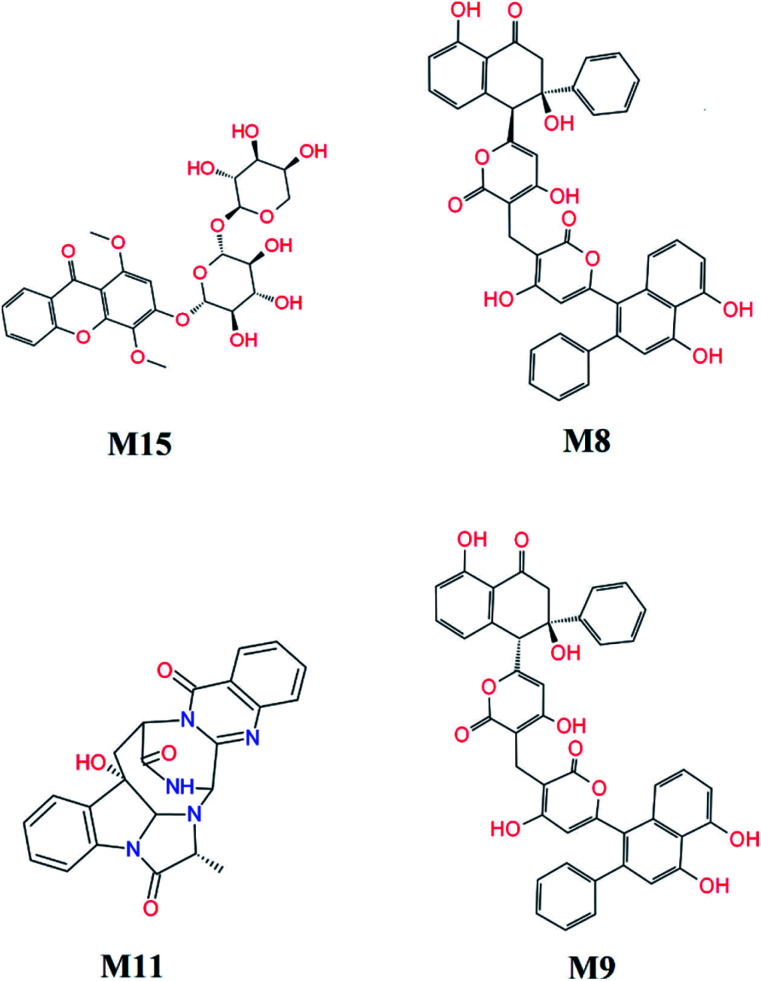
Highly potent inhibitors for SARS-CoV-2 Mpro estimated by molecular docking and FPL simulations from marine fungi compounds. The ADME estimation was reported in Table S5 of the ESI,† in which all properties are appropriate.

FPL calculations were then applied to refine the docking results. The binding free energy of 16 top-lead compounds was predicted using FPL simulations. The acquired results were shown in Table 2. In particular, four compounds including M15, M8, M11, and M9 (*cf.*Fig. 7) were indicated that they are highly potent inhibitors with the predicted value IC^Pre^_50_ in the range of high-nanomolar. Eight compounds were followed with the value IC^Pre^_50_ is in the range of sub-micromolar. Four compounds are able to bind to SARS-CoV-2 Mpro with IC^Pre^_50_ in the range of micromolar affinity. Absolutely, eleven compounds were suggested that they are highly potent inhibitors for COVID-19 treatment. However, further experimental work should be performed to validate the observation.

**Table 2:** The computational values using molecular docking and FPL simulations

No.	Compound	Δ*G*_Dock_	*F*_Max_	*W*	Δ*G*^Pre^_FPL_^a^	Predicted IC^Pre^_50_ range^b^
1	M15	−9.4	692.8 ± 26.7	77.7 ± 3.7	−9.87	High-nanomolar
2	M8	−9.7	636.4 ± 37.9	77.0 ± 3.1	−9.82	High-nanomolar
3	M11	−9.6	764.9 ± 32.0	73.4 ± 3.6	−9.62	High-nanomolar
4	M9	−9.7	601.5 ± 30.2	68.5 ± 2.7	−9.35	High-nanomolar
5	M2	−10.2	579.2 ± 49.4	60.5 ± 4.6	−8.90	Sub-micromolar
6	M13	−9.5	587.9 ± 37.6	59.4 ± 5.3	−8.84	Sub-micromolar
7	M3	−9.9	593.1 ± 35.3	59.0 ± 2.5	−8.82	Sub-micromolar
8	M5	−9.8	595.8 ± 30.1	58.4 ± 2.1	−8.78	Sub-micromolar
9	M4	−9.9	549.1 ± 32.3	56.6 ± 3.9	−8.68	Sub-micromolar
10	M16	−9.4	587.5 ± 37.4	54.6 ± 3.8	−8.57	Sub-micromolar
11	M6	−9.8	551.1 ± 24.7	54.0 ± 3.0	−8.54	Sub-micromolar
12	M1	−10.6	530.2 ± 20.0	50.8 ± 2.4	−8.36	Micromolar
13	M12	−9.6	512.9 ± 28.0	44.1 ± 1.3	−7.98	Micromolar
14	M14	−9.4	447.9 ± 19.0	40.7 ± 2.5	−7.79	Micromolar
15	M7	−9.7	418.8 ± 30.2	37.7 ± 2.9	−7.62	Micromolar
16	M10	−9.6	428.9 ± 20.3	34.3 ± 1.8	−7.43	Micromolar

a The predicted binding affinity Δ*G*^Pre^_FPL_ = −0.056 × *W* − 5.512.^59^

b The predicted IC^Pre^_50_ was calculated *via* formula 
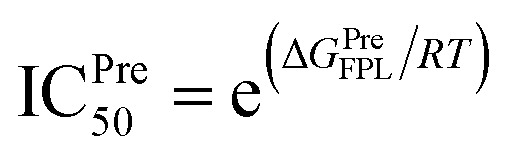
 with assumption that IC_50_ equals to inhibition constant *k*_i_. The computed error is the standard error of the mean. The unit of force and energy in pN and kcal mol^−1^, respectively.

**Fig. 8:**
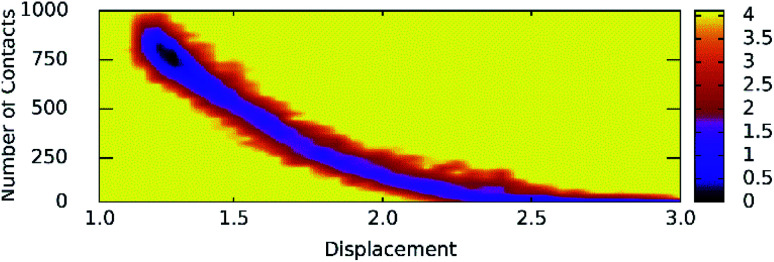
The collective-variable FEL exposing the unbinding pathway of M15 out of SARS-CoV-2 Mpro cavity.

Unbinding pathways of ligands from SARS-CoV-2 Mpro binding cavity can be characterized over FPL simulations. Dissociate process of compound M15 was repeated 50 independent times, which is in good agreement with 8 trajectories reported above with *F*_Max_ = 688.7 ± 12.2 pN and *W* = 77.6 ± 1.6 kcal mol^−1^. In particular, the coordinates of the complex were recorded every 10 ps to construct the collective-variable free energy landscape (FEL). 2D FEL was built using the displacement of the ligand and the number of contacts between protein–ligand in the range of 0.45 nm. The constructed FEL was shown in Fig. 8. In consistent with Fig. 6, the number of contacts between M15 and SARS-CoV-2 Mpro quickly reduced when the inhibitor displacement varies from 1.1 to 2.1 nm. During the unbinding process, one minimum was only observed, which corresponded to the bound state of M15 to the Mpro. Unfortunately, no minimum was found over unbinding path due to continuously affecting the pulling force on the ligand. It may argue that there is a limitation of information about unbinding pathway provided over FPL simulations because a large pulling speed was applied. A new tool should be developed for removing biased information of FPL calculation as well as umbrella sampling simulations^67^ or a significantly slow-pulling speed should be applied to be able to obtain the unbinding pathway information. Further work should be carried out to clarify the issue.

## Conclusions

We have demonstrated that Vina is better than AD4 in order to screen the ligand-binding affinity of a large database of compounds to SARS-CoV-2 Mpro, because Vina forms appropriate results using a small computing resource. ROC-AUX analysis suggested that FPL was better than Vina in classifying the inhibitors. FPL simulations were also provided physical insight into unbinding pathway of ligands from SARS-CoV-2 Mpro cavity.

Eleven marine fungi compounds adopted a large ligand-binding affinity to SARS-CoV-2 Mpro including four compounds in the range of high-nanomolar affinity and eight in the range of sub-micromolar affinity predicted IC^Pre^_50_. In particular, four compounds M15, M8, M11, and M9 formed the predicted ligand-binding free energy of −9.87, −9.82, −9.62, and −9.35 kcal mol^−1^, whereas the others adopted predicted ligand-binding free energies in the range from −8.54 to −8.94 kcal mol^−1^. All of these compounds probably play as potential inhibitors for preventing SARS-CoV-2 Mpro, however, further study should be carried out to confirm the suggestion.

In addition, unbinding pathway of ligands from SARS-CoV-2 Mpro + inhibitor complexes would not be clarified *via* FPL simulations, because a high pulling speed was applied. Further work should be carried out to clarify the issue. For example, a new method should be developed for avoiding the biased information generated by FPL calculation or studying the dissociate problem using significantly slow-pulling velocity.

## Conflicts of interest

There are no conflicts to declare.

## Supplementary Material

RA-011-D1RA03852D-s001
