# Supplementary material for: Potential inhibitors for SARS-CoV-2 Mpro from marine compounds
Source: RSC Adv. 2021 Jun 23;11(36):22206–13. doi: 10.1039/d1ra03852d (PMC9034196; doi:10.1039/d1ra03852d)
Supplement: RA-011-D1RA03852D-s001 [file RA-011-D1RA03852D-s001.pdf]

Electronic Supplementary Material (ESI) for RSC Advances.
This journal is © The Royal Society of Chemistry 2021

Supporting Information

Potential inhibitors for SARS-CoV-2 Mpro from Marine compounds

Nguyen Minh Tam,[ab+] Minh Quan Pham,[cd+] Huy Truong Nguyen,[e] Nam Dao Hong,[f] Nguyen Khoa Hien,[dg] Duong Tuan Quang,[h] Huong Thi Thu Phung,[i] and Son Tung Ngo[b,j]*

[a.] Computational Chemistry Research Group, Ton Duc Thang University, Ho Chi Minh City, Vietnam
[b.] Faculty of Applied Sciences, Ton Duc Thang University, Ho Chi Minh City, Vietnam
[c.] Graduate University of Science and Technology, Vietnam Academy of Science and Technology, Hanoi, Vietnam
[d.] Institute of Natural Products Chemistry, Vietnam Academy of Science and Technology, Hanoi, Vietnam
[e.] Faculty of Pharmacy, Ton Duc Thang University, Ho Chi Minh City, Vietnam
[f.] University of Medicine and Pharmacy at Ho Chi Minh City, Ho Chi Minh City, Vietnam
[g.] Mientrung Institute for Scientific Research, Vietnam Academy of Science and Technology, Hue City, Thua Thien Hue Province, Vietnam
[h.] Department of Chemistry, Hue University, Hue City, Thua Thien Hue Province, Vietnam
[i.] NTT Hi-Tech Institute, Nguyen Tat Thanh University, Ho Chi Minh City, Viet Nam
[j.] Laboratory of Theoretical and Computational Biophysics, Ton Duc Thang University, Ho Chi Minh City, Vietnam.
[+] Authors equally contribute to the work.
*Email: ngosontung@tdtu.edu.vn

Table S1. List of marine compounds and docking energy to SARS-CoV-2 Mpro.

N^0	Name	SMILES	ΔG_{Dock}	Marine source
1	**M1**	Cc1cc2c(c(c1)O)C(=O)c1c(ccc(c3c(C)cc(c4c3C(=O)c3cccc(c3C4=O)O)O)c1O)C2=O	-10.6	*Laurenica spectabilis*
2	**M2**	C1(=C)C[C@@H]2[C@@H]3[C@]4([C@@H]1C[C@H]4C(O[C@@H]1[C@H]4[C@@H]3[C@@H](C[C@@H]2Cl)[NH2+][C@H]4[C@]2([C@H]1CC[C@@]1([C@]2(C)CC[C@H]2[C@@]31[C@@H]([C@H]([C@H](O2)C(=C)C)O)O3)O)C)(C)C)O	-10.2	*Mucor irregularis*
3	**M3**	c1ccc2c(c1)N=C([N+](C2=O)[C@H]1C[C@]2(OC1=O)c1c(N3[C@H]2NC2(C3=O)CC2)cccc1)C(=O)N	-9.9	*Aspergillus versicolor* LZD-14-1
4	**M4**	C1(=C)C[C@@H]2[C@@H]3[C@H]4[C@@H]1C[C@H]4C(O[C@@H]1[C@H]4[C@@H]3[C@@H](C[C@@H]2Cl)[NH2+][C@H]4[C@]2([C@H]1CC[C@@]1([C@]2(C)CC[C@H]2[C@@]31[C@@H]([C@H]([C@H](O2)C(=C)C)O)O3)O)C)(C)C	-9.9	*Mucor irregularis*
5	**M5**	c1ccc2c(c1)N=C1[N+](C2=O)[C@@H]2C(=O)N[C@@H]1N1[C@@H]3[C@](C2)(c2c(N3C(=O)C31CC3)cccc2)O	-9.8	*Aspergillus versicolor* LCJ-5-4
6	**M6**	[C@H]12N3C(=O)[C@@H](N(C(=O)[C@@H]3C[C@]1(c1ccccc1N2)[C@]12[C@H](N3C(=O)[C@@H](N(C(=O)[C@@H]3C1)C)Cc1ccccc1)Nc1c2cccc1)C)Cc1ccccc1	-9.8	Mangrove endophytic fungus Gx-3a
7	**M7**	c1ccc2c(c1)N=C1[N+](C2=O)[C@@H]2C(=O)N[C@H]1O[C@@]1([C@@H]3NC4(C(=O)N3c3c1cccc3)CC4)C2	-9.7	*Aspergillus versicolor* LZD-14-1
8	**M8**	c1(c(cc(c2c1cccc2O)O)c1ccccc1)C1=CC(=C(C(=O)[OH+]1)CC1=C(C=C([OH+]C1=O)[C@@H]1[C@](CC(=O)c2c1cccc2O)(c1ccccc1)O)O)O	-9.7	*Streptomyces* sp.
9	**M9**	c1(c(cc(c2c1cccc2O)O)c1ccccc1)C1=CC(=C(C(=O)[OH+]1)CC1=C(C=C([OH+]C1=O)[C@H]1[C@](CC(=O)c2c1cccc2O)(c1ccccc1)O)O)O	-9.7	*Streptomyces* sp.
10	**M10**	c1ccc2c(c1)N=C1[N+](C2=O)[C@@H]2C(=O)N3[C@H]1O[C@@]1([C@@H]4N(C3)[C@H](C(=O)N4c3c1cccc3)C)C2	-9.6	*Aspergillus versicolor* LZD-14-1
11	**M11**	c1ccc2c(c1)N=C1[N+](C2=O)[C@@H]2C(=O)N[C@@H]1N1[C@@H]3[C@](C2)(c2c(N3C(=O)[C@H]1C)cccc2)O	-9.6	*Aspergillus versicolor* LCJ-5-4
12	**M12**	C1CC[C@H]2[C@@H](C1)[NH2+]C[C@H]2[C@@]12[C@@H]3[C@H](CCCC3)N[C@@H]1N1[C@]3([C@H]2O)C(=O)N([C@@](C1=O)(C)SSS3)C	-9.6	*Acrostalagmus luteoalbus*
13	**M13**	[C@]12([C@H]3C(=C)C[C@@H]4[C@@H]1[C@H]1[C@@H](C[C@H]4Cl)[NH2+][C@@H]4[C@H]1[C@@H](OC([C@H]2C3)(C)C)O[C@]1([C@]4([C@@]2([C@@](CC1)([C@]13[C@H](CC2)O[C@@H]([C@@H]([C@H]1O3)O)C(=C)C)O)C)C)OC)O	-9.5	*Mucor irregularis* QEN-189
14	**M14**	c1ccc2c(c1)[nH+]c1c2cc([nH]c1C(=O)C)C(=O)NCC[C@H]1[C@H]2[C@H]([NH2+]C1)CCCC2	-9.4	*Marinactinospora thermotolerans* SCSIO 00652

15	**M15**	C1[C@@H]([C@@H]([C@H]([C@@H](O1)O[C@@H]1[C@H]([C@@H]([C@H]([C@@H](O1)Oc1c(c2c(c(c1)OC)C(=O)c1c([OH+]2)cccc1)OC)O)O)O)O)O)O	-9.4	*Phomopsis* sp. ZH76
16	**M16**	[C@@]123OC(=O)CC/C=C(/C(=O)[C@H](C/C=C/[C@H]1[C@@H](C(=C)[C@H]([C@H]2[C@@H](NC3=O)Cc1ccccc1)C)O)C)\C	-9.4	*Arthrinium arundinis* ZSDS1-F3
17	**M17**	O1[C@@]2([C@@H]3N(c4c2cccc4)C(=O)[C@@H](N3)C)C[C@H]2[N+]3C(=Nc4c(C3=O)cccc4)[C@]1(NC2=O)C	-9.3	*Aspergillus* sp.
18	**M18**	c12c(cc(cc1[OH+]c1c(C2=O)c(cc(c1)OC)[C@H]1[C@@H](c2c(C1=O)cc(cc2O)OC)c1c(cc(cc1O)C)O)C)O	-9.3	*Ascomycota* sp. SK2YWS-L
19	**M19**	O=C1c2cc(OC)cc(O)c2[C@H](c2c(O)cc(C)cc2O)[C@H]1c1cc(OC)cc2c1C(=O)c1c([OH+]2)cc(C)cc1O	-9.3	*Ascomycota* sp. SK2YWS-L
20	**M20**	c1cc(ccc1)C[C@@H]1NC(=O)CN(C(=O)[C@H]2N(C(=O)[C@H](N(C(=O)[C@@H](NC(=O)[C@@H](NC(=O)[C@@H](N(C1=O)C)Cc1ccc(cc1)O)C(C)C)CC(C)C)C)Cc1ccc(cc1)O)CCC2)C	-9.2	*Acremonium persicinum* SCSIO115
21	**M21**	C1=C[C@H]2[C@@H](OC1(C)C)C[C@@H]1[C@H](C2)[C@@H]([C@H]([NH2+]1)C(C)(C=C)C)/C=C\1/C(=O)N2[C@@H](C(=O)N1)CCC2	-9.2	*Aspergillus versicolor*
22	**M22**	C1C[C@H]2[C@@H](CC1)[C@H](C[NH2+]2)C[C@@H]1[C@H]2[C@@]3(C(=O)N1)[C@H]([C@@H]([C@@]([C@H]2C)(C)O)C)/C=C/C[C@H](C)/C=C(/C(=O)[C@@H](O)CCC3=O)\C	-9.2	*Chaetomium globosum*
23	**M23**	O=c1c2ccccc2ncn1[C@@H]1C[C@]2([C@H]3N(C1=O)[C@H](C(=O)N3c1c2cccc1)C)O	-9.2	*Penicillium* sp.
24	**M24**	C1C[C@@H]2[C@@H](CC1)[C@@H](C[NH2+]2)C[C@@H]1[C@H]2[C@@]3(C(=O)N1)[C@H]([C@@H](C(=C2C)C)O)/C=C/C[C@H](C)/C=C(/C(=O)[C@@H](O)CCC3=O)\C	-9.1	*Chaetomium globosum*
25	**M25**	O1[C@H]([C@H]([C@H](C[C@@H]1O[C@@H]1C[C@@](O[C@@H]([C@H]1CC)C)([C@H]([C@@H]([C@@H]([C@@H]1[C@H](/C=C/C=C/C(=C)O[C@@H]([C@H](/C=C/C=C/C(=O)O1)C)[C@H]([C@H]([C@@H](C1=C[C@H]([C@@H]([C@H](O1)C)CC)O[C@H]1C[C@@H]([C@@H]([C@@H](O1)C)O)O)C)O)C)C)C)O)C)O)O)O)C	-9.1	*Streptomyces* sp.
26	**M26**	c1cccc(c1O)C1=N[C@@H](CO1)[C@@H]1SC[C@@H](N1C)CN1[C@@H]2C(=O)N([C@H](C1=O)CSSC2)C[C@@H]1N([C@@H](SC1)[C@H]1N=C(OC1)c1c(O)cccc1)C	-9.1	*Streptomyces olivaceus*
27	**M27**	[C@]123[C@@H]4CC[C@@H]([C@]4(CC[C@@H]1[C@]1(CC[C@@H](C[C@]1(OO2)C=C3)O)C)C)[C@H](C)/C=C/[C@@H](C(C)C)C	-9.1	*Paecilomyces lilacinus* ZBY-1
28	**M28**	C1CC[C@H]2[C@@H](C1)[NH2+]C[C@H]2[C@@]12[C@@H]3[C@H](CCCC3)N[C@@H]1N1[C@]3([C@H]2O)C(=O)N([C@@](C1=O)(CO)SSS3)C	-9.1	*Acrostalagmus luteoalbus* SCSIO F457
29	**M29**	C/C=C/[C@@H]1[C@H]2C[C@H](CC[C@@H]2C(=C[C@H]1C(=O)C1=C(C(=C[NH+]C1=O)c1ccc(cc1)O)O)C)C	-9.1	*Campylocarpon* sp. HDN13-307

30	**M30**	[C@]12([C@H]3C(=C)C[C@H]4[C@@H]1[C@@H]1[C@@H](C[C@H]4Cl)[NH2+][C@@H]4[C@H]1[C@@H](OC([C@H]2C3)(C)C)O[C@]1([C@]4([C@@]2([C@@](CC1)([C@]13[C@H](CC2)O[C@@H]([C@@H]([C@H]1O3)O)C(=C)C)O)C)C)O)O	-9.1	*Mucor irregularis* QEN-189
31	**M31**	C1(=C)C[C@@H]2[C@@H]3[C@H]4[C@@H]1C[C@H]4C(O[C@@H]1[C@H]4[C@@H]3[C@@H](C[C@@H]2Cl)[NH2+][C@H]4[C@]2([C@H]1CC[C@@]1([C@]2(C)CC[C@H]2C1=C([C@H]([C@H](O2)C(=C)C)O)O)O)C)(C)C	-9.1	*Mucor irregularis* QEN-189
32	**M32**	[C@@H]1([C@H](C[C@@H]2[C@H](C1)[C@@H](C[NH2+]2)[C@@H]1[C@H](C[NH2+]C1)[C@H]1[C@@H]2[C@@H]([NH2+]C1)C[C@@H]([C@@H](C2)Cl)Cl)Cl)Cl	-9.1	*Marine actinomycete* NPS12745
33	**M33**	c1cc(ccc1)C[C@@H]1NC(=O)CN(C(=O)[C@H]2N(C(=O)[C@H](N(C(=O)[C@@H](NC(=O)[C@@H](NC(=O)[C@@H](N(C1=O)C)Cc1ccc(cc1)O)[C@H](C)CC)CC(C)C)C)Cc1ccc(cc1)O)CCC2)C	-9	*Acremonium persicinum* SCSIO115
34	**M34**	c1ccc2c(c1)NC(=O)[C@H]2[C@@H]1C(=O)Nc2c1cccc2	-9	*Shewanella piezotolerans*
35	**M35**	c1ccc2c(c1)N=C1[N+](C2=O)[C@@H]2C(=O)N[C@@H]1N1[C@@H]3[C@](C2)(c2c(N3C(=O)[C@@H]1C[C@H](CC)C)cccc2)O	-9	*Aspergillus versicolor* LCJ-5-4
36	**M36**	[C@]123[C@@H]4CC[C@@H]([C@]4(CC=C1[C@]1(CC[C@@H](C[C@]1(OO2)C=C3)O)C)C)[C@H](C)/C=C/[C@H](C(C)C)C	-9	*Paecilomyces lilacinus* ZBY-1
37	**M37**	C1CC[C@H]2[C@@H](C1)[NH2+]C[C@H]2[C@@]12[C@@H]3[C@H](CCCC3)N[C@@H]1N1[C@]3(C2)C(=O)N([C@@](C1=O)(CO)SSS3)C	-9	*Acrostalagmus luteoalbus* SCSIO F457
38	**M38**	C1(=O)[C@H]2N(C(=O)[C@H](NC(=O)[C@H]([C@H](OC(=O)[C@@H](N(C)C(=O)CN1C)C(C)C)C)NC(=O)c1ccc(c3c1Nc1c(O3)c(c3c(c1C(=O)N[C@H]1[C@@H](C)OC(=O)[C@H](C(C)C)N(C(=O)CN(C)C(=O)[C@@H]4CCCN4C(=O)[C@@H](C(C)C)NC1=O)C)nc(o3)C)C)C)C(C)C)CCC2	-8.9	*Streptomyces* sp. IMB094
39	**M39**	c1ccc2c(c1)N=C1[N+](C2=O)[C@@H]2C[C@H]([C@]3(O[C@H]1NC2=O)[C@H]1N(c2c3cccc2)C(=O)[C@@H](N1)[C@@H](C)CC)O	-8.9	*Aspergillus versicolor* LZD-14-1
40	**M40**	C1=C[C@H]2[C@H](OC1(C)C)C[C@@H]1[C@@H](C2)[C@H]2C[C@]34[C@H](C[C@]5(N(C3=O)CCC5)C(=O)N4)C([C@@H]2[NH2+]1)(C)C	-8.9	*Aspergillus versicolor*
41	**M41**	[C@@H]12CC[C@]3([C@H]([C@]1(CC[C@@H](C2)C(C)(C)O)C)C(=O)C1=C(O3)C(=O)C(=CC1=O)[C@@H](C(=O)/C(=C/C)/C)C)C	-8.9	*Nigrospora* sp. MA75
42	**M42**	C1[C@H]2[C@@](O[C@@]31c1ccccc1[C@@H]1[C@@H]3CC(C1=C)(C)C)(C(C)(C(=O)C1=Nc3ccccc3C(=O)[N+]21)C)O	-8.9	*Neosartorya Fischeri*
43	**M43**	N1=C2[N+](C(=O)c3ccccc13)[C@@H](C(=O)N[C@H]2C(C)C)C[C@H]1C[NH2+][C@@H]2[C@H]1CCCC2	-8.9	*Neosartorya Fischeri*
44	**M44**	C1CC[C@H]2[C@@H](C1)[NH2+]C[C@H]2[C@@]12[C@@H]3[C@H](CCCC3)N[C@@H]1N1[C@]3([C@H]2O)C(=O)N([C@@](C1=O)(C)SS3)C	-8.9	*Acrostalagmus luteoalbus* SCSIO F457
45	**M45**	c12c([NH+]C(=O)[N+](C1=O)[C@@H]1c3ccccc3NC1=O)cccc2	-8.8	*Shewanella piezotolerans*

46	**M46**	c1ccc2c(c1)N=C1[N+](C2=O)[C@H]2C[C@]3([C@@H]4N([C@@H]1NC2=O)[C@@H](C(=O)N4c1c3cccc1)C)O	-8.8	*Aspergillus versicolor* LZD-14-1
47	**M47**	C1(=O)OC(C2=C[C@@H]3[C@]4([C@H]([C@@]5(C2=C1)CO5)C[C@@]1([C@@H]2C4=C(C(O[C@@H]2OC1=O)(C)C)O3)C)C)(C)C	-8.8	*Talaromyces amestolkiae* YX1
48	**M48**	C1[C@H]2[C@@](O[C@]31c1ccccc1CC3=O)(C(C)(C(=O)C1=Nc3ccccc3C(=O)[N+]21)C)O	-8.8	*Neosartorya fischeri*
49	**M49**	C1CC[C@H]2[C@@H](C1)[NH2+]C[C@H]2[C@@]12[C@@H]3[C@@H](CCCC3)N[C@@H]1N1[C@]3(C2)C(=O)N([C@@](C1=O)(CO)SS3)C	-8.8	*Acrostalagmus luteoalbus* SCSIO F457
50	**M50**	O=C1N2[C@@H](CCC2)C(=O)N[C@H]1C[C@@H]1[C@H]([NH2+][C@@H]2[C@@H]1CCCC2)CC=C(C)C	-8.7	*Penicillium* sp.
51	**M51**	N1c2c(C(=C)N[C@H](C(=O)N3[C@H](C(=O)Nc4c(C(=O)N([C@H](C1=O)C)C)cccc4)CCC3)C)cccc2	-8.7	*Aspergillus versicolor* LZD-14-1
52	**M52**	c1ccc2c(c1)[nH+]c1c2cc([nH]c1C(=O)C)C(=O)NCCc1ccc(cc1)O	-8.7	*Marinactinospora thermotolerans* SCSIO 00652
53	**M53**	c1cc(c2c(c1)C(=O)c1c(C2=O)c(c2c(c1O)c1c(C[C@H]2O)cc2c(c1O)C(=O)O[C@@](C2)(O)C)OC)O	-8.7	*Streptosporangium* sp.
54	**M54**	c1cc(c2c(c1)C(=O)c1c(C2=O)c(c2c(c1O)c1c(C[C@H]2O)cc2c(c1O)C(=O)O[C@@](C2)(OC)C)OC)O	-8.7	*Streptosporangium* sp.
55	**M55**	C1[C@H](OC(=O)C[C@H](OC(=O)c2c(cc(cc2O)O)C[C@H](OC(=O)C[C@@H](C)OC(=O)c2c(cc(cc2O)O)CC1=O)C)C)C	-8.7	*Hansfordia sinuosae*
56	**M56**	Cc1cc(c2c(c1)[OH+]C1=C([C@@H]3[C@@H]4[C@H](C1)[C@](C(=O)OCC)(O)O[C@@]4(C1=C3[OH+]c3cc(C)cc(c3C1=O)O)C(=O)OC)C2=O)O	-8.7	*Penicillium purpurogenum* G59
57	**M57**	c1ccc2c(c1)[nH+]c1c2cc([nH]c1C(=O)C)C(=O)NCCc1ccc(cc1)OC	-8.6	*Streptomyces* sp. CMN-62
58	**M58**	c1ccc2c(c1)[nH+]c1c2cc([nH]c1C(=O)C)C(=O)NCCc1ccccc1	-8.6	*Marinactinospora thermotolerans* SCSIO 00652
59	**M59**	C1C[C@@H]([C@H]2[C@H]3[C@@H]1N[C@H](C(=O)N[C@@H](C[C@H]3C[NH2+]2)CC[C@H]1O[C@H]([C@@H]([C@H]([C@@H]1O)O)O)CO)C(CC)(C)C)C(C)(C)C=C	-8.6	*Marinactinospora thermotolerans* SCSIO 00652
60	**M60**	c1ccc2c(c1)N=C1[N+](C2=O)[C@H](C(=O)NC1)C[C@@]1(c2c(cccc2)N2[C@H]1N[C@H](C2=O)C)O	-8.6	*Aspergillus versicolor* LZD-14-1
61	**M61**	[C@@H]1(C[C@@H]([C@@H]2[C@@H](C1)[C@@H]([C@@H]([NH2+]2)C(C)(C)C=C)C[C@H]1C(=O)N[C@H](C(=O)N1)C)C/C=C(\C)/CO)CC=C(C)C	-8.6	*Eurotium cristatum*
62	**M62**	C1=C[C@H]2[C@H](OC1(C)C)C[C@@H]1[C@@H](C2)[C@@H]2C[C@]34[C@@H](C[C@]5(N(C3=O)CCC5)C(=O)N4)C([C@H]2[NH2+]1)(C)C	-8.6	*Aspergillus versicolor*
63	**M63**	c1cccc(c1O)C1=N[C@@H](CO1)[C@@H]1SC[C@@H](N1C)CN1[C@@H]2C(=O)N([C@H](C1=O)CSSC2)C[C@@H]1N([C@H](SC1)[C@H]1N=C(OC1)c1c(O)cccc1)C	-8.6	*Streptomyces olivaceus*

64	**M64**	C1[C@@H](C[C@]2([C@](C1)([C@@H]1[C@@H](CC2=O)[C@H]2[C@](CC1)([C@H](CC2)[C@@H](/C=C/[C@@H](C(C)C)C)C)C)O)O	-8.6	*Aspergillus terreus* GX7-3B
65	**M65**	CC(=CC[N@H+]1[C@H]2C[C@H](CC[C@H]2[C@H]2[C@@H]1[C@H](C=C(C)C)N1C(=O)[C@@H]3CCCN3C(=O)[C@@]1(C2=O)O)OC)C	-8.6	*Neosartorya laciniosa* KUFC 7896
66	**M66**	[C@@H]1([C@H](C[C@@H]2[C@H](C1)[C@@H](C[NH2+]2)[C@@H]1[C@H]([C@H]([NH2+]C1)C(=O)OC)[C@@H]1[C@@H]2[C@H]([NH2+]C1)CC[C@@H](C2)Cl)Cl)Cl	-8.6	*Marine actinomycete* NPS12745
67	**M67**	c1ccc2c(c1O)C(=O)c1c([OH+]2)cc(c2c1O[C@@H]1[C@H]2C=CO1)OC	-8.6	*Aspergillus* sp. MF-93
68	**M68**	c1ccc2c(c1)[C@@]1([C@@H]3N2C(=O)[C@H](N3C(=O)[C@H](C1)[N+]1C(=O)c2c(N=C1)cccc2)[C@H](CC)C)O	-8.5	*Aspergillus versicolor* LZD-14-1
69	**M69**	C1(=C2[C@]34[C@H](C(=O)[C@H](CC1)C)[C@H]([C@@H]1[C@@]3(OC2=O)C[C@@H]([C@]([C@H]1O)(C)O4)C)S[C@@H]1CC(=O)[C@H](CCC(=O)C2=C3[C@]4([C@@H]1[C@@H]([C@@]([C@H](C4)C)(C)O3)O)OC2=O)C)O	-8.5	*Streptomyces koyangensis* SCSIO 5802
70	**M70**	C1=Cc2c(OC1(C)C)cc1c(c2)[C@]2(C(=N1)C([C@H]1[C@]3(C2)C(=O)N2[C@](C1)(C(=O)N3)CCC2)(C)C)O	-8.5	*Aspergillus versicolor*
71	**M71**	c12cccc3c1c(c(=O)[n+]2C)c1c(c3)C([N+]2C1=NC(=CC2=O)C)(C)C	-8.5	*Penicillium commune* DFFSCS026
72	**M72**	[C@@H]1(CC[C@@H]2[C@H](C1)[C@H]([C@H]1[C@H]([C@@]2(C)O)OC2=C(C1=O)C(=O)[NH+]C=C2c1ccc(cc1)O)/C=C/C)C	-8.5	*Campylocarpon* sp. HDN13-307
73	**M73**	C/C/1=C/C[C@@H](/C(=C\[C@@H]2[C@H](C=C(C[C@]32C(=O)/C(=C(\[C@]2(C)[C@H]1C=C[C@H]1[C@H]2[C@@H](C)C[C@H](C)[C@@H]1O[C@H]1C[C@H]([C@H]([C@H](COC(=O)C)O1)OC(=O)C)O[C@H]1CC[C@H]([C@H](C)O1)O[C@@H]1C[C@H]([C@H]([C@H](C)O1)O[C@H]1CC[C@H]([C@H](C)O1)O)O)/O)/C(=O)O3)C=O)O)/C)O[C@H]1C[C@@](C)([C@H]([C@@H](C(=O)OC)O1)N)N(=O)=O	-8.5	*Micromonospora carbonacea* LS276
74	**M74**	Cc1cc(c2c(c1)[OH+]C1=C([C@@H]3[C@@H]4[C@H](C1)[C@](C(=O)OC)(O)O[C@@]4(C1=C3[OH+]c3cc(C)cc(c3C1=O)O)C(=O)OC)C2=O)O	-8.5	*Penicillium purpurogenum* G59
75	**M75**	C1C[C@H]2[C@H](CC1)[C@H](C[NH2+]2)[C@@]1([C@@H]2CCCC[C@@H]2NC1=O)C1=CN[C@H]2[C@@H]1C=CC=C2	-8.4	*Shewanella piezotolerans*
76	**M76**	c1ccc2c(c1)[C@@]1([C@@H]3N2C(=O)[C@H](N3C(=O)[C@H](C1)[N+]1C=Nc2c(C1=O)cccc2)[C@H](CC)C)O	-8.4	*Aspergillus versicolor* LZD-14-1
77	**M77**	c1(=C)c(=C)c(=C)[n+]2c([nH]1)[C@H](NC(=O)[C@H]2C[C@@]1([C@H]2N(c3c1cccc3)C(=O)[C@@H](N2)[C@@H](CC)C)O)C	-8.4	*Aspergillus versicolor* LZD-14-1
78	**M78**	C1=C[C@@]23C(=NC4=C(NC(=C)[C@@H](N4[C@@]42O[C@H](CO3)C(O4)(C)C)Cc2ccccc2)C(C)C)OC=C1	-8.4	*Paecilomyces variotii*
79	**M79**	C1=Cc2c(OC1(C)C)cc1c(c2)[C@@]2(C(=O)N1)C([C@@H]1[C@@]3(C2)C(=O)N2[C@@](C1)(C(=O)N3)CCC2)(C)C	-8.4	*Aspergillus versicolor*

80	**M80**	C1=C[C@H]2[C@H](OC1(C)C)C[C@H]1[C@@H](C2)[C@H]([C@H]([NH2+]1)C(C)(C=C)C)C[C@H]1C(=O)N2[C@@H](C(=O)N1)CCC2	-8.4	*Aspergillus versicolor*
81	**M81**	[C@@H]1(CC[C@H]2[C@H](C1)[C@H]([C@@]1(C=C2C)C(=O)C2=C(O1)C(=C[NH+]C2=O)c1ccc(cc1)O)/C=C/C)C	-8.4	*Campylocarpon* sp. HDN13-307
82	**M82**	C1[C@H]2[C@H]3[C@@]4(C(=O)N2)[C@H](C=C([C@]3([C@@H]2[C@@H]1[C@@H]1[C@H]([NH2+]2)CCCC1)C)C)/C=C/C[C@H](C)/C=C(/C(=O)C(=O)CCC4=O)\C	-8.4	*Penicillium chrysogenum* V11
83	**M83**	O1C(=O)CC[C@]2([C@](C1(C)C)([C@@H](C[C@]1([C@@H]2Cc2c(O1)c(c1c(c2OC)C(=O)OC1)C)C)O)O)C	-8.4	*Aspergillus aureolatus* HDN14-107
84	**M84**	C1CC(=O)C=C2[C@]1([C@@H]1C(=C3[C@](CC1)([C@H](CC3)[C@H](/C=C/C(=C)C(C)C)C)C)C=C2)C	-8.4	*Aspergillus* sp.
85	**M85**	c1cc(c2c(c1)C(=O)c1c(C2=O)c(c2c(c1O)c1c(C[C@H]2OC(=O)C(C)C)cc2c(c1O)C(=O)O[C@H](C2)C)OC)O	-8.4	*Streptosporangium* sp.
86	**M86**	c1cc(c2c(c1)C(=O)c1c(C2=O)c(c2c(c1O)c1c(C[C@H]2OC(=O)CC)cc2c(c1O)C(=O)O[C@H](C2)C)OC)O	-8.4	*Streptosporangium* sp.
87	**M87**	c12c(c(ccc1O)[C@@H]1CCC(=O)c3c1cccc3O)C(=O)[C@](C2=O)(CC(=O)C)O	-8.4	*Cladosporium cladosporioides* HDN14-342
88	**M88**	N1C(=O)[C@@H](N(C(=O)[C@@H](NC(=O)[C@@H]2N(C(=O)[C@@H](OC(=O)[C@@H](N(C(=O)[C@@H]1Cc1ccc(cc1)OCCC(=C)C)C)C)Cc1ccccc1)CCC2)C)C)[C@H](CC)C	-8.4	*Bionectria ochroleuca*
89	**M89**	C[C@H]1C/C=C/[C@H]2[C@H]3[C@@](C)([C@@H](C)[C@H]4[C@H](Cc5ccccc5)N=C([C@@]24C(=O)/C=C/C(=O)[C@@H](/C(=C/1)/C)OC(=O)C)O)O3	-8.4	*Jishengella endophytica* 161111
90	**M90**	Cc1cc(cc(c1C(=O)c1c2c3cc(cc(c3c(=O)c3c(cc(C)c(c23)[oH+]c1=O)O)O)O)O)O	-8.4	*Aspergillus glaucus*
91	**M91**	N1C(=O)[C@@]2(c3cc(c4c(c13)C=CC(O4)(C)C)Cl)C([C@H]1[C@]3(C(=O)N4CCC[C@]4(C1)C(=O)N3)[C@H]2O)(C)C	-8.4	*Aspergillus westerdijkiae* DFFSCS013
92	**M92**	c1c(c(ccc1)O)/C=C/1\NC(=O)[C@@]2(NC1=O)Cc1c(O2)cccc1	-8.4	*Hansfordia sinuosae*
93	**M93**	c1ccc2c3c1O[C@]14c5c(C(=O)[C@@H]6[C@H]1O6)c(ccc5O[C@]3([C@@H](CC2=O)O)O4)O	-8.4	*Lasiodiplodia theobromae* ZJ-HQ1
94	**M94**	c1cc(c2c3c1c1c4c(c3C=CC2=O)[C@H]([C@H](Oc4c(cc1)O)C(=O)O)O)O	-8.4	*Alternaria* sp.
95	**M95**	c1cc(c2c3c1c1c4c(c3C=CC2=O)C[C@](Oc4c(cc1)O)(C(=O)O)O)O	-8.4	*Alternaria* sp.
96	**M96**	Cc1cc(cc(c1C(=O)c1c2c3cc(cc(c3c(=O)c3c(cc(C)c(c23)[oH+]c1=O)O)O)O)O)O	-8.4	*Aspergillus glaucus*
97	**M97**	C[C@H]1C(=O)N2[C@@H]3N1[C@H]1C4=Nc5ccccc5C(=O)[N+]4[C@@H](C[C@]3(c3ccccc23)O)C(=O)N1	-8.4	*Aspergillus versicolor* MST-MF495
98	**M98**	c1(ccc(cc1)C[C@@H](C(=O)N[C@H](C(=O)N[C@@H](C(=O)O)C(C)C)C[C@H]1[C@@H]2[C@H]([NH2+]C1)CCCC2)N)O	-8.3	*Aspergillus* sp. SCSIO 41501
99	**M99**	c1ccc2c(c1)c1c([nH+]2)c([nH]c(c1)C(=O)O)c1cc(oc1)CO	-8.3	*Jishengella endophytica* 161111
100	**M100**	c1ccc2c(c1)N=C1[N+](C2=O)[C@H]2C[C@]3([C@@H]4N([C@@H]1NC2=O)[C@H](C(=O)N4c1c3cccc1)CO)O	-8.3	*Aspergillus versicolor* LZD-14-1

101	**M101**	[C@@H]1(CC[C@H]2[C@H](C1)[C@H]([C@]1(C=C2C)C(=O)C2=C(O1)C(=C[NH+]C2=O)c1ccc(cc1)O)/C=C/C)C	-8.3	*Campylocarpon* sp. HDN13-307
102	**M102**	[C@@H]1(CC[C@@H]2[C@H](C1)[C@H]([C@H]1[C@H]([C@]2(C)O)OC2=C(C1=O)C(=O)[NH+]C=C2c1ccc(cc1)O)/C=C/C)C	-8.3	*Campylocarpon* sp. HDN13-307
103	**M103**	c12c(c(ccc1O)[C@@H]1CCC(=O)c3c1cccc3O)C(=O)[C@H]([C@@H]([C@@H]2OC)O)Cl	-8.3	*Cladosporium cladosporioides* HDN14-342
104	**M104**	c12c(cc(cc1[OH+]C1=C(C2=O)[C@]2([C@H]3[C@@H]1C1=C(C[C@H]3[C@@](O2)(C(=O)OC)O)[OH+]c2c(C1=O)c(cc(c2)C)O)C(=O)OC)C)O	-8.3	*Penicillium* sp. ZJ-SY2
105	**M105**	[C@@]123[C@H]([C@@]4(c5ccccc5N[C@@H]4N1C(=O)[C@](SS2)(N(C3=O)C)[C@H](C)O)[C@]12[C@@H]([C@]34N([C@H]2Nc2c1cccc2)C(=O)[C@](SS3)(N(C4=O)C)[C@H](C)O)O)O	-8.3	*Bionectria ochroleuca*
106	**M106**	[C@@H]12C[C@H]3[C@@H]4CC[C@@H](C[C@@H]4[NH2+][C@@H]3[C@@H](N1C(=O)[C@@H]1CCCN1C2=O)C=C(C)C)OC	-8.3	*Aspergillus* sp. BRF 030
107	**M107**	C1[C@@H](C[C@]2([C@](C1)([C@@]1([C@@H](CC2=O)[C@@H]2[C@](CC1)([C@H](CC2)[C@@H](/C=C/[C@@H](C(C)C)C)C)C)O)C)O)O	-8.3	*Aspergillus versicolor* ZBY-3
108	**M108**	c1ccc2c3c1O[C@]14c5c(C(=O)[C@H]([C@H]1O)Cl)c(ccc5O[C@]3([C@@H](CC2=O)OC)O4)O	-8.3	*Lasiodiplodia theobromae* ZJ-HQ1
109	**M109**	c1ccc2c3c1O[C@]14c5c(C(=O)[C@@H]6[C@H]1O6)c(ccc5O[C@]3(CCC2=O)O4)O	-8.3	*Lasiodiplodia theobromae* ZJ-HQ1
110	**M110**	c1ccc2c3c1O[C@]14c5c(C(=O)[C@@H]6[C@H]1O6)c(ccc5O[C@]3(C=CC2=O)O4)O	-8.3	*Lasiodiplodia theobromae* ZJ-HQ1
111	**M111**	[C@@H]1(CC[C@@H]2[C@H](C1)[C@@H](C[NH2+]2)[C@@H]1[C@H]([C@H]([NH2+]C1)C(=O)OC)[C@@H]1[C@@H]2[C@H]([NH2+]C1)CC[C@@H](C2)Cl)Cl	-8.3	*Marine actinomycete* NPS12745
112	**M112**	N1([C@H](C(=O)Nc2c(C(=O)N3[C@H](C(=O)Nc4c(C(=O)N[C@H](C1=O)C)cccc4)CCC3)cccc2)C)C	-8.2	*Aspergillus versicolor* LZD-14-1
113	**M113**	C1(=O)C[C@H]([C@@H]2[C@@]34C(=C(C(=O)CC[C@@H]1C)C(=O)O3)O[C@@]([C@H](C4)C)([C@H]2O)C)[C@@H]1C(=O)C(=C(O1)O)C	-8.2	*Streptomyces koyangensis* SCSIO 5802
114	**M114**	C1CC[C@@H]2[C@H](C1)[C@@H]([C@H]([NH2+]2)C(C)(C=C)C)/C=C\1/[NH+]C(=O)/C(=C/C)/[NH+]C1=O	-8.2	*Eurotium* sp. SCSIO F452
115	**M115**	C1=Cc2c(OC1(C)C)cc1c(c2)[C@](C(=O)N1)(C[C@H]1C(=O)N2[C@H](C(=O)N1)CCC2)C(C)(C)C=C	-8.2	*Aspergillus versicolor*
116	**M116**	c12cccc3c1[C@@](C(=O)N2)([C@H]1[C@@H](C3)CNC1=O)O	-8.2	*Penicillium commune* DFFSCS026
117	**M117**	C1([C@@H](CC[C@]2([C@@H]1CC[C@@]1([C@@H]2Cc2c(O1)c(c1c(c2O)C(=O)OC1)C)C)C)O)(C)C	-8.2	*Aspergillus aureolatus* HDN14-107
118	**M118**	[C@@H]1(C([C@H]2[C@](CC1)([C@@]1([C@@H](CC2)C)Oc2c(C1)c(cc1c2CN(C1=O)CCCC(=O)OC)O)C)(C)C)O	-8.2	*Stachybotrys chartarum* 952

119	**M119**	C1(=C([C@H](OC1=O)O)c1ccc(cc1)O)Cc1ccc2c(c1)CCC(O2)(C)C	-8.2	*Aspergillus terreus*
120	**M120**	N12C(=O)[C@H](OC(=O)[C@@H](N(C(=O)[C@@H](NC(=O)[C@@H](N(C(=O)[C@@H](NC(=O)[C@@H]1CCC2)CO)C)C(C)C)Cc1ccc(cc1)OCC=C(C)C)C)C)Cc1ccccc1	-8.2	*Bionectria ochroleuca*
121	**M121**	CC[C@@H](C)[C@@H]1C(=O)N(C)[C@H]([C@H](c2ccccc2)O)/C(=N/c2ccccc2/C(=N/[C@H](C)/C(=N/1)/O)/O)/O	-8.2	*Aspergillus terreus* SCSGAF0162
122	**M122**	C[C@]1(c2cccc(c2C(=C2[C@@H]1C[C@H]1[C@@H](C(=O)C(=C([C@]1(C2=O)O)O)C(=N)O)N(C)C)O)O)O	-8.2	*Streptomyces* BL 567201
123	**M123**	C1C2=C[C@@H]([C@@H]3[C@@H]([C@]2(CC[C@@H]1O)C)CC[C@]1([C@H]3CC[C@H]1[C@@H](/C=C/[C@H](C(C)C)C)C)C)O	-8.1	*Aspergillus* sp.
124	**M124**	[C@H]1(C(=O)NCC(=O)N([C@H](C(=O)N[C@H](C(=O)N1C)C)c1ccccc1)C)Cc1ccccc1	-8.1	*Aspergillus versicolor* LZD-14-1
125	**M125**	c1(ccccc1)C[C@@H](N(C(=O)[C@@H](N)[C@@H](CC)C)C)C(=O)N[C@H](C(=O)Nc1ccccc1C(=O)O)C(C)C	-8.1	*Simplicillium obclavatum* EIODSF 020
126	**M126**	N1[C@H](C2=Nc3c(C(=O)[N+]2[C@@H]2[C@@H](C1=O)C=CC=C2)cccc3)CCC(=O)N	-8.1	*Penicillium aurantiogriseum*
127	**M127**	c1ccc2c(c1)[C@@]1(C(=N2)C([C@@H]2[C@@]3(C1)CN1[C@@](C2)(O[NH2+]3)CCC1)(C)C)O	-8.1	*Penicillium purpurogenum* G59
128	**M128**	c1ccc2c(c1)[C@@]13C(=N2)C([C@H]2[C@](C1)([C@@H](N1[C@H](C2)CCC1)C#N)NC(=O)O3)(C)C	-8.1	*Aspergillus versicolor*
129	**M129**	C(C[C@@H](CCCCCC/C=C/C(=O)N[C@@H]1[C@H](O[C@@H]([C@@H]([C@@H]1O)O)C[C@@H](O)[C@@H]1[C@@H]([C@@H]([C@@H](O1)[N+]1C(=O)[NH+]C(=O)C=C1)O)O)O[C@@H]1O[C@H]([C@@H]([C@@H]([C@@H]1NC(=O)C)O)O)CO)C)C	-8.1	*Streptomyces xinghaiensis* SCSIO S15077
130	**M130**	C[C@@H]1C2=Nc3c(cccc3O)C(=O)[N+]2c2ccccc2C(=O)N1	-8.1	*Aspergillus westerdijkiae* SCSIO 05233
131	**M131**	CC(C)C[C@@H](c1ccc2c(c1OC)C(=O)OCc1cc(C)cc(c1O2)O)O	-8.1	*Penicillium* sp. ZLN29
132	**M132**	C1CC[C@H]2[C@H](C1)[C@H](C[N@H+]2C)C(=O)N1CCN(CC1)[C@@H]1[C@H](CCCC1)OC	-8.1	*Aspergillus sydowii* SCSIO 00305
133	**M133**	COc1ccc2C=C(C(=O)[OH+]c2c1OC)NC(=O)C1=NO[C@H]2[C@@H](C=C[C@H]([C@]2(C1)O)Cl)O	-8.1	*Trichoderma virens*
134	**M134**	C1(=C)C[C@@H]2[C@H]([C@H]3[C@@H]1C[C@@H]3C(O)(C)C)[C@H]1[C@@H](CC2)[NH2+][C@@H]2[C@H]1[C@H]([C@H]1[C@]2([C@@]2([C@@](CC1)([C@]13[C@H](CC2)O[C@@H]([C@@H]([C@H]1O3)O)C(=C)C)O)C)C)OC	-8.1	*Mucor irregularis* QEN-189
135	**M135**	CC[C@H](C)C(=O)O[C@H]1CCC=C2C=C[C@H](C)[C@H](CC[C@@H]3C[C@H](CC(=O)O3)O)[C@@H]12	-8.1	*Penicillium solitum*
136	**M136**	c1(ccccc1)C[C@H]1N(C(=O)[C@@H](OC(=O)[C@@H](NC(=O)[C@@H](OC(=O)[C@@H](N(C(=O)[C@H](NC1=O)C)C)C)C(C)(C)O)C)CC(C)C)C	-8.1	*Spicellum roseum*

137	**M137**	COc1ccc2c(c1)[nH]c1c2[C@@H]([C@@]2(N([C@H]1C=C(C)C)C(=O)[C@@H]1CCCN1C2=O)O)O	-8	*Aspergillus* sp.
138	**M138**	c1(ccc(cc1)O)C[C@@H](C(=O)N[C@H](C(=O)N[C@@H](C(=O)N[C@@H](C(=O)O)C(C)C)C(C)C)C)N	-8	*Aspergillus* sp.
139	**M139**	c1(ccccc1)C[C@H](N(C(=O)[C@@H](N)C(C)C)C)C(=O)N[C@H](C(=O)Nc1ccccc1C(=O)O)CC(C)C	-8	*Simplicillium obclavatum* EIODSF 020
140	**M140**	c1cc(ccc1)C[C@@H]1NC(=O)CN(C(=O)[C@H]2N(C(=O)[C@H](N(C(=O)[C@@H](NC(=O)[C@@H](NC(=O)[C@@H](N(C1=O)C)Cc1ccc(cc1)O)C(C)C)CC(C)C)C)Cc1ccccc1)CCC2)C	-8	*Acremonium persicinum* SCSIO115
141	**M141**	c1ccc2c(c1)c1c([nH+]2)c([nH]cc1)c1cc(oc1)CO	-8	*Jishengella endophytica* 161111
142	**M142**	c1ccc(c(c1)C(=O)OC)[N+]1C(=Nc2c(C1=O)cccc2)[C@@H]1CCC(=[NH2])N1	-8	*Penicillium aurantiogriseum*
143	**M143**	C1[C@@H](C[C@H]2[C@@H](C1)[C@H]([C@H]([NH2+]2)C(C)(C)C=C)C[C@@H]1C(=O)N[C@@](C(=O)N1)(C)OC)CC=C(C)C	-8	*Eurotium cristatum*
144	**M144**	O1[C@@H](CC(=O)O[C@@H](CC(=O)Cc2c(C1=O)c(cc(c2)O)O)C)C	-8	*Hansfordia sinuosae*
145	**M145**	c12c(c(ccc1O)[C@@H]1CCC(=O)c3c1cccc3O)C(=O)CC[C@@H]2OC	-8	*Cladosporium cladosporioides* HDN14-342
146	**M146**	c1c(cc2c(c1O)C(=O)c1c(C2=O)cc(cc1O)O)C[C@H](O)C	-8	*Penicillium* sp. SCSGAF 0023
147	**M147**	c1ccc2c(c1O)C(=O)c1c(C2=O)cc(c(c1O)[C@@H](CCO)CO)O	-8	*Aspergillus* sp. F40
148	**M148**	c1cc(c2c(c1)[OH+]c1c(C2=O)c(cc2c1[C@]1([C@@](O2)(O[C@@H](C1)OC)C)C)OC)O	-8	*Aspergillus* sp. F40
149	**M149**	C1C[C@](c2c(C1)c1c(c(c2)O)[C@@H]2[C@](CC1)([C@@H](CO2)O)C)(C)CO	-8	*Aspergillus wentii* SD-310
150	**M150**	C1C[C@](c2c(C1)c1c(c(c2)O)[C@@H]2[C@](CC1)([C@H](CO2)O)C)(C)CO	-8	*Aspergillus wentii* SD-310
151	**M151**	C[C@H]1C[C@H]2[C@H](C)Oc3c(cnc(c3[C@@H]2[C@@H](C)[C@@H]1O)O)c1ccc(cc1)O	-8	*Chaunopycnis* sp. CMBMF028
152	**M152**	N1C(=O)[C@@]2(c3ccc4c(c13)C=CC(O4)(C)C)C([C@H]1[C@]3(C(=O)N4CCC[C@]4(C1)C(=O)N3)[C@@H]2O)(C)C	-8	*Aspergillus westerdijkiae* DFFSCS013
153	**M153**	c1ccc2c(c1)C1=C(C2)[C@]2([C@H](C1)CC[C@@H]1[C@@]2(CC[C@H]2C1=C[C@H]([C@H](C2)C(C)(C)O)O)C)C	-8	*Mucor irregularis* QEN-189
154	**M154**	C[C@@H]1CCC/C=C\[C@@H]2CCC[C@H]2[C@H](/C=C\C(=O)O1)O	-8	*Penicillium* sp. PSU-F44
155	**M155**	O=c1c2ccccc2ncn1[C@H]1C(=O)N([C@@H]2[C@@](C1)(O)c1ccccc1N2)[C@H](C)C(=O)OC	-8	*Penicillium* sp.
156	**M156**	c12c(cccc1)c(c[nH]2)C[C@H]1C(=O)N2[C@H](C(=O)N1)CCC2	-7.9	*Aspergillus* sp.
157	**M157**	C1C[C@@H]2[C@H](CC1)[C@@H](C[NH2+]2)[C@]1([C@@H]2CCCC[C@H]2NC1=O)OC	-7.9	*Shewanella piezotolerans*
158	**M158**	c1ccc2c(c1)N=C1[N+](C2=O)[C@H](C(=O)NC1)C[C@]12c3c(cccc3)N3[C@H]1N(C1(C3=O)CC1)CO2	-7.9	*Aspergillus versicolor* LZD-14-1

159	**M159**	[C@H]1([C@]23N([C@@H]4[C@]1(c1c(N4)cccc1)[C@@]14c5c(N[C@@H]1N1[C@]6([C@H]4O)C(=O)N([C@@](C1=O)(CO)[S@@]6=S=S)C)cccc5)C(=O)[C@@](N(C2=O)C)(C)[S@]3=S=S)O	-7.9	*Acrostalagmus luteoalbus* HDN13-530
160	**M160**	C1CC[C@H]2[C@@H](C1)[C@H](C[N@H+]2C/C=C(\C)/CO)C[C@H]1C(=O)N[C@H](C(=O)N1)C	-7.9	*Eurotium cristatum*
161	**M161**	c12cccc3c1[C@@]1(C(=O)N2C)[C@H]2[C@@H](C3)C(N3[C@]2([C@](O1)([C@](C3=O)([C@H](C)O)O)O)O)(C)C	-7.9	*Penicillium commune* DFFSCS026
162	**M162**	C1CC[C@H]2[C@H](C1)[C@H]1[C@H]([NH2+]2)C([C@@H]2[C@]3(C1)CN1[C@@](C2)(O[NH2+]3)CCC1)(C)C	-7.9	*Penicillium purpurogenum* G59
163	**M163**	[C@H]1([C@H]([C@H]([C@@H]([C@H]2[C@@]1(C=C([C@H]([C@@H]2/C=C(/C(=O)O)\C)/C(=C/C[C@@H](C[C@H]([C@@H]([C@H](C(C)C)O)C)O)O)/C)C)C)C)O)O)O	-7.9	*Streptomyces* sp. SCSGAA 0027
164	**M164**	[C@H]1(CC[C@@H]([C@H]2[C@@]1(C=C([C@H]([C@@H]2/C=C(/C(=O)O)\C)/C(=C/C[C@@H](C[C@H]([C@@H]([C@H](C(C)C)O)C)O)O)/C)C)C)C)O	-7.9	*Streptomyces* sp. SCSGAA 0027
165	**M165**	c1(c(cc2c(c1C)c(c1c(c2)[C@@H](OC1=O)[C@]1([C@@H](C(=CC(=O)[C@@H]1OC)OC)O)O)O)OC)C(=O)OC	-7.9	*Saccharothrix* sp. 10-10
166	**M166**	C1(=C([C@H](OC1=O)OC)c1ccc(cc1)O)Cc1ccc(c(c1)CC=C(C)C)O	-7.9	*Aspergillus terreus*
167	**M167**	c1cc(c2c(c1)C(=O)c1c(C2=O)c(c2c(c1O)c1c(C[C@H]2O)cc(c(c1O)C(=O)O)CC(=O)C)OC)O	-7.9	*Streptosporangium* sp.
168	**M168**	c1c(cc2c(c1O)[OH+]C(=CC2=O)c1c(c(cc(c1)O)O)O)O	-7.9	*Penicillium* sp. SCSGAF 0023
169	**M169**	C1(=O)[C@@]([C@@H]([C@@H]2C(=C1)C=C(OC2)/C=C/[C@@]([C@H]([C@H](CC)C)O)(O)C)O)(O)C	-7.9	*Penicillium sclerotiorum* M-22
170	**M170**	C(=O)(c1ccc(cc1)[N](=O)O)O[C@H]1[C@@H]2[C@@]([C@@]3(C(=C1)C(=O)OC3)O)(CCC[C@]2(C)CO)C	-7.9	*Aspergillus ochraceus* Jcma1F17
171	**M171**	N1C(=O)[C@@H](NC(=O)/C/1=C/[C@@H]1[C@H]([NH2+][C@H]2[C@@H]1CCCC2)C(C)(C=C)C)C	-7.9	*Microsporum* sp. MFS-YL
172	**M172**	c1ccc2c3c1O[C@]14c5c([C@H]([C@@H]6[C@H]1O6)O)c(ccc5O[C@]3(C=CC2=O)O4)O	-7.9	*Lasiodiplodia theobromae* ZJ-HQ1
173	**M173**	C1[C@@H](C(c2c(C1)c1c(c(c2)O)[C@@H]2[C@](CC1)([C@@H](CO2)O)C)(C)C)O	-7.9	*Aspergillus wentii* SD-310
174	**M174**	C1CC(c2c(C1)c1c(c(c2)O)[C@@H]2[C@](CC1)([C@@H](CO2)O)C)(C)C	-7.9	*Aspergillus wentii* SD-310
175	**M175**	COc1cc2cc3COC(=O)c3c(c3ccc4c(c3)OCO4)c2cc1OC	-7.9	*Nocardia* sp. ALAA 2000
176	**M176**	CC(=C[C@H]1[C@]2(C=C3C(=O)N4CCC[C@H]4C(=O)N13)c1ccc(cc1N=C2O)OC)C	-7.9	*Aspergillus sydowi* PFW1-13
177	**M177**	[C@@H]1(O)CC2=CC(=O)C3=C([C@]2(CC1)C)CC[C@]1([C@@H]3CC[C@@H]1[C@@H](/C=C/[C@@H](C(C)C)C)C)C	-7.8	*Aspergillus* sp.
178	**M178**	CC/C=C\[C@@H]([C@@H](C1=C(C(=O)[C@]2(O1)C(=O)N([C@]([C@@H]2O)(C(=O)c1ccccc1)OC)C(=O)Cc1ccccc1)O)O)O	-7.8	*Aspergillus* sp.

179	**M179**	C1(=O)[C@H](NC(=O)CC(=O)/C=C/C[C@H](OC(=O)[C@@H]2N1CCC2)CCC)Cc1ccccc1	-7.8	*Sarocladium kiliense* HDN11-112
180	**M180**	C1(=O)[C@H](N(C(=O)[C@@H](NC(=O)[C@@H](NC(=O)[C@H]2N(C(=O)[C@@H](N1)C(C)C)CCC2)Cc1ccc(cc1)OC)Cc1ccc(cc1)OC)C)Cc1ccc(cc1)O	-7.8	*Aspergillus* sp. SCSIO 41501
181	**M181**	c12cccc3c1c(c(=O)[nH+]2)c1c(c3)C(N(C1=O)C(=O)CC(=O)C)(C)C	-7.8	*Penicillium commune* DFFSCS026
182	**M182**	c12cccc3c1c(c(=O)[nH+]2)c1c(c3)C(NC1=O)(C)C	-7.8	*Penicillium commune* DFFSCS026
183	**M183**	c1cc(ccc1C(=O)OC[C@@]1([C@H]2[C@](CCC1)([C@@]1([C@@H](C[C@H]2O)C(=O)OC1)O)C)C)[N](=O)O	-7.8	*Aspergillus ochraceus* Jcma1F17
184	**M184**	c1(c(cc2c(c1C)c(c1c(c2)[C@H](OC1=O)[C@]1([C@@H](C(=CC(=O)[C@@H]1O)OC)O)O)O)OC)C(=O)OC	-7.8	*Saccharothrix* sp. 10-10
185	**M185**	[C@@H]1(CC[C@@H](O[C@@H]1C)O[C@H]1[C@](C[C@@H](O[C@@H]1C)O[C@H]1[C@@H]([C@H](O[C@H](C1)c1c(c2c(cc1)C(=O)C1=C(C2=O)[C@@H]([C@@H]([C@@]2([C@]1([C@H](C=C(C2)C)O[C@H]1O[C@H]([C@H]([C@@H](C1)OC)O)C)O)O)CC(=O)C)C)O)C)O)(O)C)OC(=O)N	-7.8	*Streptomyces pratensis* NA-ZhouS1
186	**M186**	C1(=O)OC(C2=CC(=O)[C@]3([C@H]([C@@]4(C2=C1)CO4)C[C@@]1([C@@H]2[C@H]3C(=O)[C@H](O[C@@H]2OC1=O)C)C)C)(C)C	-7.8	*Talaromyces amestolkiae* YX1
187	**M187**	c1c(cc(c(c1CCC(=O)/C=C/C)C(=O)O[C@@H](CC(=O)O[C@@H](Cc1c(c(cc(c1)O)O)C(=O)O[C@@H](CC(=O)O[C@@H](Cc1cc(cc(c1)O)O)C)C)C)C)O)O	-7.8	*Hansfordia sinuosae*
188	**M188**	c12c(c(ccc1O)[C@@H]1CCC(=O)c3c1cccc3O)C(=O)[C@@](C2=O)(CC(=O)C)O	-7.8	*Cladosporium cladosporioides* HDN14-342
189	**M189**	[C@@H]1([C@](C[C@H]2[C@]3(C([C@H](CC[C@]13C)Cl)(C)C)O2)(C)OCc1c(c2c([C@H](OC2=O)OC)c(c1O)C=O)OC)C(=O)O	-7.8	*Acremonium strictum*
190	**M190**	C12=CC(=O)OC(C1=C[C@@H]1[C@]3([C@H]([C@H]2C)C[C@@]2([C@@H]4[C@]3([C@@]([C@@H](O[C@@H]4OC2=O)C)(O1)O)C(=O)OC)C)C)(C)C	-7.8	*Penicillium* sp. 303
191	**M191**	[C@]12([C@H]([C@H]3[C@@H]4CC[C@@H](C[C@@H]4[NH2+][C@@H]3[C@@H](N1C(=O)[C@H]1CCCN1C2=O)C=C(C)C)OC)O)O	-7.8	*Aspergillus* sp. BRF 030
192	**M192**	CC(=CCc1ccc2c(c1OC)C(=O)OCc1cc(C)cc(c1O2)O)C	-7.8	*Penicillium* sp. ZLN29
193	**M193**	[C@@H]12[C@H]3CC[C@@H]([C@]3(CC[C@H]1[C@]1(CC[C@@H](C[C@]31O[C@H]3C2=O)O)C)C)[C@H](C)/C=C/[C@H](C(C)C)C	-7.8	*Paecilomyces lilacinus* ZBY-1
194	**M194**	c1(cc(cc2C(=O)c3cc(cc(c3C(=O)c12)O)C)OC)O	-7.8	*Microsporum* sp. MFS-YL
195	**M195**	[C@@H]12OC3=C(C(=O)[NH+]C=C3c3ccc(cc3)O)C(=O)[C@@H]1[C@@H]([C@H]1C[C@H](CC[C@@H]1[C@]2(C)O)C)/C=C/C	-7.8	*Campylocarpon* sp. HDN13-307
196	**M196**	c1(ccccc1O)C(=O)[C@@H]1[N@H+]([C@H]([C@H](C1)Cl)Cl)[C@@H]1[C@H]([C@H]([NH2+][C@@H]1C(=O)[C@H]1CCCC[C@@H]1O)Cl)Cl	-7.8	*Actinomycete* strain CNQ-418
197	**M197**	CC(=CC(=O)[C@@H]1[C@H](C[C@H]2C(=O)N3CCC[C@H]3C(=N2)O)[C@@H]2CC[C@@H](C[C@@H]2[NH2+]1)OC)C	-7.8	*Aspergillus sydowi* PFW1-13

198	**M198**	O=c1c2ccccc2ncn1[C@H]1C(=O)N([C@@H]2[C@@](C1)(O)c1ccccc1N2)[C@H](C)C(=O)O	-7.8	*Penicillium* sp.
199	**M199**	O[C@@H]1CC2=CC=C3[C@H]4[C@@]([C@H](CC4)[C@H](C)/C=C/[C@@H](C(C)C)C)(CC[C@@H]3[C@]2(CC1)C)C	-7.7	*Aspergillus* sp.
200	**M200**	C[C@@H]1CCC/C=C/C=C/[C@@H](C[C@H]([C@@H](/C=C\C=C\[C@@H](C/C=C/C=C\C(=O)O1)O)O)O)O	-7.7	*Bacillus amyloliquefaciens* SCSIO 00856
201	**M201**	C[C@@H]1[C@H](OC=C2C1=C(C(=C(C2=O)CC1=C(C(=C([OH+]C1=O)C)C)O)O)C)C	-7.7	*Aspergillus* sp. 16-02-1
202	**M202**	[C@@H]12[C@H](CC(=O)[C@H](CCC1=O)C)[C@@H]1[C@@]3(OC2=O)C[C@@H]([C@]([C@H]1O)(C)OC3=O)C	-7.7	*Streptomyces koyangensis* SCSIO 5802
203	**M203**	c12c(cccc1)N=C([N+](C2=O)c1c(cccc1)C(=O)O)[C@H]1NC(=[NH2])CC1	-7.7	*Penicillium aurantiogriseum*
204	**M204**	C[C@H]1/C=C/C(=O)O[C@H](C)[C@H](/C=C\C=C\C(=O)[C@](C)(C[C@H](C)[C@@H]1O[C@H]1[C@@H]([C@H](C[C@@H](C)O1)OC)O)O)CO[C@H]1[C@@H]([C@@H]([C@@H]([C@@H](C)O1)O)OC)OC	-7.7	*Streptomyces* sp. HK-2006-1
205	**M205**	c12cccc3c1[C@@]1(C(=O)N2C)[C@H]2[C@@H](C3)C(N3[C@]2([C@](O1)([C@](C3=O)(CC(=O)C)O)O)O)(C)C	-7.7	*Penicillium commune* DFFSCS026
206	**M206**	c1(c(cc2O[C@@]3([C@@H]([C@@H]4c2c1[C@@H]([C@H](O4)C)C)C)C(=O)/C(=C(\[C@@H](CCCCCC)C)/O)/C(=O)N3C)O)C	-7.7	*Penicillium citrinum*
207	**M207**	c1ccc2c(c1)N=C([NH+]C2=O)[C@]([C@](C(=O)Nc1c(cccc1)C(=O)N)(C)O)(O)C	-7.7	*Penicillium commune* 366606
208	**M208**	C1(=C[C@@H]2[C@]3(CC1)[C@@]1([C@]4([C@H](O2)C[C@H]1OC(=O)C/C=C/C=C(/OCC/C(=C/C(=O)OC3)/C)\C(=O)C)CO4)C)C	-7.7	*Stachybotrys chartarum*
209	**M209**	[C@]12([C@H]([C@H](C[C@H]1O)C(C)C)C[C@]1([C@H](C2)[C@H](CC[C@@H]2C3=C1C(=O)O[C@]3(C[C@@H]2C)C)C)O)C	-7.7	*Alternaria alternata* k21-1
210	**M210**	O1[C@]2(CC[C@]3([C@](C1(C)C)([C@@H](C[C@]1([C@@H]3Cc3c(O1)c(c1c(c3OC)C(=O)OC1)C)C)C(=O)OC)O2)C)O	-7.7	*Aspergillus aureolatus* HDN14-107
211	**M211**	O1[C@H]([C@H]([C@H](C[C@@H]1O[C@@H]1C[C@@](O[C@@H]([C@H]1CC)C)([C@H]([C@@H]([C@@H]([C@@H]1[C@H](/C=C/C=C/C(=C)O[C@@H]([C@H](/C=C/C=C/C(=O)O1)C)[C@H]([C@H]([C@@H]([C@]1(C[C@H]([C@@H]([C@H](O1)C)CC)O[C@H]1C[C@@H]([C@@H]([C@@H](O1)C)O)O)O)C)O)C)C)C)O)C)O)O)O)C	-7.7	*Streptomyces* sp. 7-145
212	**M212**	[C@@H]1(CC(=C2[C@]([C@H]1O)(Oc1c(C2=O)c(c(cc1)c1ccc2c(c1O)C(=O)C1=C(C[C@@H]([C@@H]([C@@]1(O2)C(=O)OC)O)C)O)O)C(=O)OC)O)C	-7.7	*Penicillium* sp. SCSGAF 0023
213	**M213**	C1[C@@H](C[C@@]23[C@](C1)([C@@]1(C(=C4[C@](CC1)([C@H](C[C@@H]4O)[C@@H](/C=C/[C@@H](C(C)C)C)C)C=C2)OO3)C)O	-7.7	*Aspergillus niger* MA-132
214	**M214**	C[C@@]1(Cc2c(C[C@H]1O)c(c1C(=O)C=C(C(=O)c1c2O)OC)O)O	-7.7	*Nigrospora* sp. 1403
215	**M215**	C(=O)([C@H](CC(C)C)NC(=O)[C@H](Cc1ccc(cc1)O)N(C)C(=O)[C@H]([C@@H](CC)C)NC(=O)C)OC	-7.7	*Simplicillium obclavatum* EIODSF 020e

216	**M216**	c1cc(ccc1[N](=O)O)C(=O)OC[C@@]1([C@H]2[C@](CCC1)([C@@]1([C@@H](C[C@H]2O)C(=O)OC1)O)C)C	-7.7	*Aspergillus ochraceus* Jcma1F17
217	**M217**	C[C@]1([C@@H](C[C@H]2[C@@H]([C@H]1O)C(=O)c1c(C2=O)c(cc(c1)OC)O)O)O	-7.7	*Stemphylium globuliferum*
218	**M218**	c1(ccccc1O)C(=O)[C@@H]1[N@H+]([C@H]([C@H]([C@@H]1Br)Cl)Cl)[C@@H]1[C@H]([C@H]([NH2+][C@@H]1C(=O)[C@H]1CCCC[C@@H]1O)Cl)Cl	-7.7	*Actinomycete* strain CNQ-418
219	**M219**	CC[C@H](C)C(=O)O[C@H]1C[C@@H](C)C=C2C=C[C@H](C)[C@H](CC[C@@H]3C[C@H](CC(=O)O3)O)[C@@H]12	-7.7	*Aspergillus terreus*
220	**M220**	O1[C@@]2(Oc3cccc4c3c1ccc4O)[C@@H]1[C@@H]([C@@H](c3c2c(ccc3O)O)O)O1	-7.7	*Ascochyta* sp. NGB4
221	**M221**	C/C=C/C=C/C=C/C(=O)O[C@@H]1C=C2COC(=O)[C@@]2([C@@]2(C)CCCC(C)(C)[C@H]12)O	-7.7	*Aspergillus ustus*
222	**M222**	C1(=O)N[C@@H](C(=O)N2[C@H]1CCC2)Cc1ccccc1	-7.6	*Aspergillus* sp.
223	**M223**	[C@@H]1(O)C[C]23=C([C@H](O)C4=C([C@]2(CC1)C)CC[C@]1([C@@H]4CC[C@@H]1[C@@H](/C=C/[C@@H](C(C)C)C)C)C)O3	-7.6	*Aspergillus* sp.
224	**M224**	O=C1N2[C@H]([C@]3(C=C2C(=O)N2[C@H]1CCC2)c1c(NC3=O)cc(OC)cc1)C=C(C)C	-7.6	*Aspergillus* sp.
225	**M225**	c12c(cc(cc1[OH+]C1=C(C2=O)[C@]2([C@H]3[C@@H]1C1=C(C[C@@H]3[C@](O2)(C(=O)OC)O)[OH+]c2c(C1=O)c(cc(c2)C)O)C(=O)OC)C)O	-7.6	*Penicillium* sp. ZJ-SY2
226	**M226**	C(=O)(/C=C/[C@@H]1[C@@]23C(=C(C(=O)CC[C@H](C)O)C(=O)O2)O[C@@]([C@H](C3)C)([C@H]1O)C)O	-7.6	*Streptomyces koyangensis* SCSIO 5802
227	**M227**	c1(c(cc2c(c1)nc1c(n2)C(=O)[NH+]C(=O)[NH+]1)C)C	-7.6	*Jishengella endophytica* 161111
228	**M228**	C1C[C@H]2[C@@H](CC1)[C@@H](C[NH2+]2)[C@@]1([C@H]2CCCC[C@@H]2NC1=O)OC	-7.6	*Shewanella piezotolerans*
229	**M229**	c1ccc2c(c1O)N=C1[N+](C2=O)[C@H](C(=O)NC1)C[C@H]1[C@H]2[C@@H](CCCC2)[NH2+]C1	-7.6	*Aspergillus versicolor* LZD-14-1
230	**M230**	C[C@H]1CCC(=O)O[C@H](C)[C@@H](CO[C@H]2[C@@H]([C@@H]([C@@H]([C@@H](C)O2)O)OC)OC)[C@H]2[C@H](/C=C/C(=O)[C@](C)(C[C@H](C)[C@H]1O[C@H]1[C@@H]([C@H](C[C@@H](C)O1)OC)O)O)O2	-7.6	*Streptomyces* sp. HK-2006-1
231	**M231**	c1c(c(c2c(c1C(=O)OC)C(=O)c1c([OH+]2)cc(nc1)C)OC(=O)C)OC	-7.6	*Diaporthe phaseolorum* SKS019
232	**M232**	C1(=C[C@H]2[C@](CC1)([C@@]1([C@](O2)(CCC1)CO[C@H]1O[C@@H]([C@H]([C@@H]([C@@H]1O)O)O)CO)C)C)C	-7.6	*Stachybotrys chartarum*
233	**M233**	c1(c(cc2c(c1C)c(c1c(c2)[C@H](OC1=O)[C@]1([C@@H](C(=CC(=O)[C@@H]1OC)OC)O)O)O)OC)C(=O)OC	-7.6	*Saccharothrix* sp. 10-10
234	**M234**	C([C@@H](CC/C(=C/[C@@H]1O[C@H]2[C@@](C1)(OC1=C(C2)C(=O)[C@H](CC1)O)C)/C)O)(C)(C)O	-7.6	*Alternaria alternata* k21-1
235	**M235**	C1(=O)OC(C2=CC(=O)[C@]3([C@H](C(=C2C1)COC(=O)CC(C)C)C[C@@]1([C@@H]2[C@H]3C(=O)[C@H](O[C@@H]2OC1=O)C)C)C)(C)C	-7.6	*Talaromyces amestolkiae* YX1

236	**M236**	[C@@H]1(C([C@H]2[C@](CC1)([C@@]1([C@@H](CC2)C)Oc2c(C1)c(cc1c2CN(C1=O)CC(=O)OC)O)C)(C)C)O	-7.6	*Stachybotrys chartarum* 952
237	**M237**	c1c(cc(c(c1CCC(=O)C[C@@H](C)O)C(=O)O[C@@H](CC(=O)O[C@@H](Cc1c(c(cc(c1)O)O)C(=O)O[C@@H](CC(=O)O[C@@H](Cc1cc(cc(c1)O)O)C)C)C)C)O)O	-7.6	*Hansfordia sinuosae*
238	**M238**	C1[C@@]23[C@@H]([C@H]4/C(=C(\CC[C@H](C)OC(=O)C4)/O)/C(=O)O2)[C@H]([C@@](OC3=O)([C@@H]1C)C)O	-7.6	*Streptomyces koyangensis* SCSIO 5802
239	**M239**	C1[C@@H](C[C@]2([C@](C1)([C@@H]1[C@@H](CC2=O)[C@]2([C@](CC1)([C@H](CC2)[C@@H](/C=C/[C@@H](C(C)C)C)C)C)O)C)O)O	-7.6	*Aspergillus terreus* GX7-3B
240	**M240**	c1cc(c2c(c1O)C(=O)c1c(C2=O)cc(cc1)C)O	-7.6	Mangrove endophytic fungus No.5094
241	**M241**	CC1=CC(=O)C2=C([OH+]1)O[C@]1(CC[C@H]3[C@@](CC[C@H]4C(C)(C)[C@H]([C@H](C[C@]34C)OC(=O)C)OC(=O)C)([C@@H]1C2)C)C	-7.6	Neosartorya laciniosa KUFC 7896
242	**M242**	c1(c(cc(cc1)COc1cc(ccc1[C@@](CCCC(C)C)(C)O)CO)O)[C@@](CCCC(C)C)(C)O	-7.6	*Aspergillus* sp.
243	**M243**	COC(=O)[C@H]1[C@@H](C=CC2=C1C(=O)c1c(cc(cc1[OH+]2)CO)O)O	-7.6	*Neosartorya fischeri* 1008F1
244	**M244**	[C@H]12N(C(=O)[C@H](NC1=O)Cc1ccc(cc1)O)CCC2	-7.6	*Aspergillus versicolor* ZBY-3
245	**M245**	C1(=O)C2=C(C(=C([OH+]1)C)C)O[C@@]1(C2)C(=C[C@H]([C@@H]2[C@]3(C=CC(=O)C([C@@H]3CC[C@@]12C)(C)C)C)O)C	-7.6	*Penicillium* sp.
246	**M246**	C1(=O)C2=C(C(=C([OH+]1)C)C)O[C@@]1(C2)C(=CC[C@@H]2[C@]3(C=CC(=O)C([C@@H]3CC[C@@]12C)(C)C)C)C	-7.6	*Penicillium* sp.
247	**M247**	C12=C(C(=O)[N+]3C(=N1)[C@](NC(=O)[C@@H]3Cc1ccccc1)(C(C)C)OC)C=CC=CO2	-7.6	Paecilomyces variotii EN-291
248	**M248**	C[C@]1([C@@H]([C@H](C2=C([C@H]1O)C(=O)c1c(C2=O)c(cc(c1)OC)O)O)O)O	-7.6	*Stemphylium globuliferum*
249	**M249**	c1c(c(c2c(c1C(=O)OC)C(=O)C1=C([OH+]2)C=C(O[C@@H]1OC)C)O)OC	-7.6	*Chaetomium* sp. strain Gö 100/2
250	**M250**	C[C@@H]1CCC/C=C/C(=O)c2c(cc(cc2O)O)CC(=O)O1	-7.6	*Curvularia* sp. strain no. 768
251	**M251**	C[C@@H]([C@@H](/C=C/C=C/C(=O)O[C@@H]1C=C2COC(=O)[C@@]2([C@@]2(C)CCCC(C)(C)[C@H]12)O)O)O	-7.6	*Aspergillus ustus*
252	**M252**	CC(C)C[C@H]1C(=O)N2CCCC[C@H]2/C(=N/[C@@H](CCCCCC(=O)[C@@H]2CO2)/C(=N/[C@H](Cc2ccccc2)/C(=N\1)/O)/O)/O	-7.6	*Petriella* sp.
253	**M253**	CC(=CC[C@H]1CCC[C@H]2[C@@H](C[C@H]3C(=N[C@@H](C)C(=O)N3O)O)C[NH2+][C@@H]12)C	-7.6	*Aspergillus sydowi* PFW1-13
254	**M254**	c12c(c[nH]c2cccc1)c1c2c([nH]c1)cccc2	-7.5	*Aspergillus* sp.
255	**M255**	C1(=O)/C=C/[C@@H]2[C@@]34C(=C(C(=O)CC[C@H](C)O1)C(=O)O3)O[C@@]([C@H](C4)C)([C@H]2O)C	-7.5	*Streptomyces koyangensis* SCSIO 5802
256	**M256**	C1(=O)[C@H]2N(C(=O)[C@H](NC(=O)[C@H]([C@H](OC(=O)[C@@H](N(C)C(=O)CN1C)C(C)C)C)NC(=O)c1ccc(c3c1Nc1c(O3)c(c3c(c1C(=O)N[C@H]1[C@@H](C)OC(=O)[C@H](C(C)C)N(C(=O)CN(C)C(=O)[C@@H]4CCCN4C(=O)[C@@H](C(C)C)NC1=O)C)nc(o3)CCC(=O)O)C)C)C(C)C)CCC2	-7.5	*Streptomyces* sp. IMB094

257	**M257**	C1(=O)/C=C/[C@@H]2[C@@]34C(=C(C(=O)CC[C@@H]1C)C(=O)O3)O[C@@]([C@H](C4)C)([C@H]2O)C	-7.5	*Streptomyces koyangensis* SCSIO 5802
258	**M258**	C1C[C@H]([C@@H]2[C@H](C1)[C@@H]([C@@H]([NH2+]2)/C=C(/[C@@H](CO)O)\O)N[C@@H]1[C@@H](C(=O)O)CCCC1)C(=O)O	-7.5	*Streptomyces* sp. CMN-62
259	**M259**	C1(=C2[C@]34[C@H](C(=O)[C@H](CC1)C)[C@H]([C@@H]1[C@@]3(OC2=O)C[C@@H]([C@]([C@H]1O)(C)O4)C)S[C@@H]1[C@@H]2[C@@]34[C@]5([C@@H]1[C@@H]([C@@]([C@H](C5)C)(C)O3)O)OC(=O)C4=C(CC[C@@H](C2=O)C)O)O	-7.5	*Streptomyces koyangensis* SCSIO 5802
260	**M260**	c12cccc3c1c(c(=O)[n+]2C)c1c(c3)C(N(C1=O)[C@@H](CC(=O)C)C)(C)C	-7.5	*Penicillium commune* DFFSCS026
261	**M261**	C(C[C@@H](CCCCCC)C)C/C=C(/C=C/C(=O)[C@@H](CC[C@@H]1[C@H](C(=O)NC(=C)C(=O)NC[C@@H](C(=O)N[C@@H](C(=O)O1)CCC(=O)N)C)C)C)\C	-7.5	*Diaporthe phaseolorum* SKS019
262	**M262**	[C@H]1(CC[C@@H]([C@H]2[C@@]1(C=C([C@H]([C@@H]2/C=C(/C(=O)O)\C)/C(=C/C[C@@H](C[C@H](C[C@H]([C@@H]([C@H](C(C)C)O)C)O)O)O)/C)C)C)C)O	-7.5	*Streptomyces* sp. SCSGAA 0027
263	**M263**	[C@@]12([C@H]3[C@](CC[C@@H]1C(OC(=O)C=C2)(C)C)([C@@]12[C@@](C(=O)[C@@](C3)(C)C2=C)(O)[C@@H](C)OC1=O)C)C	-7.5	*Penicillium brasilianum*
264	**M264**	C1(=O)OC(C2=CC(=O)[C@]3([C@H](C(=C2C1)COC(=O)C)C[C@@]1([C@@H]2[C@H]3C(=O)[C@H](O[C@@H]2OC1=O)C)C)C)(C)C	-7.5	*Talaromyces amestolkiae* YX1
265	**M265**	c1(cc(c2c(c1)CC(=O)O[C@H](CCC/C=C/C2=O)C)O)O	-7.5	*Penicillium Sumatrense*
266	**M266**	c1c(cc(c(c1CCC(=O)C)C(=O)O[C@@H](CC(=O)O[C@@H](Cc1c(c(cc(c1)O)O)C(=O)O[C@@H](CC(=O)O[C@@H](Cc1cc(cc(c1)O)O)C)C)C)C)O)O	-7.5	*Hansfordia sinuosae*
267	**M267**	c1c(cc(c(c1CCC(=O)C[C@@H](C)OC)C(=O)O[C@@H](CC(=O)O[C@@H](Cc1c(c(cc(c1)O)O)C(=O)O[C@@H](CC(=O)O[C@@H](Cc1cc(cc(c1)O)O)C)C)C)C)O)O	-7.5	*Hansfordia sinuosae*
268	**M268**	c1c(cc2c(c1O)C(=O)c1c(C2=O)cc(cc1O)O)C	-7.5	*Penicillium* sp. SCSGAF 0023
269	**M269**	C1C[C@](c2c(C1)c1c(c(c2)O)[C@@H]2[C@](CC1)([C@@H](CO2)O)C)(C)COC(=O)C	-7.5	*Aspergillus wentii* SD-310
270	**M270**	c12c([OH+]C(=O)C=C1)c(c1c(c2C)cco1)CC=C(C)C	-7.5	*Penicillium* sp. ZH16
271	**M271**	N(C(=O)[C@@H](N(C(=O)[C@@H](NC(=O)[C@@H]1N(C(=O)[C@H](O)Cc2ccccc2)CCC1)CO)C)[C@H](CC)C)[C@H](C(=O)O)Cc1ccc(cc1)OCC=C(C)C	-7.5	*Bionectria ochroleuca*
272	**M272**	c1c(cc2c(c1C(=O)OC)C(=O)c1c([OH+]2)cc(c(c1)O)C)O	-7.5	*Phomopsis* sp. SK7RN3G1
273	**M273**	CC(=CCC/C(=C\COc1cc2[C@H](C)N(c3ccc(cc3OCC=C(C)C)OC)C(=O)c2c(c1C)OC)/C)C	-7.5	*Stachylidium* sp.
274	**M274**	CC(=CCc1c(C)cc(cc1Oc1cc(C)cc(c1)O)O)C	-7.5	*Aspergillus versicolor*
275	**M275**	c1ccc2c(c1)C(=O)[N+]1c3ccccc3C(=N[C@@H](CCC(=N)O)C1=N2)O	-7.5	*Penicillium aurantiogriseum*
276	**M276**	C[C@@]1(CC2=C(C[C@H]1O)C(=O)c1c(C2=O)cc(cc1O)OC)O	-7.5	*Stemphylium globuliferum*

277	**M277**	C1CC[C@H]2[C@H](C1)[C@H](C[NH2+]2)[C@@H]1[C@H]([C@H]([NH2+][C@@H]1C(=O)OC)C(=O)OC)[C@H]1[C@@H]2[C@H]([NH2+]C1)CC[C@@H](C2)Cl	-7.5	*Marinispora sp.* NPS12745
278	**M278**	c12c(cc(cc1O)C)C(=O)c1c(C2=O)c(ccc1)OC	-7.5	*Nocardia* sp. ALAA 2000
279	**M279**	c1c(c(c2c(c1C(=O)OC)C(=O)C1=C([OH+]2)C=C(OC1)C)O)OC	-7.5	*Chaetomium* sp. strain Gö 100/2
280	**M280**	c1(c(cc2c(c1C(=O)O)C(=O)C1=C([OH+]2)C=C(O[C@@H]1OC)C)O)O	-7.5	*Chaetomium* sp. strain Gö 100/2
281	**M281**	CC1(C)CCC[C@@]2(C)[C@H]1[C@@H](C=C1COC(=O)[C@]21O)OC(=O)/C=C/C=C/C(=O)O	-7.5	*Aspergillus ustus*
282	**M282**	[C@H]1(NC(=O)[C@H]2N(C1=O)C[C@H](C2)O)Cc1ccccc1	-7.4	*Aspergillus* sp.
283	**M283**	c12c3c([nH]c2CCCC1)cncc3	-7.4	*Aspergillus* sp.
284	**M284**	C1C[C@H]([C@@H]2[C@@H]3[C@@H]1N[C@H](C(=O)N[C@@H](C[C@@H]3C[NH2+]2)CO)[C@H](CC)C)C(C)(C)C=C	-7.4	*Marinactinospora thermotolerans* SCSIO 00652
285	**M285**	c12cccc3c1c(c(=O)[nH+]2)c1c(c3)C(N(C1=O)[C@@H](CC(=O)C)C)(C)C	-7.4	*Penicillium commune* DFFSCS026
286	**M286**	c12cccc3c1[C@@](C(=O)N2C)([C@H]1[C@@H](C3)CNC1=O)O	-7.4	*Penicillium commune* DFFSCS026
287	**M287**	c1c(cc2c(c1C(=O)OC)C(=O)c1c([OH+]2)cc(nc1)CO)O	-7.4	*Diaporthe phaseolorum* SKS019
288	**M288**	c1c(cc2c(c1O)C(=O)c1c(C2=O)cc(nc1)C)OC	-7.4	*Diaporthe phaseolorum* SKS019
289	**M289**	C1=C[C@]2([C@H]3[C@]([C@@H]1O)([C@@]1(C(=C[C@H]3OC2=O)COC(=O)C1)O)C)C	-7.4	*Aspergillus wentii* SD-310
290	**M290**	C([C@@H](CC/C(=C/[C@@H]1O[C@H]2[C@@](C1)(OC1=C(C2)C(=O)CC[C@@H]1O)C)/C)O)(C)(C)O	-7.4	*Alternaria alternata* k21-1
291	**M291**	c1(cc(c2c(c1C=O)O[C@](C=C2)(CC/C=C(/C)\CC[C@@H](O)C(C)(O)C)C)O)COC(=O)C	-7.4	*Stachybotrys chartarum* 952
292	**M292**	c1c(cc(c(c1O)C(=O)O)c1cc(c2c(c1C)scn2)O)OC	-7.4	*Alternaria* sp.
293	**M293**	c1c(cc(c(c1C)C(=O)O[C@@H](CC(=O)O[C@@H](Cc1c(c(cc(c1)O)O)C(=O)O[C@@H](CC(=O)O[C@@H](Cc1cc(cc(c1)O)O)C)C)C)C)O)O	-7.4	*Hansfordia sinuosae*
294	**M294**	c1c(cc(c(c1CCC(=O)/C=C/C)C(=O)O[C@@H](CC(=O)O[C@@H](Cc1c(c(cc(c1)O)O)C(=O)O[C@@H](CC(=O)O[C@@H](Cc1c(c(cc(c1)O)O)C(=O)O)C)C)C)C)O)O	-7.4	*Hansfordia sinuosae*
295	**M295**	c1c(cc2c(c1O)C(=O)c1c(C2=O)cc(cc1O)O)CO	-7.4	*Penicillium* sp. SCSGAF 0023
296	**M296**	C/C(=C/CC1=C(C=C(C)[OH+]C1=O)O)/CC/C=C(/C)\CC[C@@H]1C(=C)CC[C@@H](C1(C)C)OC(=O)C	-7.4	*Neosartorya laciniosa* KUFC 7896
297	**M297**	c1(c(cc(cc1)Cc1c(cc(c(c1)C(CCCC(C)C)(C)C)O)CO)O)[C@@](CCCC(C)C)(C)O	-7.4	*Aspergillus* sp.
298	**M298**	[C@H]12N(C(=O)[C@H](NC1=O)Cc1ccccc1)CCC2	-7.4	*Aspergillus versicolor* ZBY-3
299	**M299**	O[C@H]1C=CC[C@@H]2C[C@]34SS[C@@]5(C[C@@H]6[C@@H](O)C=C[C@H](O)[C@H]6N5C3=O)C(=O)N4[C@H]12	-7.4	*Penicillium brocae* MA-231
300	**M300**	c1ccc2c3c1O[C@]14c5c(C(=O)[C@H]([C@H]1OC)Cl)c(ccc5O[C@]3([C@@H](CC2=O)OC)O4)O	-7.4	*Lasiodiplodia theobromae* ZJ-HQ1

301	**M301**	CC(=O)O[C@@H]1[C@H](C2=C([C@H]([C@]1(C)O)O)C(=O)c1c(C2=O)c(cc(c1)OC)O)O	-7.4	*Stemphylium globuliferum*
302	**M302**	C1C([C@@](C(=CC1=O)C)(O)/C=C/C(=C\C(=O)O[C@@H]1[C@H]([C@@H](C=C(C1=O)CO)O)O)/C)(C)C	-7.4	*Nigrospora* sp.
303	**M303**	C[C@H]1[C@H](C)OCc2c1c(C)c(c(c1c(c(C)c3[C@H](C)[C@@H](C)OCc3c1O)O)c2O)O	-7.4	*Aspergillus* sp. MF-93
304	**M304**	c1(ccccc1)C[C@H]1N(C(=O)[C@@H](OC(=O)[C@@H](NC(=O)[C@@H](OC(=O)[C@@H](N(C(=O)[C@H](NC1=O)C)C)C)C(C)C)C)CC(C)C)C	-7.4	*Spicellum roseum*
305	**M305**	c1ccc(cc1)CC1=CC(=O)C(=C[OH+]1)C(=O)N	-7.4	*Aspergillus carbonarius*
306	**M306**	[C@H]1(NC(=O)[C@@H]2N(C1=O)CCC2)Cc1ccc(cc1)O	-7.3	*Aspergillus* sp.
307	**M307**	O=C1N2[C@H](CCC2)C(=O)N[C@@H]1Cc1ccccc1	-7.3	*Aspergillus* sp.
308	**M308**	C1CCC[C@H]2[C@@H]1[C@H](C[N@H+]2CC=C(C)C)C[C@@H](C(=O)OC)NC(=O)[C@H](CC1CCCCC1)O	-7.3	*Aspergillus* sp. SCSIO XWS03F03
309	**M309**	c1c(cc2c(c1C(=O)OC)C(=O)c1c([OH+]2)cc(nc1)C)OC	-7.3	*Diaporthe phaseolorum* SKS019
310	**M310**	c1c(c(c2c(c1C(=O)OC)C(=O)c1c([OH+]2)cc(nc1)C)OC)OC	-7.3	*Diaporthe phaseolorum* SKS019
311	**M311**	C1CC[C@H]2[C@@H](C1)[NH2+][C@@H]([C@H](C2=O)C(=O)O)[C@@H]1CCC[NH2+]1	-7.3	*Penicillium* sp.
312	**M312**	[C@H]1(C[C@H]([C@@H]([C@H]2[C@]1(C=C([C@H]([C@@H]2/C=C(/C(=O)O)\C)/C(=C/C[C@@H](C[C@H](C[C@H]([C@@H]([C@H](C(C)C)O)C)O)O)O)/C)C)C)C)O)O	-7.3	*Streptomyces* sp. SCSGAA 0027
313	**M313**	c1(ccc(cc1)[N](=O)O)C(=O)O[C@@H]1C[C@@H]2[C@@]([C@@]3([C@@H]1[C@@](CCC3)(C)CO)C)(COC2=O)O	-7.3	*Aspergillus ochraceus* Jcma1F17
314	**M314**	C1[C@@H](C([C@H]2[C@](C1)(C1=CC(=C)[C@@]([C@H](C1=CC2)O)(C=C)C)O)(C)C)O	-7.3	*Aspergillus wentii* SD-310
315	**M315**	C(=CCC/C(=C/[C@@H]1O[C@H]2[C@@](C1)(OC1=C(C2)C(=O)CC[C@@H]1O)C)/C)(C)C	-7.3	*Alternaria alternata* k21-1
316	**M316**	[C@@]1([C@H]2[C@](CC[C@@H]1C(O)(C)C)([C@@]13[C@@](C(=O)[C@@](C2)(C)C3=C)(O)[C@@H](C)OC1=O)C)(C)CCC(=O)OC	-7.3	*Penicillium brasilianum*
317	**M317**	c1(cc(c2c(c1)CC(=O)O[C@H](CCCCC2=O)[C@@H](C)O)O)O	-7.3	*Penicillium Sumatrense*
318	**M318**	c1c(cc(c(c1O)C(=O)O[C@@H](CC(=O)O[C@@H](Cc1cc(cc(c1)O)O)C)C)CC(=O)C[C@H](O)C)O	-7.3	*Hansfordia sinuosae*
319	**M319**	c1(cc(c2c(c1)[C@](OC2=O)(c1c(c(ccc1O)CC=C(C)C)O)C)O)C	-7.3	*Ascomycota* sp. SK2YWS-L
320	**M320**	N12C(=O)[C@H](OC(=O)[C@@H](N(C(=O)[C@@H](NC(=O)[C@@H](N(C(=O)[C@@H](NC(=O)[C@@H]1CCC2)CO)C)[C@@H](C)CC)Cc1ccc(cc1)OCC=C(C)C)C)C)Cc1ccccc1	-7.3	*Bionectria ochroleuca*
321	**M321**	C1(=O)[C@@](C(=O)[C@@H]2C(=C1Cl)C[C@@H]([N@@H+](C2)CCCC[N@H+]1C[C@H]2C(=O)[C@@](C(=O)C(=C2C[C@@H]1/C=C/C(=C/[C@@H](CC)C)/C)Cl)(C)OC(=O)C)/C=C/C(=C/[C@H](CC)C)/C)(C)OC(=O)C	-7.3	*Penicillium* sp. 303

322	**M322**	O1C(=C(C(=O)[C@]21C(=O)N[C@@]([C@@H]2O)(OC)C(=O)c1ccccc1)C)[C@H]([C@H](/C=C\CC)O)O	-7.3	*Aspergillus* sp. BRF 030
323	**M323**	c1c(ccc(c1)/C=C(/C(=C/c1ccc(cc1)O)/[N]#C)\[N]#C)O	-7.3	*Penicillium commune* SD-118
324	**M324**	c1(c(cc2c(c1O)C(=O)c1c(C2=O)cc(c(c1O)[C@H](CCCCC)O)O)OC)Cl	-7.3	*Aspergillus* sp. SCSIO F063
325	**M325**	c1ccc2c(c1)C(=C(C(=O)[NH+]2)OC)c1cc(ccc1)O	-7.3	*Aspergillus versicolor* Y31-2
326	**M326**	CC(C)C(=O)Cc1c(C)cc(cc1Oc1cc(C)cc(c1)O)O	-7.3	*Aspergillus versicolor*
327	**M327**	Cc1cc(c2c(c1)[OH+]C1=C(C[C@@H]([C@](C[C@H](C(=O)OC)O)(C(=O)OC)O)S1)C2=O)O	-7.3	*Penicillium oxalicum* SCSGAF 0023
328	**M328**	C1C2=C(C(=O)c3c([OH+]2)cc(cc3O)C)C(=O)C1	-7.3	*Penicillium oxalicum* SCSGAF 0023
329	**M329**	[C@@H]1(CC[C@H]2[C@H](C1)[C@H](C[NH2+]2)[C@@H]1[C@H]([C@H]([NH2+][C@@H]1C(=O)OC)C(=O)OC)[C@H]1[C@@H]2[C@H]([NH2+]C1)CC[C@@H](C2)Cl)Cl	-7.3	*Marinispora*. sp. NPS12745
330	**M330**	c1cc(ccc1)C[C@@H](O[C@@H](CC(C)C)C(=O)O[C@@H]1[C@H]([C@@H](C=C(C1=O)CO)O)O)C(=O)OC	-7.3	*Nigrospora* sp.
331	**M331**	CC1(C)CCC[C@@]2(C)[C@H]1[C@@H](C=C1COC(=O)[C@]21O)OC(=O)/C=C/C=C/C=O	-7.3	*Aspergillus ustus*
332	**M332**	[C@H]1(NC(=O)[C@H]2N(C1=O)CCC2)Cc1ccccc1	-7.2	*Aspergillus* sp.
333	**M333**	C1(=O)N[C@H](C(=O)N2[C@H]1CCC2)Cc1ccccc1	-7.2	*Aspergillus* sp.
334	**M334**	c1(ccccc1)C[C@@H](N(C(=O)[C@@H](NC(=O)C)C(C)C)C)C(=O)N[C@H](C(=O)O)CC(C)C	-7.2	*Simplicillium obclavatum*
335	**M335**	C1(C[C@@H]2/C(=C\[C@@H]([C@@H](C(=C)CC[C@@]12O)NC(=O)/C=C(/CCO)\C)O)/CO)(C)C	-7.2	*Hansfordia sinuosae*
336	**M336**	C1[C@@H](C([C@H]2[C@](C1)(C1=CC(=C)[C@@]([C@@H](C1=CC2)O)(C=C)C)O)(C)C)O	-7.2	*Aspergillus wentii* SD-310
337	**M337**	C(/C=C/C/C(=C/[C@@H]1O[C@H]2[C@@](C1)(OC1=C(C2)C(=O)CC[C@@H]1O)C)/C)(OO)(C)C	-7.2	*Alternaria alternata* k21-1
338	**M338**	[C@H]1([C@@H]([C@@H]2[C@@H](CC1)[C@@]([C@@H](C(=C2)C)/C(=C/C)/C)(/C=C/C=C/C=C/C=C/C(=O)O)C)O)C	-7.2	*Janthinobacterium* spp. ZZ145
339	**M339**	O1[C@H]([C@H]([C@H](C[C@@H]1O[C@@H]1C[C@@](O[C@@H]([C@H]1CC)C)([C@H]([C@@H]([C@@H]([C@@H]1[C@H](/C=C/C=C/C(=C)O[C@@H]([C@H](/C=C/C=C/C(=O)O1)C)[C@H]([C@H]([C@@H]([C@]1(C[C@H]([C@@H]([C@H](O1)C)CC)O[C@H]1C[C@@H]([C@@H]([C@@H](O1)C)O)O)O)C)O)C)C)C)O)C)OC)O)O)C	-7.2	*Streptomyces* sp. 7-145
340	**M340**	c1c(cc(c(c1CCC(=O)C[C@@H](C)OC)C(=O)O[C@@H](CC(=O)O[C@@H](Cc1c(c(cc(c1)O)O)C(=O)O[C@@H](CC(=O)O[C@@H](Cc1c(c(cc(c1)O)O)C(=O)O)C)C)C)C)O)O	-7.2	*Hansfordia sinuosae*

341	**M341**	C([C@@H](C)O)C(=O)Cc1cc(cc(c1C(=O)O[C@@H](CC(=O)O[C@@H](Cc1c(c(cc(c1)O)O)C(=O)O[C@@H](CC(=O)O)C)C)C)O)O	-7.2	*Hansfordia sinuosae*
342	**M342**	C1[C@@](Oc2c(C1=O)c(ccc2c1ccc(c2c1O[C@](CC2=O)(C(=O)OC)[C@@H]1[C@H](CC(=O)O1)C)O)O)([C@]1([C@H](CC(=O)O1)C)C)C(=C)OC	-7.2	*Penicillium* sp. SCSGAF 0023
343	**M343**	c1c(cc2c(c1C(=O)OC)C(=O)c1c(cc(cc1O)CO)[OH+]2)O	-7.2	*Penicillium* sp. ZJ-SY2
344	**M344**	c1ccc2c(c1C(=O)OC)C(=O)c1c(cc(cc1O)C)[OH+]2	-7.2	*Penicillium* sp. ZJ-SY2
345	**M345**	c1ccc2c(c1C(=O)OC)C(=O)c1c(cc(cc1O)CO)[OH+]2	-7.2	*Penicillium* sp. ZJ-SY2
346	**M346**	[C@@]12(C3=CC(=O)OC[C@H]3[C@@H]([C@H]3OC(=O)[C@@](CCC1)([C@@H]23)C)O)C	-7.2	*Aspergillus terreus* GX7-3B
347	**M347**	C(=O)(C(C)C)OC[C@]1([C@H]2[C@@]3(C4=C([C@H]([C@@](CC4)(C)C=C)O)C(=O)[C@@H]2O)[C@@H](C[C@H]1OC(=O)C)C3)C	-7.2	*Eutypella* sp. FS46
348	**M348**	[C@@]123[N+](c4ccccc4[C@@]1(C=C(C(=O)N2/C(=C/c1c[nH]c[nH+]1)/C(=O)N3)O)C(C)(C=C)C)OC	-7.2	*Penicillium commune* SD-118
349	**M349**	c1c(cc2c(c1)C(=O)C1=C(C2=O)[C@H]([C@H]([C@H](O1)C)O)O)O	-7.2	*Acaromyces ingoldii* FS121
350	**M350**	C[C@@]1([C@@H](C2=C(C(=O)c3c(cc(c(c4c(cc(c5c4C(=O)C4=C(C5=O)[C@@H]([C@H]([C@](C)([C@@H]4O)O)O)O)O)OC)c3C2=O)OC)O)[C@@H]([C@H]1O)O)O)O	-7.2	*Stemphylium globuliferum*
351	**M351**	c1c(cccc1C(=O)O[C@@H]1[C@H]([C@@H](C=C(C1=O)CO)O)O)O	-7.2	*Nigrospora* sp.
352	**M352**	CC(C)CCCCCC(=O)N[C@H](Cc1ccc(cc1)O)C(=O)N[C@@H](CO)C(=O)N[C@H](CC(C)C)C(=O)N[C@@H](C[C@@H]1C[NH2+][C@H]2CCCC[C@H]12)C(=O)N[C@@H](CCCN=C(N)N)C(=O)OC	-7.2	*Brevibacillus laterosporus* PNG276
353	**M353**	c1(ccc(cc1)O)C[C@@H]1NC(=O)[C@H](N(C(=O)[C@@H](NC(=O)[C@@H](NC(=O)[C@H]2N(C1=O)CCC2)C(C)C)C(C)C)C)Cc1ccc(cc1)O	-7.2	*Aspergillus versicolor* MST-MF495
354	**M354**	c1c(ccc(c1)C[C@@H](NC(=O)[C@@H](NC(=O)[C@@H](NC(=O)[C@@H](N)C)C(C)C)C(C)C)C(=O)O)O	-7.1	*Aspergillus* sp. SCSIO 41501
355	**M355**	C1CC[C@@H]2[C@H](C1)[C@@H](C[NH2+]2)C[C@@H]1CNC[C@H](N1)C	-7.1	*Serinicoccus profundi* sp.
356	**M356**	C1(=C[C@@]2([C@]3(N(C1=O)[C@H](C(=O)N3)C)[N+](c1c2cccc1)O)C(C)(C)C=C)O	-7.1	*Aspergillus versicolor* LZD-14-1
357	**M357**	C[C@@H]1CCC/C=C/C=C/[C@@H](C[C@H]([C@@H](/C=C\C=C\[C@H](C/C=C/C=C/C(=O)O1)O)O)O)O	-7.1	*Bacillus amyloliquefaciens* SCSIO 00856
358	**M358**	C1=C2[C@@](C(=C)C1=O)([C@H]1[C@@](C2)(CC(C1)(C)C)O)C	-7.1	*Chondrostereum* sp.
359	**M359**	C1(=O)[C@@](C[C@]2(C(=C1)C=C[C@H]([C@@H]2C)OC(=O)/C=C/C(=C/C(C)C)/C)C)(C(=C)C=O)O	-7.1	*Cochliobolus lunatus* SCSIO41401
360	**M360**	c1(cc(c2c(c1)OCc1c2nc(cc1)C)C(=O)O)O	-7.1	*Phomopsis* sp. 33
361	**M361**	c1c(cc(c(c1O)C(=O)O[C@@H](CC(=O)O[C@@H](Cc1cc(cc(c1)O)O)C)C)C[C@H](OC(=O)C[C@H](O)C)C)O	-7.1	*Hansfordia sinuosae*
362	**M362**	[C@@H]1(CC(=C2[C@]([C@@H]1O)(Oc1c(C2=O)c(c(cc1)c1ccc2c(c1O)C(=O)C1=C(C[C@@H]([C@H]([C@@]1(O2)C(=O)OC)O)C)O)O)C(=O)OC)O)C	-7.1	*Penicillium* sp. SCSGAF 0023

363	**M363**	[C@H]1(CC(=C2[C@@]([C@H]1O)(Oc1c(C2=O)c(ccc1c1c(O)c2C(=O)C3=C(O)C[C@@H]([C@H]([C@@]3(Oc2cc1)C(=O)OC)O)C)O)C(=O)OC)O)C	-7.1	*Penicillium* sp. SCSGAF 0023
364	**M364**	c1c(cc(c(c1C(=O)OC)C(=O)c1c(ccc(c1O)CC=C(C)C)O)O)C	-7.1	*Ascomycota* sp. SK2YWS-L
365	**M365**	c1(ccc2c(c1C(=O)OC)C(=O)c1c(cc(cc1O)C)[OH+]2)O	-7.1	*Penicillium* sp. ZJ-SY2
366	**M366**	c1(ccc2c(c1C(=O)OC)C(=O)c1c(cc(cc1O)CO)[OH+]2)O	-7.1	*Penicillium* sp. ZJ-SY2
367	**M367**	CC(=CCC/C(=C\COc1cc2[C@@H](C)N(c3ccc(cc3OCC=C(C)C)OC)C(=O)c2c(c1C)OC)/C)C	-7.1	*Stachylidium* sp.
368	**M368**	C1(=O)[C@@]([C@@H]([C@@H]2C(=C1Cl)C=C(OC2)/C=C/[C@H]([C@@]([C@H]([C@@H](CC)C)O)(O)C)O)O)(O)C	-7.1	*Penicillium sclerotiorum* M-22
369	**M369**	CCCCCC[C@@H](C)C(=O)C1=C([C@@]2([C@H](C)[C@@H]3c4c(cc(c(C)c4[C@H](C)[C@@H](C)O3)O)O2)N(C)C1=O)O	-7.1	*Penicillium citrinum*
370	**M370**	CC1=CC(=O)c2c(cc(c([C@@H]3C[C@H](C(=O)O3)OC)c2[OH+]1)OC)O	-7.1	*Rhytidhysteron rufulum*
371	**M371**	O=C1N[C@H](C(=O)N[C@@H]1Cc1ccccc1)C(C)C	-7.1	*Cladosporium* sp. F14
372	**M372**	c1(ccc(cc1)O)C[C@@H]1NC(=O)[C@H](N(C(=O)[C@@H](NC(=O)[C@@H](NC(=O)[C@H]2N(C1=O)CCC2)C(C)C)[C@@H](C)CC)C)Cc1ccc(cc1)O	-7.1	*Aspergillus versicolor* MST-MF495
373	**M373**	C1(=O)O[C@H](c2c(C1)cc(cc2O)O)CCCCCC[C@@H](C)O	-7.1	*Pestalotiopsis* sp.
374	**M374**	c1(cc(c2c(c1)[OH+]C(=CC2=O)CCCCCCC)CC(=O)O)O	-7.1	*Pestalotiopsis* sp.
375	**M375**	C/C=C\[C@@H]([C@@H](C1=C(C)C(=O)[C@]2([C@H]([C@](C(=O)c3ccccc3)(N=C2O)OC)O)O1)O)O	-7.1	*Aspergillus sydowi* PFW1-13
376	**M376**	C1=C[C@]2([C@H]([C@@H](C1=O)C)[C@@H](C(=O)[C@]1([C@@H]2CC[C@@H]2[C@@]1(C[C@@H](/C/2=C(\CCCC(C)(C)O)/C(=O)O)OC(=O)C)C)C)OC(=O)C)C	-7.1	*Aspergillus sydowi* PFW1-13
377	**M377**	c1(cc2c(cc1C)nc(c(c2O)O)C(=O)O)C	-7	*Aspergillus* sp.
378	**M378**	C1(=O)N[C@H](C(=O)N2[C@H]1CCC2)Cc1ccc(cc1)O	-7	*Aspergillus* sp.
379	**M379**	S1SSC[C@H]2NC(=O)[C@H](NC(=O)C[C@@H](/C=C/CC1)OC(=O)[C@@H](NC(=O)/C(=C/C)/NC2=O)C(C)C)C(C)C	-7	*Chromobacterium* sp. HS-13-94
380	**M380**	c1(ccccc1)C[C@@H](N(C(=O)[C@@H](NC(=O)C)[C@@H](CC)C)C)C(=O)N[C@H](C(=O)O)CC(C)C	-7	*Simplicillium obclavatum* EIODSF 020
381	**M381**	c1ccc2c(c1)[C@]1([C@](N2)(N2[C@@H](C1)C(=O)N[C@H](C2=O)C)C(C)(C=C)C)O	-7	*Eurotium* sp. SCSIO F452
382	**M382**	c1(cc(c(cc1)[C@](CCCC(C)C)(C)O)O)C(=O)O	-7	*Aspergillus* sp.
383	**M383**	[C@@H]1([C@@]([C@@H]2[C@@](C1)(CC(=O)C(=CC2)C)C)(C(C)C)O)O	-7	*Trichoderma virens*
384	**M384**	C1(=O)[C@@](C[C@]2(C(=C1)C=C[C@H]([C@@H]2C)OC(=O)/C=C/C(=C/C(C)C)/C)C)(C(=C)/C(=N\O)/C)OC	-7	*Cochliobolus lunatus* SCSIO41401
385	**M385**	C1=C[C@]2([C@H]3[C@](C1=O)([C@@]1(C(=C[C@H]3OC2=O)COC(=O)C1)O)C)C	-7	*Aspergillus wentii* SD-310
386	**M386**	c1(c(cc2c(c1C)c(c1c(c2)C(=O)[C@]2([C@@](C1=O)(C(=CC(=O)[C@@H]2OC)OC)O)O)O)OC)C(=O)OC	-7	*Saccharothrix* sp. 10-10

387	**M387**	c1(cc(c2c(c1)OCc1c2nc(cc1)C)C(=O)O)OC	-7	*Phomopsis* sp. 33
388	**M388**	c1(cc(c2c(c1O)OCc1c2nc(cc1)C)C(=O)O)OC	-7	*Phomopsis* sp. 33
389	**M389**	O1[C@H]([C@H]([C@H](C[C@@H]1O[C@@H]1C[C@@](O[C@@H]([C@H]1CC)C)([C@H]([C@@H]([C@@H]([C@@H]1[C@H](/C=C/C=C/C(=C)O[C@@H]([C@H](/C=C/C=C/C(=O)O1)C)[C@H]([C@H]([C@@H]([C@]1(C[C@H]([C@@H]([C@H](O1)C)C)O[C@H]1C[C@@H]([C@@H]([C@@H](O1)C)O)O)OC)C)O)C)C)C)O)C)OC)O)O)C	-7	*Streptomyces* sp. 7-145
390	**M390**	[C@H]1([C@@H]([C@@H]2[C@@H](CC1)[C@@]([C@@H](C(=C2)CO)/C(=C/C)/C)(/C=C/C=C/C=C/C=C/C(=O)O)C)O)C	-7	*Janthinobacterium* spp. ZZ145
391	**M391**	c1(cc(c2c(c1)CC(=O)O[C@H](CCCCC2=O)[C@@H](C)O)O)O	-7	*Penicillium Sumatrense*
392	**M392**	c1(cc(c2c(c1)CC(=O)O[C@H](CCCCCC2=O)C)O)O	-7	*Penicillium Sumatrense*
393	**M393**	O1[C@H]([C@H]([C@H](C[C@@H]1O[C@@H]1C[C@@](O[C@@H]([C@H]1CC)C)([C@H]([C@@H]([C@@H]([C@@H]1[C@H](/C=C/C=C/C(=C)O[C@@H]([C@H](/C=C/C=C/C(=O)O1)C)[C@H]([C@H]([C@@H]([C@]1(C[C@H]([C@@H]([C@H](O1)C)CC)O[C@H]1C[C@@H]([C@@H]([C@@H](O1)C)O)O)OC)C)O)C)C)C)O)C)OC)O)O)C	-7	*Streptomyces* sp. 7-145
394	**M394**	C=C1CC[C@H]2[C@@](C)(CC[C@H]3C(C)(C)[C@H](CC[C@]23C)OC(=O)C)[C@@H]1CC1=C(C=C(C)[OH+]C1=O)O	-7	*Neosartorya laciniosa* KUFC 7896
395	**M395**	c1(cc(c2c3c1[C@H](OC(=O)c3c(cc2C)O)C(=O)OC)O)O	-7	*Penicillium purpurogenum* G59
396	**M396**	C1CC(=[NH2])N[C@@H]1C1=Nc2c(C(=O)[N+]1c1c(C(=O)O)cccc1)cccc2	-7	*Penicillium aurantiogriseum*
397	**M397**	COC(=O)c1ccccc1[N+]1C(=Nc2ccccc2C1=O)[C@@H]1CCC(=N)N1	-7	*Penicillium aurantiogriseum*
398	**M398**	c1c(cc2c(c1O)C(=O)C1=C([OH+]2)C=C[C@@H]([C@H]1C(=O)OC)O)CO	-7	*Engyodontium album* DFFSCS021
399	**M399**	c1ccc2c(c1O)C(=O)C1=C(C2=O)[C@H]([C@H]([C@H](O1)C)O)O	-7	*Acaromyces ingoldii* FS121
400	**M400**	c1ccccc1C[C@@H]1N2C(=O)[C@]3(C=CC=COC3=NC2=C(C(C)C)NC1=O)OCC(=O)C(C)C	-7	Paecilomyces *variotii* EN-291
401	**M401**	C[C@@]12CCCCC1=CC=C1C2=CC=CC1=O	-7	*Pseudomonas stutzeri* CMG 1030
402	**M402**	CC(C)CCCCC/C(=N/[C@H](C[C@H]1CC[C@@H](CC1)O)/C(=N/[C@@H](CO)/C(=N/[C@H](CC(C)C)/C(=N/[C@@H](C[C@H]1C[NH2+][C@H]2CCCC[C@@H]12)/C(=N/[C@@H](CCCNC(=N)N)C(=O)O)/O)/O)/O)/O)/O	-7	*Brevibacillus laterosporus* PNG276
403	**M403**	c1cc(ccc1C/C(=N/O)/C(=O)OC[C@@H]([C@@H](COC(=O)/C(=N\O)/Cc1ccc(cc1)O)O)O)O	-7	*Aspergillus aculeatus* CRI323-04A
404	**M404**	c1(cc(c2c(c1)[OH+]C(=CC2=O)CCCCCCC)CC(=O)OC)O	-7	*Pestalotiopsis* sp.
405	**M405**	c1(cc(c2c(c1)[OH+]C(=CC2=O)CCCCC[C@@H](C)O)CC(=O)OC)O	-7	*Pestalotiopsis* sp.
406	**M406**	c1(cc(c2c(c1)[OH+]C(=CC2=O)CCCCC[C@@H](C)O)CC(=O)OCC)O	-7	*Pestalotiopsis* sp.
407	**M407**	CC(=C1C(=O)/C(=C\2/C=CC[C@H](C)O2)/C(=N1)O)C	-6.9	*Cladosporium* sp. OUCMDZ-1635
408	**M408**	c1(ccc(cc1)O)C[C@@H](N(C(=O)[C@@H](NC(=O)C)[C@@H](CC)C)C)C(=O)N[C@H](C(=O)O)CC(C)C	-6.9	*Simplicillium obclavatum* EIODSF 020

409	**M409**	c1(ccc(cc1)O)C[C@@H](N(C(=O)[C@@H](NC(=O)C)[C@@H](CC)C)C)C(=O)N[C@H](C(=O)OC)CC(C)C	-6.9	*Simplicillium obclavatum* EIODSF 020
410	**M410**	c1c(ccc(c1)C[C@@H]1[C@@H](C)N=C(N=C1)Cc1ccc(cc1)OC)OC	-6.9	*Eurotium* sp. SCSIO F452
411	**M411**	C[C@H]1/C=C/C(=O)O[C@H](C)[C@@H](CO[C@H]2[C@@H]([C@@H]([C@@H]([C@@H](C)O2)O)OC)OC)[C@H]2[C@H](/C=C/C(=O)[C@](C)(C[C@H](C)[C@H]1O[C@H]1[C@@H]([C@H](C[C@@H](C)O1)OC)O)O)O2	-6.9	*Streptomyces* sp. HK-2006-1
412	**M412**	C1(=O)[C@@]([C@@H]2[C@@](C1)(CC(=O)C(=CC2)C)C)(C(C)C)O	-6.9	*Trichoderma virens*
413	**M413**	[C@]12(CC[C@]3([C@H]([C@@H]1[C@@H]1[C@]([C@H]4C(=C(CC4)C(=O)O)C2)(C1)C)[C@H](CC3)C(C)C)C)CO	-6.9	*Aspergillus* sp. 16-5c
414	**M414**	c1(cc(c2c(c1)OCc1c2nc(cc1)C)C(=O)OC)O	-6.9	*Phomopsis* sp. 33
415	**M415**	c1(cc(c2c(c1)CC(=O)O[C@H](CCCC2=O)C[C@H](C)O)O)O	-6.9	*Penicillium Sumatrense*
416	**M416**	c1(cc(c2c(c1)CC(=O)O[C@@H](CCCC2=O)C[C@H](C)O)O)O	-6.9	*Penicillium Sumatrense*
417	**M417**	C12=C(C[C@@H]3[C@]4(C(=C1C)C[C@@]1([C@@H]5[C@]4([C@@]([C@@H](O[C@@H]5OC1=O)C)(O3)O)C(=O)OC)C)C)C(OC(=O)C2)(C)C	-6.9	*Penicillium* sp. 303
418	**M418**	c1cccc(c1C(=O)O)N/C=N/c1occc1	-6.9	*Penicillium paneum* SD-44
419	**M419**	C=C[C@H]1CC[C@@]2([C@@H]3CC[C@H](C(C)(C)C3=C(C(=O)C2=C1)O)O)O	-6.9	*Eutypella scoparia* FS26
420	**M420**	CC(C)C[C@H]1[C@@H]2[C@H](C)C(=C[C@@H]3/C=C(/C)\CCCC(=O)C[C@H](C(=O)[C@@]23C(=N1)O)SC)C	-6.9	*Aspergillus* sp.
421	**M421**	c1(ccccc1)CCOC(=O)[C@H]1NC(=O)CC1	-6.9	*Aspergillus versicolor* ZBY-3
422	**M422**	c1c(cc2c(c1O)C(=O)C1=C([OH+]2)C=C[C@H]([C@H]1C(=O)OC)O)CO	-6.9	*Engyodontium album* DFFSCS021
423	**M423**	[C@@H]12[C@@H]([C@]3([C@H]4[C@]([C@@H]1O2)(C1=CC(=C)OCC1=C[C@H]4OC3=O)C)C)O	-6.9	*Aspergillus dimorphicus* SD317
424	**M424**	C1=C[C@]2([C@H]3[C@]([C@@H]1O)(C1=CC(=O)OCC1=C[C@H]3OC2=O)C)C	-6.9	*Aspergillus wentii* EN-48
425	**M425**	CC1(C)[C@@H](C(=O)O)N2C(=O)[C@H]([C@H]2S1)/N=C(/Cc1ccccc1)\O	-6.9	*Penicillium mold*
426	**M426**	C[C@H]1CC(=O)C/C=C/[C@@H]2CCC[C@H]2[C@@H](/C=C/C(=O)O1)O	-6.9	*Penicillium* sp. PSU-F44
427	**M427**	O=C1N2[C@H](C(=O)N[C@H]1Cc1ccccc1)CCC2	-6.9	*Cladosporium* sp. F14
428	**M428**	C[C@@H]1CCC/C=C\[C@]2(CC(=O)c3c(cc(cc3O2)O)CC(=O)O1)O	-6.9	*Curvularia* sp. 768
429	**M429**	c1(cc(c2c(c1)[OH+]C(=CC2=O)CCCCCCC)CC(=O)OCC)O	-6.9	*Pestalotiopsis* sp.
430	**M430**	c1(cc(c2c(c1)[OH+]C(=CC2=O)CCCCC)CC(=O)OC)O	-6.9	*Pestalotiopsis* sp.
431	**M431**	[C@H](CCCCCCCCCCCC)([C@@H]1OC(=O)[C@H](NC(=O)CN(C(=O)/C=C/C(=C)NC(=O)[C@H]1C)C)[C@@H](C(=O)N)O)C	-6.8	*Micromonospora chalcea* FIM 02-523
432	**M432**	C1C(=O)[C@@H]([C@@]2([C@H]1[C@H](C1=C2CC(C1=O)(C)C)O)C)C	-6.8	*Chondrostereum* sp.
433	**M433**	[C@]12([C@@]3(C[C@H](CC1)C([C@@]3(O)C)(C)C)[C@@H](C[C@@H]2C)O)O	-6.8	*Streptomyces* sp. SCSIO 10355
434	**M434**	C1CC([C@]2([C@](C1)(C1=C([C@@H](C2=O)O)[C@H]([C@](CC1)(C=C)C)O)C)O)(C)C	-6.8	*Eutypella scoparia* FS26
435	**M435**	C1CC([C@H]2[C@]3(C1)[C@@]1([C@@](CC2=O)([C@H]([C@](CC1)(C=C)C)O)OC3=O)O)(C)C	-6.8	*Aspergillus wentii* SD-310

436	**M436**	C1CC(C2=CC(=O)C3=C([C@@]2(C1)O)CC[C@@]([C@@H]3O)(C=C)C)(C)C	-6.8	*Aspergillus wentii* SD-310
437	**M437**	C1CC(C2=CC(=O)C3=C([C@]2(C1)O)CC[C@@]([C@@H]3O)(C=C)C)(C)C	-6.8	Aspergillus wentii SD-310
438	**M438**	[C@@]12([C@H]3[C@](CC[C@@H]1[C@@]1(OC(=O)[C@H]([C@H]2O)C1)C)([C@@]12[C@@](C(=O)[C@@](C3)(C)C2=C)(O)[C@@H](C)OC1=O)C)C	-6.8	*Penicillium brasilianum*
439	**M439**	[C@@H]1(CC[C@@H](O[C@@H]1C)O[C@H]1[C@](C[C@@H](O[C@@H]1C)O[C@H]1[C@@H]([C@H](O[C@H](C1)c1c(c2c(cc1)C(=O)C1=C(C2=O)[C@@H]([C@@H]([C@@]2([C@]1([C@H](C=C([C@H]2O)C)O[C@H]1O[C@H]([C@H]([C@@H](C1)OC)O)C)O)O)CC(=O)C)C)O)C)O)(O)C)OC(=O)N	-6.8	*Streptomyces pratensis* NA-ZhouS1
440	**M440**	c1(cc(c2c(c1C=O)O[C@](C=C2)(CC/C=C(/C)\CC[C@@H](O)C(C)(C)OC)C)O)COC(=O)C	-6.8	*Stachybotrys chartarum* 952
441	**M441**	c1cccc(c1C(=O)O)N/C(=N/CC(C)(C)O)/C	-6.8	*Penicillium paneum* SD-44
442	**M442**	Cc1cc(c(C)c(c1)O[C@@H]1C(=O)C(=C(C(=O)[C@@]1(C)OC)O)C)O	-6.8	*Aspergillus aculeatus*
443	**M443**	CC[C@@H](CC(=O)C(Cl)Cl)[C@H](C)c1ccc(cc1)[N](=O)O	-6.8	*Nocardia* sp. ALAA 2000
444	**M444**	C[C@H]1C[C@H](C/C=C/[C@@H]2CCC[C@H]2[C@@H](/C=C/C(=O)O1)O)O	-6.8	*Penicillium* sp. PSU-F44
445	**M445**	C1[C@H](C=C2[C@H]([C@H]1OC(=O)C(CC)(C)C)[C@H]([C@H](C=C2)C)CC[C@@H]1C[C@H](CC(=O)O1)O)C	-6.8	*Aspergillus terreus*
446	**M446**	C1(=C(C=C([OH+]C1=O)/C(=C/[C@@H](CC(=O)O)C)/C)OC)CO	-6.8	*Petriella* sp.
447	**M447**	c1(c(c2c(cc1C)C(=O)C[C@H](O2)/C=C/C)C)O	-6.7	*Aspergillus* sp.
448	**M448**	c1c(c2c(c(c1O)C)[C@H]([C@@H](OC2=O)C)C)O	-6.7	*Aspergillus* sp.
449	**M449**	[C@H](CCCCCCCCCCCCCC)([C@@H]1OC(=O)[C@H](NC(=O)CN(C(=O)/C=C/C(=C)NC(=O)[C@H]1C)C)[C@@H](C(=O)N)O)C	-6.7	*Micromonospora chalcea* FIM 02-523
450	**M450**	[C@H](CCCCCCCCCCCCCCC)([C@@H]1OC(=O)[C@H](NC(=O)CN(C(=O)/C=C/C(=C)NC(=O)[C@H]1C)C)[C@@H](C(=O)N)O)C	-6.7	*Micromonospora chalcea* FIM 02-523
451	**M451**	C(=O)([C@H](CSc1ccc[n+]2c1[C@]([C@H]([C@@H]2/C=C(\C)/C=C/C1CC1)O)(C)O)CC(=O)C)O	-6.7	*Streptomyces* sp. HZP-2216E
452	**M452**	C1(=C[C@@]2([C@]3(N(C1=O)CC(=O)N3)[N@H+]([C@@H]1[C@@H]2C=CC=C1)O)C(C=C)(C)C)OC	-6.7	*Aspergillus versicolor* LZD-14-1
453	**M453**	C1=C2[C@@]([C@H](C1=O)C)([C@]1(C(=C2)C(=O)C(C1)(C)C)O)C	-6.7	*Chondrostereum* sp.
454	**M454**	C1(=O)C=C([C@@H]2[C@]1([C@H]([C@@H]1[C@H]2CC(C1)(C)C)O)C)C	-6.7	*Chondrostereum* sp.
455	**M455**	[C@@H]1([C@@]([C@@H]2[C@@](C1)(CC=C(CC2)C(=O)O)C)(C(C)C)O)O	-6.7	*Trichoderma virens*
456	**M456**	[C@@]12(CC[C@]3([C@H]([C@@H]1CC(=C1[C@@H]([C@H]2O)[C@H](CC1)C)C)CC(CC3)(C)C)C)C	-6.7	*Aspergillus* sp. 085242
457	**M457**	[C@@]12(CC[C@]3([C@H]([C@@H]1CC(=C1[C@@H]([C@H]2O)[C@H]([C@@H](C1)O)C)C)CC(CC3)(C)C)C)C	-6.7	*Aspergillus* sp. 085242
458	**M458**	c1(cc(c2c(c1C=O)O[C@](C=C2)(CC/C=C(/C)\CC[C@@H](O)C(C)(O)C)C)O)C	-6.7	*Stachybotrys chartarum* 952
459	**M459**	c1c(cc(c(c1O)C(=O)O)c1cc(c(cc1C)O)O)OC	-6.7	*Streptomyces olivaceus*
460	**M460**	c1c(cc(cc1O)c1cc(c(cc1C)O)O)OC	-6.7	*Streptomyces olivaceus*

461	**M461**	O1[C@H]([C@H]([C@H](C[C@@H]1O[C@@H]1C[C@@](O[C@@H]([C@H]1CC)C)([C@H]([C@@H]([C@@H]([C@@H]1[C@H](/C=C/C=C/C(=C)O[C@@H]([C@H](/C=C/C=C/C(=O)O1)C)[C@H]([C@H]([C@@H]([C@]1(C[C@H]([C@@H]([C@H](O1)C)C)O[C@H]1C[C@@H]([C@@H]([C@@H](O1)C)O)O)O)C)O)C)C)C)O)C)O)O)O)C	-6.7	*Streptomyces* sp. 7-145
462	**M462**	C1CC(C2=C(C(=O)C3=C[C@@](CC(=O)[C@@]3([C@]2(C1)C)O)(C)C=C)O)(C)C	-6.7	Fungal strain HS-1
463	**M463**	Cc1cc(c2c(c1)[OH+]C1=C(C[C@@H]([C@](CC(=O)OC)(C(=O)OC)O)S1)C2=O)O	-6.7	*Penicillium oxalicum* SCSGAF 0023
464	**M464**	[C@@H]12[C@@H]([C@]3([C@H]4[C@]([C@@H]1O)(C1=CC(=O)OCC1=C[C@H]4OC3=O)C)C)O2	-6.7	*Aspergillus wentii* EN-48
465	**M465**	C1=C[C@]2([C@@H]3[C@]([C@H]1O)(C(=O)O[C@@H]3C=C1C2=CC(=O)OC1)C)C	-6.7	*Aspergillus wentii* EN-48
466	**M466**	[C@]12(C(=O)O[C@@H]3[C@@H]1[C@]([C@@H](CC2)O)([C@H]1[C@H](C3)COC(=O)C1)C)C	-6.7	*Aspergillus wentii* SD-310
467	**M467**	O[C@H]1/C=C\C=C\[C@@H](CCC[C@H](C)OC(=O)/C=C/1)O	-6.7	*Streptomyces lividan*
468	**M468**	O[C@@H]1/C=C\C=C\[C@@H](CCC[C@H](C)OC(=O)/C=C/1)O	-6.7	*Streptomyces lividan*
469	**M469**	CCCCCCCCCCCCCCCc1cc(cc(c1C(=O)Oc1cc(C)c(c(c1)O)C(=O)O)O)O[C@H]1[C@@H]([C@H]([C@H]([C@@H](CO)O1)O)O)O	-6.7	*Cosmospora* sp. SF-5060
470	**M470**	C/C=C(/C)\[C@H]1[C@@H](/C=C/C=C(/C)\[C@H]([C@@H](C)CO)O)[C@@H]2C[C@H]([C@@](C)(C[C@H]2[C@H]2[C@]1(C)O2)O)Cl	-6.7	*Aspergillus* sp.
471	**M471**	C/C=C/[C@@H]1CO[C@]2(C)[C@H]1CC1=C(C=C(C)[OH+]C1=O)O2	-6.7	*Penicillium* sp. PSU-F44
472	**M472**	c1ccc(cc1)CC1=CC(=C(C(=O)[NH+]1)C=O)O	-6.7	*Aspergillus carbonarius*
473	**M473**	C1(=C(C=C([OH+]C1=O)/C(=C/[C@@H](CC(=O)O)C)/C)OC)C	-6.7	*Petriella* sp.
474	**M474**	C1(=O)O[C@H](c2c(C1)cc(cc2O)O)CCCCC[C@@H](C)OC(=O)C	-6.7	*Pestalotiopsis* sp.
475	**M475**	CCOC(=O)[C@H](CCCNC(=N)N)/N=C(/[C@H](C[C@H]1C[NH2+][C@H]2CCCC[C@@H]12)/N=C(/[C@@H](CC(C)C)/N=C(/[C@H](CO)/N=C(/[C@@H](C[C@H]1CC[C@H](CC1)O)/N=C(/CCCCCC(C)C)\O)\O)\O)\O)\O	-6.7	*Brevibacillus laterosporus* PNG276
476	**M476**	C1(=O)O[C@H](c2c(C1)cc(cc2O)O)CCCCCCCCOC(=O)C	-6.7	*Pestalotiopsis* sp.
477	**M477**	[C@H]1([C@@]23N([C@@H]4[C@]1(c1c(N4)cccc1)[C@@]14c5c(N[C@@H]1N1[C@]6([C@H]4O)C(=O)N([C@@](C1=O)(CO)[S@]6=S=S)C)cccc5)C(=O)[C@](N(C2=O)C)(CO)[S@@]3=S=S=S)O	-6.6	*Acrostalagmus luteoalbus* HDN13-530
478	**M478**	c1ccc2c(c1)[C@]1([C@@](N2)(N2[C@@H](C1)C(=O)NCC2=O)C(C)(C=C)C)O	-6.6	*Eurotium* sp. SCSIO F452
479	**M479**	C1(C[C@@H]2/C(=C\[C@@H]([C@@H](C(=C)C[C@@H]([C@@]12O)OC(=O)C)NC(=O)/C=C(/CCO)\C)O)/CO)(C)C	-6.6	*Hansfordia sinuosae*
480	**M480**	c1(ccc(cc1)[C@@](CCCC(C)C)(O)C)CO	-6.6	*Aspergillus* sp.
481	**M481**	C1C(=O)[C@@H]([C@@]2([C@H]1C(=O)C1=C2CC(C1=O)(C)C)C)C	-6.6	*Chondrostereum* sp.
482	**M482**	[C@@H]1([C@@]([C@@H]2[C@@](C1)(CC(=O)C(=CC2)C)C)(C(C)(C)O)O)O	-6.6	*Trichoderma virens*

483	**M483**	C1C[C@@H]([C@@H]2[C@@H](C1=C)[C@@H](CC(=C2)C(=O)O)O)C(C)C	-6.6	*Cochliobolus lunatus* SCSIO41401
484	**M484**	C1CC(C2=C(C(=O)C3=C[C@](CC[C@@]3([C@]2(C1)C)O)(C=C)C)O)(C)C	-6.6	*Eutypella scoparia* FS26
485	**M485**	C1CC([C@]2([C@]3(C1)C1=C([C@H]([C@@]2(OC3)O)O)[C@H]([C@](CC1)(C=C)C)O)O)(C)C	-6.6	*Eutypella scoparia* FS26
486	**M486**	C1[C@@H](C([C@H]2[C@](C1)(C1=C(C(=O)C2)C[C@](C[C@@H]1O)(C=C)C)C)(C)C)O	-6.6	*Aspergillus wentii* SD-310
487	**M487**	C1/C=C/[C@H](C/C=C/[C@H](CC(=O)O[C@@H]1C)O)O	-6.6	*Dendrodochium* sp.
488	**M488**	C1C(=C[C@]2(C1)[C@@H]1[C@H](CC[C@@H]2C)[C@@H](C(=O)O1)C)CO	-6.6	*Penicilliumsp*.FJ-1
489	**M489**	O1C(=O)c2c([C@@H]1CCCCCCC)c(c(c2)O)O)O	-6.6	*Acremonium strictum*
490	**M490**	C=C[C@]1(C)C=C2C(=O)[C@@]3([C@]4(C(C)(C)CCC[C@]4(CO3)[C@]2([C@@H](C1)O)O)O)O	-6.6	*Eutypella scoparia* FS26
491	**M491**	c1c(cc2c(c1O)C(=O)C1=C([OH+]2)CC[C@H]1OC)C	-6.6	The the diethyl sulfate (DES) mutagenesis of *Penicillium purpurogenum* G59
492	**M492**	Cc1cc(c(C)c2c1C(=O)[C@]1(C(=O)C(=C(C(=O)[C@@]1(C)OC)O)C)O2)O	-6.6	*Aspergillus aculeatus*
493	**M493**	c1(cc(c2c3c1COC(=O)c3c(cc2C)O)O)O	-6.6	The the diethyl sulfate (DES) mutagenesis of *Penicillium purpurogenum* G59
494	**M494**	CC1(C)C[C@@H]2[C@H](C1)[C@@H]([C@]1(C)CCC(=C21)C(=O)O)O	-6.6	*Chondrostereum* sp.
495	**M495**	[C@H]1(C[C@]2([C@H]3[C@](C1)(C1=CC(=O)OCC1=C[C@H]3OC2=O)C)C)O	-6.6	*Aspergillus dimorphicus* SD317
496	**M496**	c1c(cc(c2c1C=C(C(=O)[OH+]2)[C@@H]([C@@H](C)O)O)OC)OC	-6.6	*Pestalotiopsis* sp.
497	**M497**	c1c(cc(c2c1C=C(C(=O)[OH+]2)C[C@@H](CO)O)OC)OC	-6.6	*Pestalotiopsis* sp.
498	**M498**	c1c(cc(c2c1C=C(C(=O)[OH+]2)C[C@@H](CO)O)O)OC	-6.6	*Pestalotiopsis* sp.
499	**M499**	c1cc(c(cc1)NC[C@]1([C@H]([C@@H]([C@H](O1)CO)O)O)OC)C(=O)O	-6.5	*Streptomyces* sp. CMN-62
500	**M500**	c1(cc(c(cc1)[C@](CCCC(C)C)(C)O)O)CO	-6.5	*Aspergillus* sp.
501	**M501**	C1[C@@H](C(C2=C(C(=O)C3=C[C@](C[C@H]([C@@]3([C@]2(C1)C)O)O)(C=C)C)O)(C)C)O	-6.5	*Eutypella scoparia* FS26
502	**M502**	C1CC([C@H]2[C@]3(C1)C1=C([C@H]([C@@H]2O)O[C@H]3OC)[C@H]([C@](CC1)(C=C)C)O)(C)C	-6.5	*Aspergillus wentii* SD-310
503	**M503**	[C@@H]1(C([C@H]2[C@](C[C@@H]1O)([C@@]1([C@@H](CC2)C)Oc2c(C1)c(cc(c2C=O)CO)O)C)(C)C)OC(=O)C	-6.5	*Stachybotrys chartarum* 952
504	**M504**	CC(C)CC1=NC=C([C@H](C(C)C)O)[N+](C1=O)O	-6.5	Fungus strain CF07002
505	**M505**	C(=O)([C@@H](/C=C/CCCCCCCCCCC)O)N[C@@H](CO[C@@H]1O[C@@H]([C@H]([C@@H]([C@H]1O)O)O)CO)[C@@H](/C=C/CC/C=C(/CCCCCCCCC)\C)O	-6.5	*Paecilomyces lilacinus* ZBY-1
506	**M506**	CC[C@@H](C)C[C@H](/C=C(/C)\[C@@H]([C@H](C)/C=C(/C)\[C@@H]([C@H](C)/C=C/[C@@H]([C@H](C)/C=C(/C)\C=O)O)O)O)CO	-6.5	*Pestalotiopsis clavispora*
507	**M507**	CCCCCC/C(=C/C(=O)/C=C\1/O[C@@H](C(=O)N1)CCCC)/C	-6.5	*Marinispora* strain NPS008920

508	**M508**	C1(=C(C(=O)[OH+]C(=C1)/C(=C/[C@@H](CCCC(=O)O)C)/C)CO)OC	-6.5	*Petriella* sp.
509	**M509**	C1CC[C@@H]2[C@@H](C1)[C@@H]([C@@H]([NH2+]2)C(C)(C=C)C)C(=O)N	-6.4	*Eurotium* sp. SCSIO F452
510	**M510**	c1c(cc2c(c1O)CN(C2=O)CCCCC(=O)OC)O	-6.4	*Diaporthe phaseolorum* SKS019
511	**M511**	C1(C[C@@H]2/C(=C/C=C\[C@@](C[C@@H]([C@@]12O)OC(=O)C)(C)O)/CO)(C)C	-6.4	*Hansfordia sinuosae*
512	**M512**	c1(cc(c(cc1)[C@@]1(C)OC(CCC1)(C)C)O)CO	-6.4	*Aspergillus* sp.
513	**M513**	[C@@H]1([C@@]([C@@H]2[C@@](C1)(CC(=O)[C@]1([C@@H](C2)O1)C)C)(C(C)C)O)O	-6.4	*Trichoderma virens*
514	**M514**	C1[C@@H](C(C2=C(C(=O)C3=C[C@](CC[C@@]3([C@]2(C1)C)O)(C=C)C)O)(C)C)O	-6.4	*Eutypella scoparia* FS26
515	**M515**	C1=C[C@]2([C@@H](C(C1=O)(C)C)[C@H]([C@@H](C1=C[C@](CC[C@]21O)(C=C)C)O)O)C	-6.4	*Eutypella* sp. FS46
516	**M516**	C1[C@@H](C([C@H]2[C@](C1)(C1=C(C(=O)C2)[C@H]([C@](CC1)(C=C)C)OC(=O)C)C)(C)C)O	-6.4	*Aspergillus wentii* SD-310
517	**M517**	c1c(cc(c(c1O)C(=O)O)c1c2c(c(cc1C)O)ncs2)OC	-6.4	*Alternaria* sp.
518	**M518**	c1c(cc(c(c1C(=O)O)C(=O)c1c(cccc1O)O)O)C	-6.4	*Penicillium* sp. ZJ-SY2
519	**M519**	c1c(cc(c(c1C(=O)OC)C(=O)c1c(cccc1O)O)O)C	-6.4	*Penicillium* sp. ZJ-SY2
520	**M520**	C(=O)(CC[C@@]1([C@H](CCc2c(c(c(cc12)O)C)C=C)C(=C)C)C)OC	-6.4	Endophytic fungus J3
521	**M521**	C=C[C@@]1(C)CC[C@]2(C(=C1)C(=O)C(=C1C(C)(C)CC[C@H]([C@]21C)O)O)O	-6.4	*Eutypella scoparia* FS26
522	**M522**	C(=O)(C)O[C@H]1[C@H]2O[C@H]2C2=CC(=O)[C@]3(C[C@@]2([C@H]1C)C)O[C@]3(C)CO	-6.4	*Penicilliumsp*. PR19 N-1
523	**M523**	C1CC([C@]2([C@]3(C1)[C@@H]1C(=C[C@@](CC1=O)(C)C=C)C(=O)[C@]2(O)OC3)O)(C)C	-6.4	Fungal strain HS-1
524	**M524**	C1CC([C@]2([C@]3(C1)[C@@]1(C(=C[C@@](C[C@H]1O)(C)C=C)C(=O)[C@]2(O)OC3)O)O)(C)C	-6.4	Fungal strain HS-1
525	**M525**	c1(cc(c2c(c1)[OH+]C1=C(C2=O)C[C@H]([C@@]1(O)C(=O)OC)SC[C@@H](C(=O)OC)O)O)C	-6.4	*Penicillium purpurogenum* G59
526	**M526**	C(=O)([C@@H](/C=C/CCCCCCCCCCCCCC)O)N[C@@H](CO[C@@H]1O[C@@H]([C@H]([C@@H]([C@H]1O)O)O)CO)[C@@H](/C=C/CC/C=C(/CCCCCCCCC)\C)O	-6.4	*Paecilomyces lilacinus* ZBY-1
527	**M527**	C(CCCCC/C(=C/C(=O)/C=C\1/O[C@@H](C(=O)N1)CCCCC)/C)C	-6.4	*Marinispora* strain NPS008920
528	**M528**	OC[C@@H]1O[C@H](C[C@H]1O)[N+]1C(=O)NC(=O)C(=C1)C	-6.3	*Aspergillus* sp.
529	**M529**	[C@@H]1(C[C@@H]([C@H]2[C@]([C@@H]1O)(CC[C@](C2)(C(=C)C)O)C)C)O	-6.3	*Penicillium* sp. J-54
530	**M530**	[C@@H]1(C[C@@H]([C@]2([C@]([C@@H]1O)(CC[C@H](C2)C(=C)C)C)O)C)O	-6.3	*Penicillium* sp. J-54
531	**M531**	[C@@H]1([C@@]([C@@H]2[C@@](C1)(C[C@H](C(=C)CC2)O)C)(C(C)C)O)O	-6.3	*Trichoderma virens*
532	**M532**	C(/C=C/C(C)(C)OC)[C@]([C@@H]1CC[C@@]([C@H]1C)(C)O)(O)C	-6.3	*Trichoderma harzianum* X-5
533	**M533**	[C@@H]1([C@@H]([C@](CC1)(C)O)C)[C@](CCC[C@@H](C)C(=O)OC)(C)O	-6.3	*Trichoderma harzianum* X-5
534	**M534**	C1CC(C2=CC(=O)C3=C([C@]2(C1)O)CC[C@@]([C@H]3O)(C=C)C)(C)C	-6.3	*Aspergillus wentii* SD-310

535	**M535**	[C@@]12([C@H]3[C@](CC[C@@H]1[C@@]1(OC(=O)[C@H]([C@H]2O)C1)C)([C@@]1(C(=O)[C@@](C(=O)[C@@](C3)(C)C1=C)(C)O)C(=O)OC)C)C	-6.3	*Penicillium brasilianum*
536	**M536**	C1[C@H]2[C@H]([C@H](C/C=C/[C@H](CC(=O)O[C@@H]1C)O)O)O2	-6.3	*Dendrodochium* sp.
537	**M537**	C1[C@H](C[C@H](C/C=C/[C@H](CC(=O)O[C@@H]1C)O)O)O	-6.3	*Dendrodochium* sp.
538	**M538**	c1(cc(cc(c1)C[C@H](OC(=O)C[C@@H](C)O)C)O)O	-6.3	*Hansfordia sinuosae*
539	**M539**	c1(ccc(cc1)CCO)OC[C@@H](O)C(C)(C)O	-6.3	*Penicilliumsp*.FJ-1
540	**M540**	c1(c(c(cc(c1)O)O)C(=O)CCCCCCC)CC(=O)OC	-6.3	*Dothiorella* sp.
541	**M541**	c1ccc2c(c1)c(=C)[nH+]c([nH]2)[C@@H](C)O	-6.3	*Penicillium commune* SD-118
542	**M542**	CC(C)CC1=CN=C(CC(C)C)C(=O)[N+]1O	-6.3	marine-derived fungus strain CF07002
543	**M543**	c1cc2[C@H](CCC(=O)c2c(c1)O)O	-6.3	*Aspergillus fumigatus*
544	**M544**	O=C1/C=C\[C@@H](OC)/C=C\CCC[C@H](C)OC(=O)/C=C/1	-6.3	*Pestalotiopsis microspore*
545	**M545**	c1c(cc(c2c1C=C(C(=O)[OH+]2)C[C@@H](C)O)OC)OC	-6.3	*Pestalotiopsis* sp.
546	**M546**	C([C@H]1NC(=O)[C@H](NC1=O)Cc1ccc(cc1)O)C(C)C	-6.2	*Aspergillus* sp.
547	**M547**	c1(c(c2c(c(c1O)C)[C@@H]([C@H](OC2)C)C)O)C(=O)O	-6.2	*Aspergillus* sp.
548	**M548**	C1(=O)C[C@H]([C@@H]2[C@@]34C(=C(C(=O)CC[C@@H]1C)C(=O)O3)O[C@@]([C@H](C4)C)([C@H]2O)C)O	-6.2	*Streptomyces koyangensis* SCSIO 5802
549	**M549**	c1(ccc(cc1)O)C[C@@H](N(C(=O)[C@@H](NC(=O)C)[C@@H](CC)C)C)C(=O)O	-6.2	*Simplicillium obclavatum* EIODSF 020
550	**M550**	C1(=C[C@@]2([C@]3(N(C1=O)CC(=O)N3)[N@H+]([C@@H]1[C@@H]2C=CC=C1)OC)C(C=C)(C)C)OC	-6.2	*Aspergillus versicolor* LZD-14-1
551	**M551**	c1c(cc2c(c1O)CN(C2=O)CCO)O	-6.2	*Diaporthe phaseolorum* SKS019
552	**M552**	[C@@]12([C@@H]([C@@]3([C@](C[C@@H]1OC(=O)C)([C@H](C=C3)O)C)CO)CC2(C)C)O	-6.2	*Hansfordia sinuosae*
553	**M553**	[C@@H]1(C[C@@H]([C@H]2[C@]([C@@H]1O)(CC[C@H](C2)C(=C)C)C)C)O	-6.2	*Penicillium* sp. J-54
554	**M554**	[C@@H]12C[C@@]([C@@H]3[C@](C1)([C@H](C[C@H]3O)C)CC=C2C)(C)O	-6.2	*Penicillium bilaiae* MA-267
555	**M555**	C1[C@H]([C@H]2C(C(=C1C)CC/C(=C/[C@@H](C/C(=C/C2)/C)OC)/C)(C)C)O	-6.2	*Trichoderma harzianum* X-5
556	**M556**	C1[C@@H](CC(=O)C/C=C/[C@H](CC(=O)O[C@@H]1C)O)O	-6.2	*Dendrodochium* sp.
557	**M557**	C1/C=C/[C@@H](C/C=C/[C@H](CC(=O)O[C@@H]1C)O)O	-6.2	*Dendrodochium* sp.
558	**M558**	C1=C([OH+]c2c(C1=O)c(cc(c2[C@@H](OC)C[C@H](C(=O)O)OC)OC)O)C	-6.2	*Rhytidhysteron rufulum*
559	**M559**	[C@H]12C(=C[C@H]([C@H](O1)/C=C/C)O)C(=O)O[C@H]2/C=C/C	-6.2	*Acremonium Strictum*
560	**M560**	c1cccc(c1C(=O)O)/N=C(/C(=O)C)\C	-6.2	*Penicillium paneum* SD-44
561	**M561**	C[C@H]1C[C@@H]([C@@H]2[C@@](C)(C(=O)CCO)[C@@](C)(C=C[C@]2(C1)O)O)O	-6.2	*Aspergillus sulphureus* KMM 4640
562	**M562**	Br/C=C/C#CCCCCCC/C=C/C#CCCCCCCCC(=O)N[C@@H](CCl)N	-6.2	*Xestospongia* sp.
563	**M563**	C1[C@H]([C@@H](C[C@@]2([C@H](C[C@H](C=C12)O)C)C)C(=C)C)O	-6.2	*Penicilliumsp*. PR19 N-1
564	**M564**	C1=C([OH+]c2c(C1=O)c(cc(c2[C@@H](OC)C[C@H](C(=O)OC)OC)OC)O)C	-6.2	*Rhytidhysteron rufulum*
565	**M565**	CN1/C(=C/c2ccccc2)/C(=O)N(C)[C@@](CO)(C1=O)SC	-6.2	Pleosporales strain CRIF2
566	**M566**	c1(c(c(cc(c1)O)O)C(=O)CCCCCC[C@@H](C)O)CC(=O)OC	-6.2	*Pestalotiopsis* sp.

567	**M567**	c1(c(c(cc(c1)O)O)C(=O)CCCCCCCC)CC(=O)OC	-6.2	*Pestalotiopsis* sp.
568	**M568**	C1(=O)N[C@@H](C(=O)N2[C@H]1CCC2)[C@@H](CC)C	-6.1	*Aspergillus* sp.
569	**M569**	c1(C)c(c(C(=O)O)c(O)cc1[C@H]([C@H](O)C)C)O	-6.1	*Aspergillus* sp.
570	**M570**	C1(=O)[C@@H]([C@H](C)CC)NC(=O)[C@@H](NC(=O)[C@H](NC(=O)[C@@H](NC(=O)[C@H](NC(=O)[C@H](NC(=O)C[C@H](OC(=O)[C@H](N1)[C@H](CC)C)CCCCCCCCCCCCCC)CCC(=O)OC)CC(C)C)CC(C)C)CC(C)C)CC(=O)OC	-6.1	*Bacillus* sp. 176
571	**M571**	[C@@H]1([C@@]([C@@H]2[C@@](C1)(CC=C(CC2)C)C)(C(C)(C)O)O)O	-6.1	*Trichoderma virens*
572	**M572**	[C@@H]12C[C@@]([C@@H]3[C@](C1)([C@H](C[C@H]3O)C)CC=C2C)(C)OC(=O)C	-6.1	*Penicillium bilaiae* MA-267
573	**M573**	[C@]123[C@H]([C@]4([C@@H]([C@@H](CC1)C)C(=O)C4)C)C[C@H]([C@@H](C[C@H]2C)O)C3(C)C	-6.1	*Trichoderma harzianum* X-5
574	**M574**	[C@H]1([C@@H](CO[C@@H]([C@H]1O)CO)NC(=O)C)O	-6.1	*Virgibacillus dokdonensis* MCCC 1A00493
575	**M575**	C1[C@H](CC(=O)C/C=C/[C@H](CC(=O)O[C@@H]1C)O)O	-6.1	*Dendrodochium* sp.
576	**M576**	C1CC[C@@H](C/C=C/[C@H](CC(=O)O[C@@H]1C)O)O	-6.1	*Dendrodochium* sp.
577	**M577**	C1C[C@]([C@H]2[C@](C1)([C@H](C(=C)[C@H](C2)O)CC(=O)O)C)(C)C(=O)O	-6.1	*Aspergillus terreus* GX7-3B
578	**M578**	O1C(=O)/C=C\[C@](/C=C\[C@@H](/C(=C\CC[C@@H]([C@H]1C)C)/C)OC)(C)O	-6.1	*Eutypella* sp. FS46
579	**M579**	C(=O)(C)O[C@@H]1[C@]23[C@@H]([C@@H]([C@H]([C@@H](/C=C(/C1)\C)O)O)[C@@H](O3)O)CC2(C)C	-6.1	*Ascotricha* sp. ZJ-M-5
580	**M580**	C1(=O)C(=C(C=C([OH+]1)C[C@H](C)O)O)C	-6.1	*Aspergillus* sp. 16-02-1
581	**M581**	C(=O)([C@H](N(C)C(=O)[C@@H](NC(=O)C)[C@@H](CC)C)Cc1ccccc1)O	-6.1	*Simplicillium obclavatum* EIODSF 020e
582	**M582**	O[C@H]1/C=C\[C@@H](OC)/C=C\CCC[C@H](C)OC(=O)/C=C/1	-6.1	*Pestalotiopsis microspore*
583	**M583**	c1cc(c2c(c1)C(=O)C(=C([OH+]2)CO)C)OC	-6.1	*Penicillium* sp.
584	**M584**	C(CCCCC/C(=C/C(=O)CC(=N)O[C@H](C(=O)O)CCCC)/C)C	-6.1	*Marinispora* strain NPS008920
585	**M585**	C[C@H](CC[C@H]1c2cccc(c2C(=O)O1)OC)O	-6.1	*Pleosporales* strain CRIF2
586	**M586**	[C@@H]1(CC([C@H]2[C@](C1)([C@]([C@@H](CC2=O)C)(CO)O)C)(C)C)O	-6.1	*Aspergillus ustus*
587	**M587**	c1c(cc(c2c1C=C(C(=O)[OH+]2)CO)OC)O	-6.1	*Pestalotiopsis* sp.
588	**M588**	[C@H]1(NC(=O)[C@H]2N(C1=O)CCC2)CC(C)C	-6	*Aspergillus* sp.
589	**M589**	C1(=O)N[C@H](C(=O)N2[C@H]1CCC2)CC(C)C	-6	*Aspergillus* sp.
590	**M590**	C1(=O)[C@@H]([C@H](C)CC)NC(=O)[C@@H](NC(=O)[C@H](NC(=O)[C@@H](NC(=O)[C@H](NC(=O)[C@H](NC(=O)C[C@H](OC(=O)[C@H](N1)[C@H](CC)C)CCCCCCCCCCCCCCC)CCC(=O)OC)CC(C)C)CC(C)C)CC(C)C)CC(=O)OC	-6	*Bacillus* sp. 176
591	**M591**	c1(occc1)c1cncc(n1)C[C@@H]([C@H](CO)O)O	-6	*Jishengella endophytica* 161111
592	**M592**	[C@@H]1([C@@]([C@@H]2[C@@](C1)([C@@H](C=C(CC2)C)O)C)(C(C)C)O)O	-6	*Trichoderma virens*
593	**M593**	C1=CC2=CC(=O)[C@@](C[C@@]2([C@H](C1)C)C)(C(=C)CO)O	-6	*Cochliobolus lunatus* SCSIO41401
594	**M594**	C(CC=C(C)C)[C@]([C@@H]1[C@H](C[C@@]([C@H]1C)(C)O)O)(O)C	-6	*Trichoderma harzianum* X-5

595	**M595**	C1CCC(=O)C/C=C/[C@H](CC(=O)O[C@@H]1C)O	-6	*Dendrodochium* sp.
596	**M596**	C1CC[C@H](C/C=C/[C@H](CC(=O)O[C@@H]1C)O)O	-6	*Dendrodochium* sp.
597	**M597**	C1CC[C@H](CCC[C@H](CC(=O)O[C@@H]1C)O)O	-6	*Dendrodochium* sp.
598	**M598**	O1C(=O)[C@@H](N(C(=O)[C@H](OC(=O)[C@@H](N(C(=O)[C@H](OC(=O)[C@@H](N(C(=O)[C@H]1C(C)C)C)Cc1ccccc1)C(C)C)C)Cc1ccccc1)C(C)C)C)Cc1ccccc1	-6	*Fusarium* sp. DZ27
599	**M599**	c1(c(cc(c1)O)O)C(=O)CCCCCCCC)CC(=O)OCC	-6	*Dothiorella* sp.
600	**M600**	[C@@H]12N(C(=O)[C@@H](NC1=O)CC(C)C)CCC2	-6	*Aspergillus versicolor* ZBY-3
601	**M601**	C1=C(C2=CC(=O)C(=C[C@@]2([C@H]([C@H]1OC(=O)C)C)C)O)Cl	-6	*Penicillium* sp. PR19N-1
602	**M602**	C(=O)(c1c(cc(cc1CCCCCCCCC)O)O)O	-6	*Lasiodiplodia* sp. 318
603	**M603**	CCCC[C@@H](CC)COC(=O)c1ccccc1C(=O)OC[C@H](CC)CCCC	-6	*Cladosporium* sp. F14
604	**M604**	[C@H]1(NC(=O)[C@H]2N(C1=O)C[C@@H](C2)O)CC(C)C	-5.9	*Aspergillus* sp.
605	**M605**	c1c(c(ccc1)C(=O)OCC(C)C)C(=O)OCCCC	-5.9	*Aspergillus* sp.
606	**M606**	C1(=O)[C@]([C@H](C(=C1C)[C@@H](CC/C=C/C)O)O)(O)C	-5.9	*Trichoderma* sp. HPQJ-34
607	**M607**	C1(=O)C2=C[C@@H]3[C@@]45[C@@]2(C(=C(CC[C@@H]1C)O)C(=O)O4)O[C@@]([C@H](C5)C)([C@H]3O)C	-5.9	*Streptomyces koyangensis* SCSIO 5802
608	**M608**	C1(C[C@@H]2/C(=C\[C@@H](/C=C(/C[C@@H]([C@@]12O)OC(=O)C)\C)O)/CO)(C)C	-5.9	*Hansfordia sinuosae*
609	**M609**	[C@@H]1(C[C@@H]([C@]2([C@]([C@@H]1O)(CC[C@](C2)(C(=C)C)O)C)O)C)O	-5.9	*Penicillium* sp. J-54
610	**M610**	C1(=C[C@H]2[C@](CC1)([C@@]1([C@](O2)([C@H](C[C@H]1O)OC)CO)C)C)C	-5.9	*Stachybotrys chartarum*
611	**M611**	[C@]123[C@H]([C@H](C(=O)C4=C1CC[C@@]([C@@H]4O)(C=C)C)O)[C@@]([C@@H](C[C@H]2C3)OC(=O)C)(CC(=O)C(C)C)C	-5.9	*Eutypella* sp. FS46
612	**M612**	C1/C=C/C(=O)C[C@H](C[C@H](CC(=O)O[C@@H]1C)O)OC	-5.9	*Dendrodochium* sp.
613	**M613**	C(=O)([C@@H](N(c1c(cccc1)C(=O)O)C(=O)[C@H](N(C)C(=O)[C@@H](N)C(C)C)Cc1ccccc1)CC(C)C)N	-5.9	*Simplicillium obclavatum* EIODSF 020e
614	**M614**	C(=O)(c1c(cc(cc1CCCCCCC[C@@H](C)O)O)O)OCC	-5.9	*Lasiodiplodia* sp. 318
615	**M615**	[C@@H]12[C@@H](C(=O)[C@@H](NC1=O)CC(=C)C)CCC2	-5.9	*Penicillium purpurogenum* G59
616	**M616**	C(=O)(C)O[C@@H]1[C@]23[C@@H]([C@@H]([C@H]([C@@H](/C=C(\C1)/C)O)OC)[C@@H](O3)O)CC2(C)C	-5.9	*Ascotricha* sp. ZJ-M-5
617	**M617**	[C@@H]12N(C(=O)[C@@H](NC1=O)[C@@H](CC)C)CCC2	-5.9	*Aspergillus versicolor* ZBY-3
618	**M618**	C(=O)([C@@H](CCCCCCCCCCCCCC)O)N[C@@H](CO[C@@H]1O[C@@H]([C@H]([C@@H]([C@H]1O)O)O)CO)[C@@H](/C=C/CC/C=C(/CCCCCCCCC)\C)O	-5.9	*Paecilomyces lilacinus* ZBY-1
619	**M619**	C(=O)([C@@H](CCCCCCCCCCCCCCCC)O)N[C@@H](CO[C@@H]1O[C@@H]([C@H]([C@@H]([C@H]1O)O)O)CO)[C@@H](/C=C/CC/C=C(/CCCCCCCCC)\C)O	-5.9	*Paecilomyces lilacinus* ZBY-1
620	**M620**	C1=C([OH+]c2c(C1=O)c(cc(c2[C@H](OC)C[C@H](C(=O)OC)OC)OC)O)C	-5.9	*Rhytidhysteron rufulum*
621	**M621**	CCCC[C@H]1c2cccc(c2C(=O)O1)OC	-5.9	Fungus strain CRIF2
622	**M622**	C1(=O)N[C@H](C(=O)N2[C@H]1CCC2)C(C)C	-5.8	*Aspergillus* sp.

623	**M623**	C(C)[C@@H](C)[C@@H]1NC(=O)[C@H](NC1=O)CC(C)C	-5.8	*Aspergillus* sp.
624	**M624**	C1(=O)[C@@H](NC(=O)[C@H]2N1CCC2)CC(C)C	-5.8	*Aspergillus* sp.
625	**M625**	c1(cc(c2c(c1OC)C(=O)OC2)COC)C	-5.8	*Penicillium* sp. ZH58
626	**M626**	[C@H]1([C@H](OC(=O)C(=C1)[C@H]([C@H](C)O)Cl)C)O	-5.8	*Exophiala* sp.
627	**M627**	Cc1ccc(cc1C(=O)[C@H](C)O)O	-5.8	*Massarina* sp. CNT-016
628	**M628**	C1[C@@H](C([C@H]2[C@](C1)([C@H]([C@@H](CC2=O)C)CO)C)(C)C)O	-5.8	*Aspergillus ustus*
629	**M629**	C1(=C[C@]2([C@](CC1)([C@@]1(C[C@H](O2)C[C@H]1O)C)C)OCO)C	-5.7	*Stachybotrys chartarum*
630	**M630**	C1C=C(CC[C@]21[C@@H](C[C@H]([C@@H]2C)O)C(C)(C)O)C	-5.7	*Trichoderma harzianum* X-5
631	**M631**	[C@H](C(=C)C(=O)O)(C(=O)O)CCCCC(=O)C	-5.7	*Aspergillus niger*
632	**M632**	[C@H]1([C@H](C=C(C(=O)O1)[C@@H]([C@@H](C)O)OC)O)C	-5.7	*Aspergillus* sp. 16-02-1
633	**M633**	CC[C@@H](C)[C@H](NC(=O)C)C(=O)N(C)[C@@H](Cc1ccc(O)cc1)C(=O)O	-5.7	*Simplicillium obclavatum* EIODSF 020e
634	**M634**	C[C@@H]1[C@H]2CC(=O)[C@@H]([C@@]31[C@H](C(=C)OC3=O)O2)O	-5.7	*Massarina* sp. CNT-016
635	**M635**	C1[C@@H](C([C@H]2[C@](C1)([C@]([C@@H](CC2=O)C)(CO)O)C)(C)C)O	-5.7	*Aspergillus ustus*
636	**M636**	CC(=C1[C@H](C(=O)N(CCC(=O)OC)C1=O)O)C	-5.7	*Pestalotiopsis* sp.
637	**M637**	C(CCCCC/C(=C/C(=O)/C=C\1/O[C@@H](C(=O)N1)CCCC)/C)C	-5.7	*Marinispora* strain NPS008920
638	**M638**	c1c2c(ccc1)c(c[nH]2)CC(=O)O	-5.6	*Aspergillus* sp.
639	**M639**	[C@H]1(OC(=O)C(=C1)[C@H]([C@H](C)O)O)[C@H](C)O	-5.6	*Aspergillus* sp. 16-02-1
640	**M640**	[C@H]1([C@H](OC(=O)C(=C1)[C@@H]([C@@H](C)O)OC)C)O	-5.6	*Aspergillus* sp. 16-02-1
641	**M641**	[C@H](/C=C(/C=C/C(=C)C)\CO)(C)O	-5.6	*Aspergillus* sp. 16-02-1
642	**M642**	C1CCC[C@H]2[C@H]1[NH2+]C[C@@H]2C=O	-5.6	*Jishengella endophytica* 161111
643	**M643**	O1[C@H](C=C(C1=O)[C@H]([C@H](C)O)O)[C@H](C)O	-5.6	*Aspergillus* sp. 16-02-1
644	**M644**	C1(=O)N[C@H](C(=O)N2[C@H]1CCC2)C	-5.5	*Aspergillus* sp.
645	**M645**	[C@@H]1(O[C@H]([C@@H]([C@H]([C@H]1O)O)O)OC(C)C)CO	-5.5	*Aspergillus* sp.
646	**M646**	S([C@]12N([C@H]3C(=CC=C[C@@H]3O)C1)C(=O)[C@@](SC)(N(C2=O)C)CO)C	-5.5	*Aspergillus* sp.
647	**M647**	[C@H]1(OC(=O)C(=C1)[C@@H]([C@@H](C)O)O)[C@H](C)O	-5.5	*Aspergillus* sp. 16-02-1
648	**M648**	[C@H]1(OC(=O)C(=C1)[C@@H]([C@@H](C)O)OC)[C@H](C)O	-5.5	*Aspergillus* sp. 16-02-1
649	**M649**	[C@@H]1(OC(=O)/C(=C/[C@H](C)O)/[C@H]1O)[C@@H](C)O	-5.5	*Aspergillus ochraceus* MA-15
650	**M650**	[C@H]1([C@H](OC(=O)C(=C1)CC(=O)C)C)C	-5.5	*Aspergillus ochraceus* MA-15
651	**M651**	[C@@]12([C@@H]([C@@]3([C@](C[C@@H]1OC(=O)C)(C=C[C@H]3O)C)CO)CC2(C)C)O	-5.5	*Hansfordia sinuosae*
652	**M652**	O1[C@H](C=C(C1=O)[C@@H]([C@@H](C)O)O)[C@H](C)O	-5.5	*Aspergillus* sp. 16-02-1
653	**M653**	O1[C@H](C=C(C1=O)[C@@H]([C@@H](C)O)OC)[C@H](C)O	-5.5	*Aspergillus* sp. 16-02-1
654	**M654**	[C@H]1([C@H](C=C(C(=O)O1)C[C@H](C)O)O)C	-5.5	*Aspergillus* sp. 16-02-1
655	**M655**	[C@H]1([C@H](OC(=O)C(=C1)[C@@H]([C@H](C)Cl)O)C)O	-5.5	*Aspergillus versicolor* ZBY-3
656	**M656**	c1ccc(cc1)/C=C/C(=O)O	-5.5	*Penicillium* sp. PR19N-1
657	**M657**	c1c2[nH]cc(c2ccc1)CCO	-5.4	*Aspergillus* sp.
658	**M658**	[C@H]1(OC(=O)C(=C1)C[C@@H](C)O)[C@H](C)O	-5.4	*Aspergillus* sp. 16-02-1

659	**M659**	[C@H]1(OC(=O)C(=C1)C[C@H](C)O)[C@H](C)O	-5.4	*Aspergillus* sp. 16-02-1
660	**M660**	[C@H]1(OC(=O)C(=C1)CC(=O)[C@H](C)O)C	-5.4	*Aspergillus ochraceus* MA-15
661	**M661**	[C@H](C(=C)C(=O)O)(C(=O)OC)CCCCCCO	-5.4	*Aspergillus niger*
662	**M662**	[C@H](C(=C)C(=O)O)(C(=O)O)CCCCCCOC(=O)C	-5.4	*Aspergillus niger*
663	**M663**	O1[C@H](C=C(C1=O)C[C@@H](O)C)[C@H](C)O	-5.4	*Aspergillus* sp. 16-02-1
664	**M664**	c1c2c(ccc1)c(c[nH]2)C=O	-5.3	*Aspergillus* sp.
665	**M665**	C[C@@H]([C@H]1C=C(C(=O)O1)CC(=O)C)O	-5.3	*Aspergillus* sp. 16-02-1
666	**M666**	O1[C@H](C=C(C1=O)CC(=O)C)[C@H](C)O	-5.3	*Aspergillus* sp. 16-02-1
667	**M667**	O1[C@H](C=C(C1=O)C[C@H](O)C)[C@H](C)O	-5.3	*Aspergillus* sp. 16-02-1
668	**M668**	CC(=O)/C=C/C(=C/[C@@H](C)O)/CO	-5.3	*Aspergillus* sp. 16-02-1
669	**M669**	O[C@@H]1[C@@H](O)[C@@H]([C@H](O[C@H]1CO)OCCC)O	-5.2	*Aspergillus* sp.
670	**M670**	C1(OC(=O)C(=C1)CC[C@H](C)O)(C)C	-5.2	*Aspergillus* sp. 16-02-1
671	**M671**	C1[C@@H](CC(=O)C/C=C/[C@H](CC(=O)O[C@@H]1C)O)OC	-5.2	*Dendrodochium* sp.
672	**M672**	O1[C@H](C=C(C1=O)CC[C@H](C)O)C	-5.2	*Aspergillus* sp. 16-02-1
673	**M673**	C[C@@H](/C=C(/C=C/[C@@H](C)O)\CO)O	-5.2	*Aspergillus* sp. 16-02-1
674	**M674**	C[C@@H](C1=CC(=CC(=O)[OH+]1)OC)O	-5.2	*Nigrospora* sp.
675	**M675**	N12[C@H](C(=O)NCC1=O)CCC2	-5.1	*Aspergillus* sp.
676	**M676**	c1cc(ccc1)CCC(=O)O	-5.1	*Aspergillus* sp.
677	**M677**	C[C@@H](/C=C(/C=C/[C@H](C)O)\CO)O	-5.1	*Aspergillus* sp. 16-02-1
678	**M678**	c1cc(c(cc1)O)CC=O	-4.8	*Aspergillus* sp.
679	**M679**	C1=C(CO)C(=O)[C@H]2[C@@H]([C@@H]1O)O2	-4.8	*Nigrospora* sp.
680	**M680**	c1cc(ccc1)[C@H](CO)O	-4.7	*Aspergillus* sp.
681	**M681**	c1(c(cccc1)O)CN	-4.7	*Aspergillus* sp.
682	**M682**	c1ccc(cc1)C(=O)O	-4.6	*Aspergillus* sp.
683	**M683**	N1[C@@H](CCC1=O)[C@H](OC)O	-4.6	*Aspergillus* sp.
684	**M684**	C(CO)C(=O)[C@@H]1CC(=O)OC1	-4.5	*Penicillium* sp. PSU-F44
685	**M685**	n1cnc(c2c1nc[nH]2)N	-4.4	*Aspergillus* sp.
686	**M686**	[nH]1c(=O)[nH]c(=O)c(c1)C	-4.4	*Aspergillus* sp.
687	**M687**	c1(ccc(o1)CO)C=O	-4.4	*Penicillium chrysogenum* HGQ6
688	**M688**	O1[C@H](C=C(C1=O)CCO)C	-4.4	*Aspergillus* sp. 16-02-1
689	**M689**	c1c(=O)[nH]c(=O)[nH]c1	-3.9	*Aspergillus* sp.
690	**M690**	[C@H]([C@@H](O)C)(O)C	-3.1	*Aspergillus* sp.

Table S2. Docked and MD-refined structures of SARS-CoV-2 Mpro + top-lead ligands.

N0	Name	Docked structure	MD-refined structure
1	**M15**	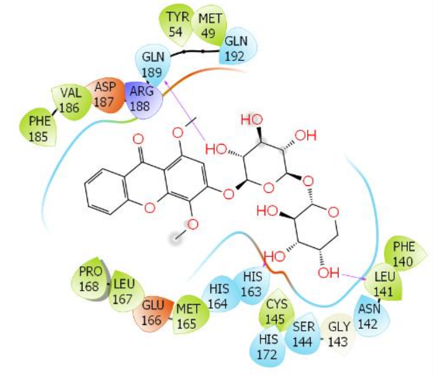	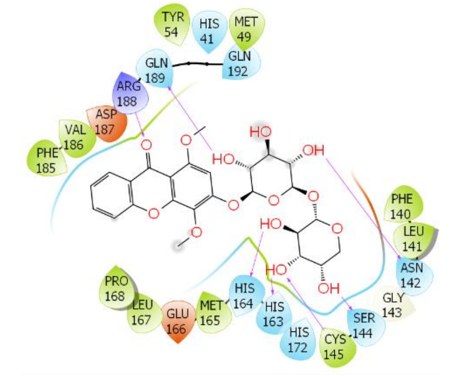
2	**M8**	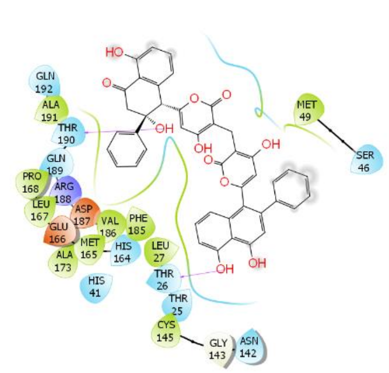	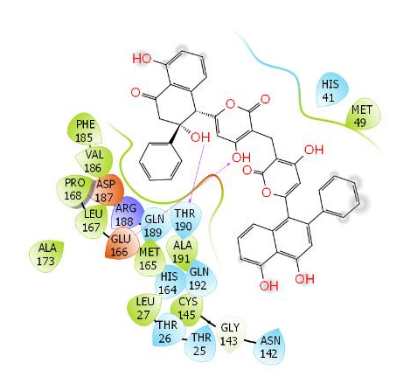
3	**M11**	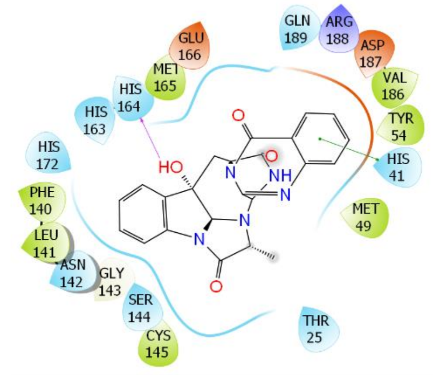	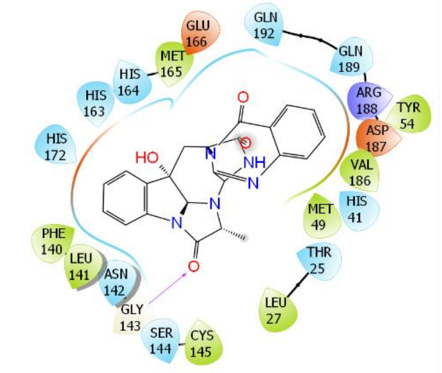
4	**M9**	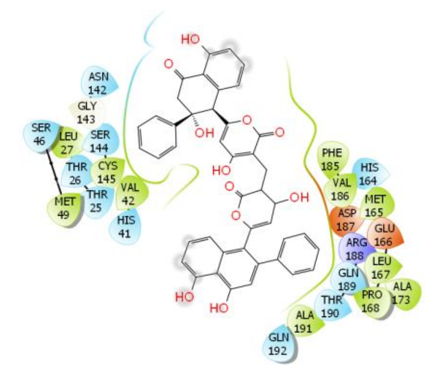	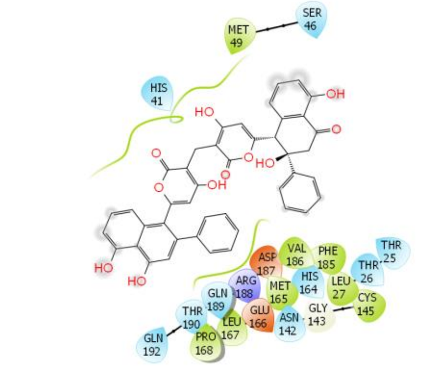

<table>
<tr><td>5</td><td>M2</td><td>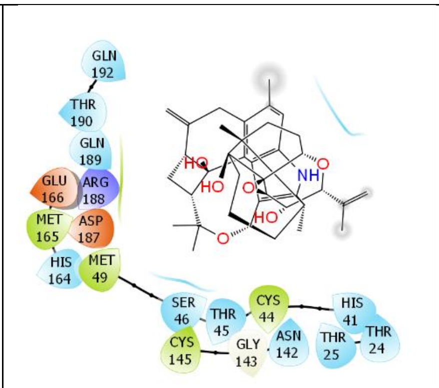
</td><td>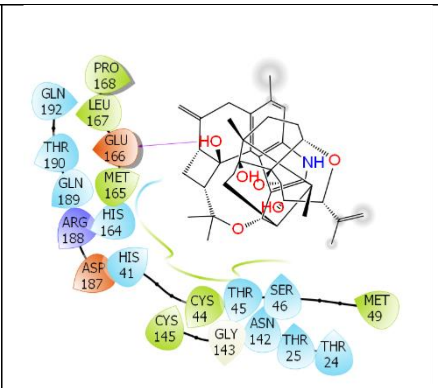
</td></tr>
<tr><td>6</td><td>M13</td><td>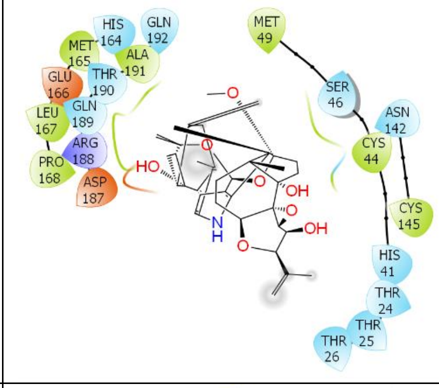
</td><td>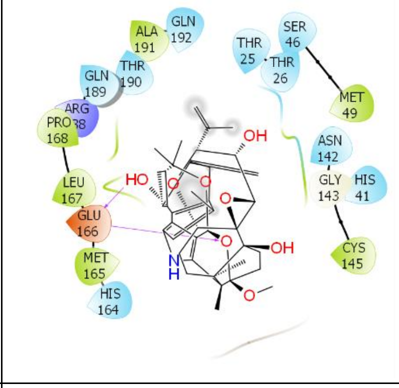
</td></tr>
<tr><td>7</td><td>M3</td><td>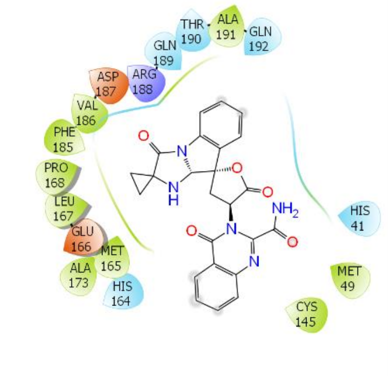
</td><td>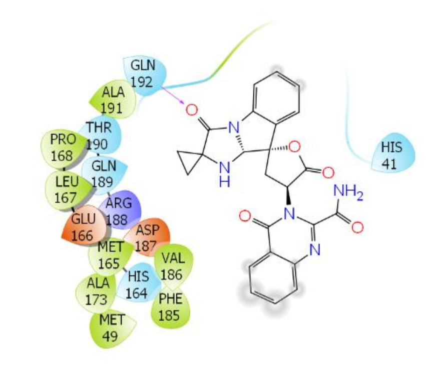
</td></tr>
<tr><td>8</td><td>M5</td><td>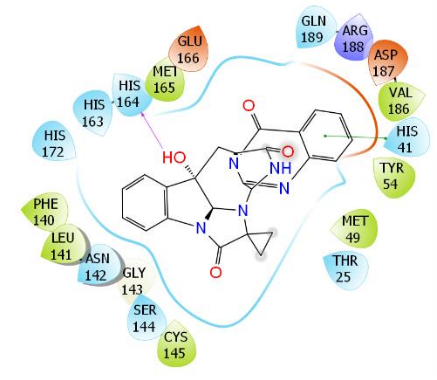
</td><td>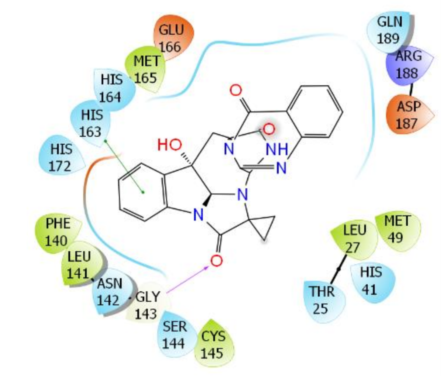
</td></tr>
</table>

9	**M4**	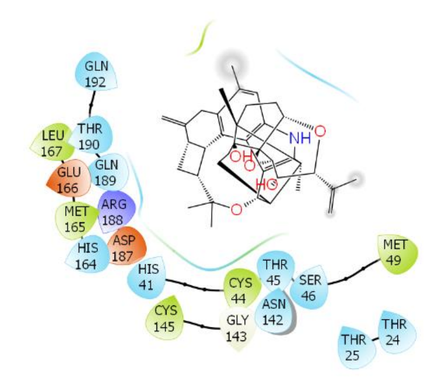	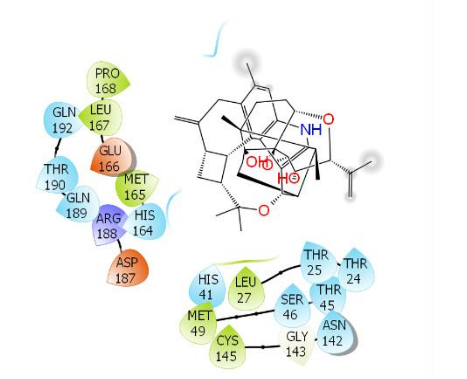
10	**M16**	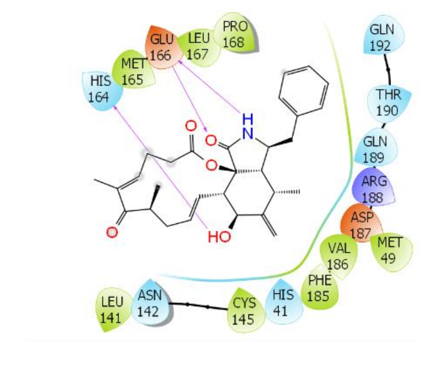	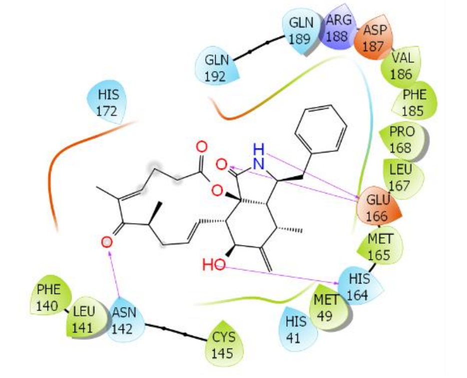
11	**M6**	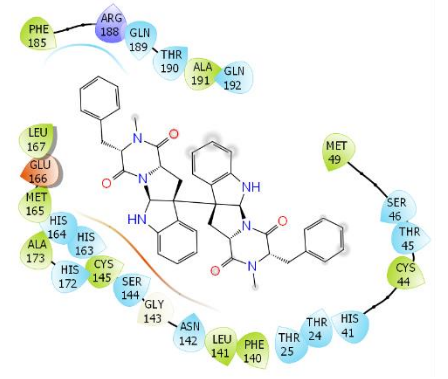	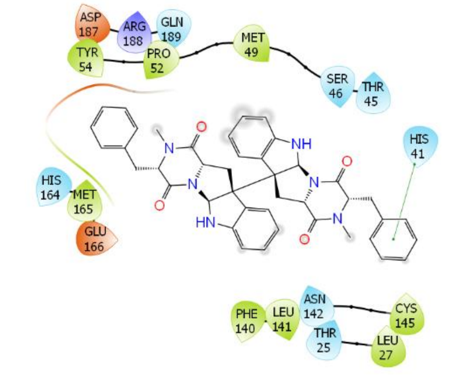
12	**M1**	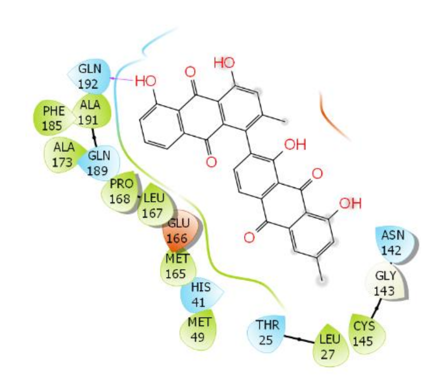	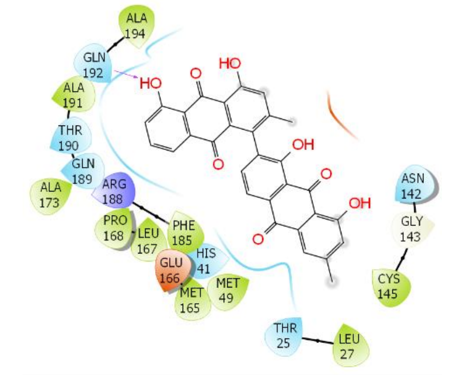

13	**M12**	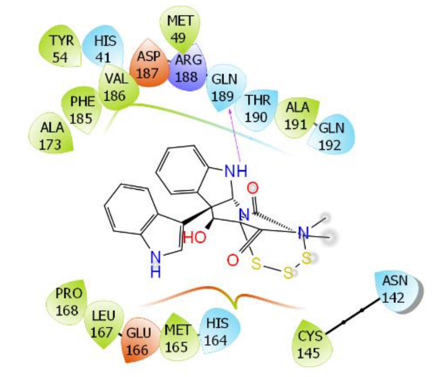	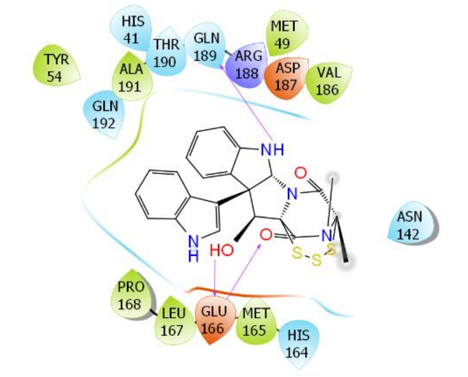
14	**M14**	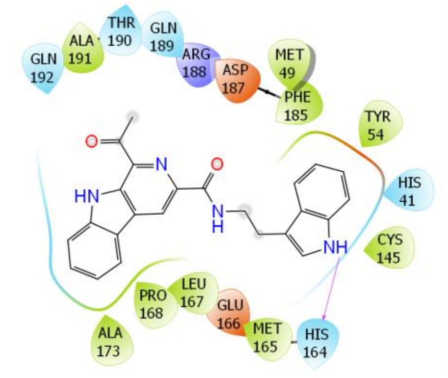	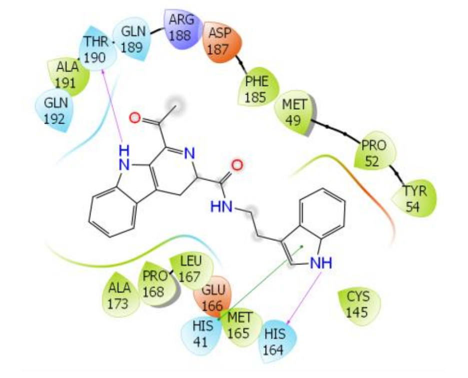
15	**M7**	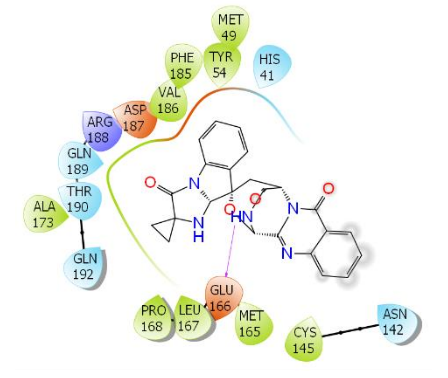	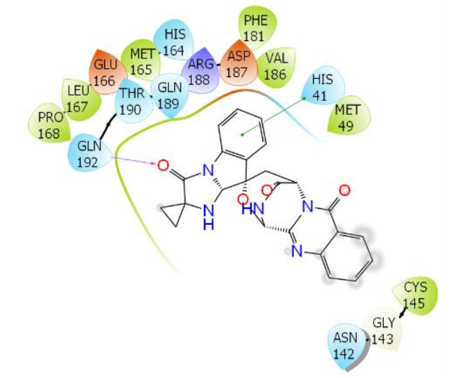
16	**M10**	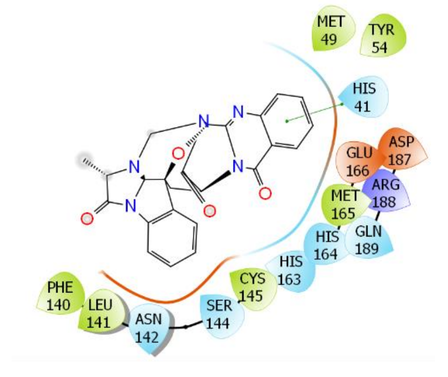	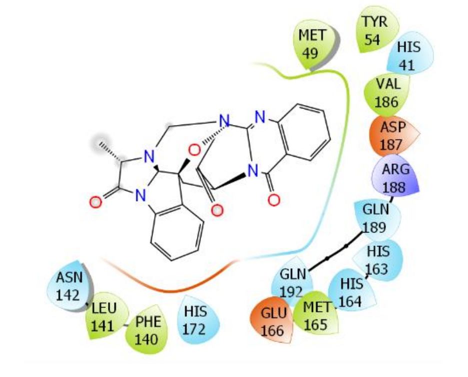

Table S3. The pulling force in displacement dependence over FPL simulations

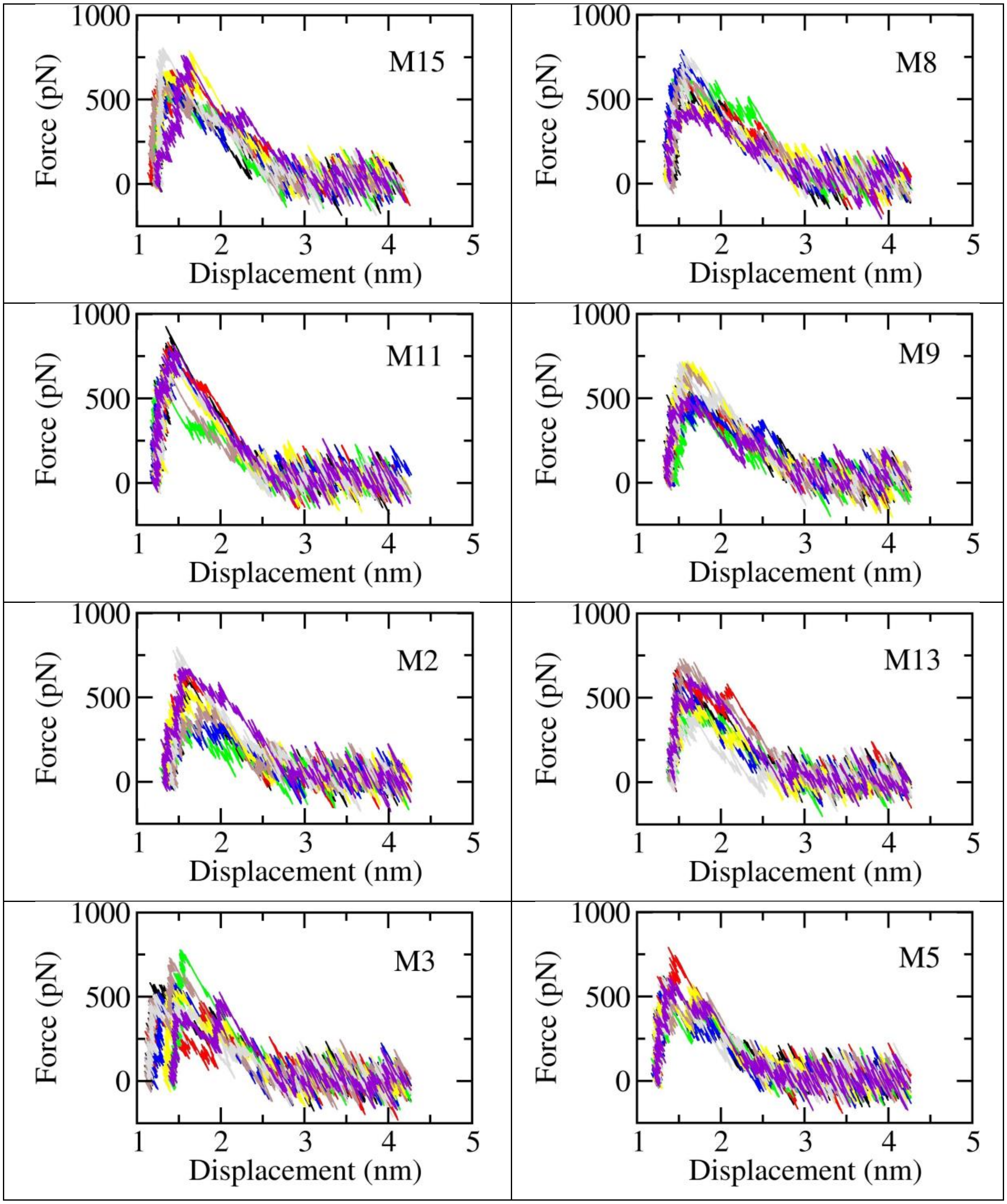

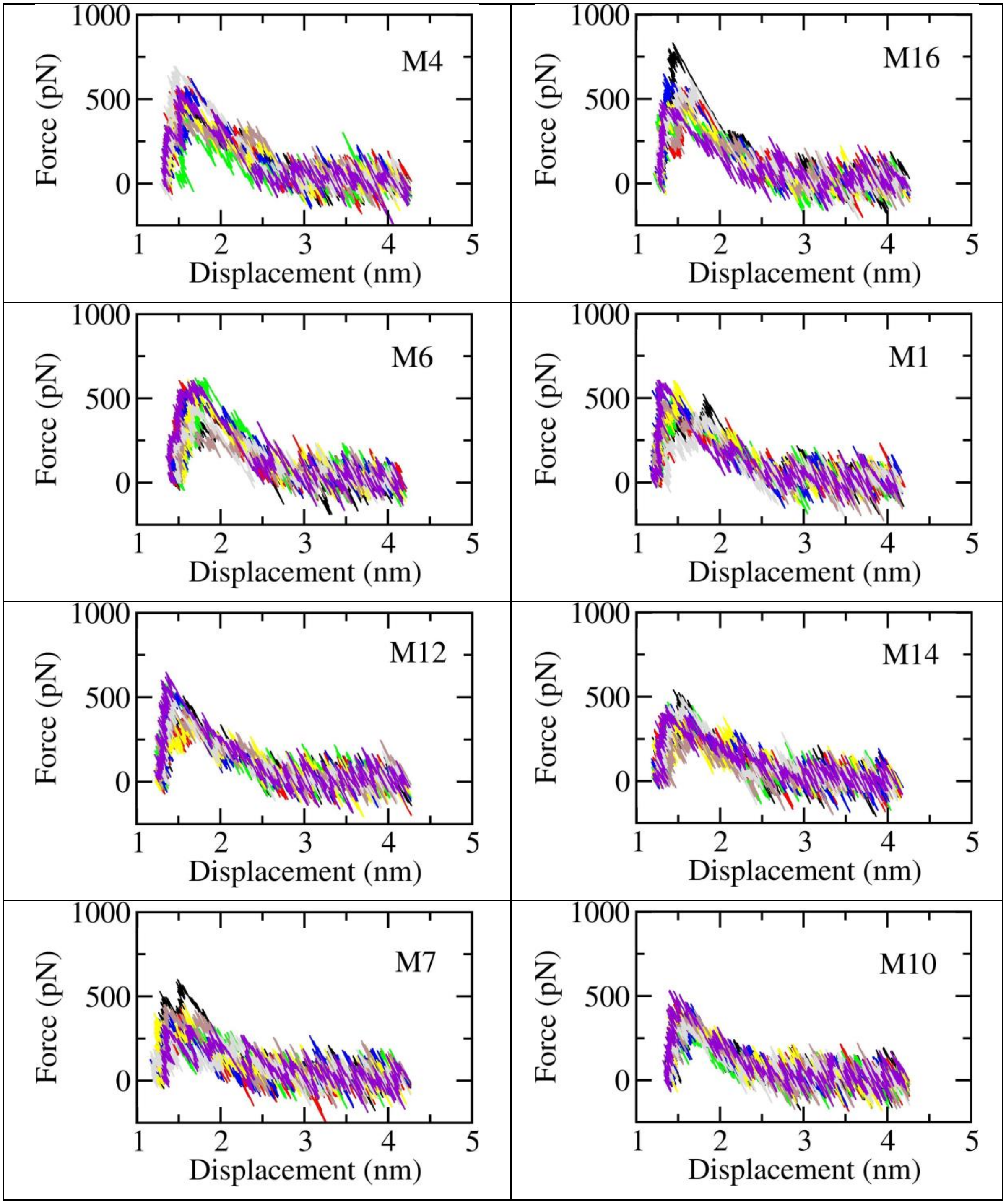
M4
M16
M6
M1
M12
M14
M7
M10
Force (pN)
Displacement (nm)

Table S4. The computational values using FPL simulations of available inhibitors.

N⁰	Compound	F_{Max}	W	ΔG_{EXP}[a]
1	Atazanavir	762.8 ± 44.8	83.7 ± 3.5	-8.64
2	Candesartan Cilexetil	682.6 ± 15.7	68.2 ± 2.3	-9.23
3	Chloroquine	417.3 ± 40.9	34.5 ± 2.8	-8.56
4	Cimetidine	513.0 ± 29.7	46.5 ± 3.9	-7.51
5	Maribavir	377.0 ± 19.4	33.9 ± 1.5	-7.51
6	Omeprazole	361.4 ± 31.1	28.2 ± 2.4	-8.03
7	Oxytetracycline	448.1 ± 30.7	37.9 ± 2.7	-8.22
8	Roxatidine Acetate Hydrochloride	652.2 ± 31.7	62.2 ± 3.3	-8.05
9	Sulfacetamide	282.6 ± 19.0	16.0 ± 1.4	-7.51
10	Valacyclovir Hydrochloride	492.8 ± 20.9	49.8 ± 3.3	-8.16

[a]The experimental binding free energy was obtained through inhibition constant k_{i}.[1] The unit of energy and force are in kcal mol^{-1} and pN, respectively.

Table S5. ADME profile of potential compounds

Compound	Molecular Weight	HIA	BBR	Carcinogenicity	LD_{50} (mg/kg)	Toxicity prediction[a]
**M8**	722.18	0.6252	0.5804	0.9571	300	3
**M9**	722.18	0.6252	0.5804	0.9502	300	3
**M11**	429.14	0.6725	0.6995	0.7880	75	3
**M15**	552.15	0.9105	0.9401	0.9717	5000	5

[a]Toxicity prediction class: 1 → 6 (High toxicity to non-toxic). The ADME was calculated via admetSAR[2] and ProTOX-II[3] webserver.

Reference

1. Z. Li, X. Li, Y.-Y. Huang, Y. Wu, R. Liu, L. Zhou, Y. Lin, D. Wu, L. Zhang, H. Liu, X. Xu, K. Yu, Y. Zhang, J. Cui, C.-G. Zhan, X. Wang and H.-B. Luo, *Proc. Natl. Acad. Sci. U.S.A*, 2020, **117**, 27381-27387.
2. F. Cheng, W. Li, Y. Zhou, J. Shen, Z. Wu, G. Liu, P. W. Lee and Y. Tang, *J. Chem. Inf. Model.*, 2012, **52**, 3099-3105.
3. P. Banerjee, A. O. Eckert, A. K. Schrey and R. Preissner, *Nucleic Acids Res* 2018, **46**, W257-W263.
